# Experimental and Computational Elucidation of C(*sp* ^3^)–H Fluorination Barriers in an Iron(II)- and 2‑Oxoglutarate-Dependent Halogenase

**DOI:** 10.1021/jacs.6c03646

**Published:** 2026-07-30

**Authors:** Vishal Yadav, Chao Wang, Christopher J. Pollock, Jiang Ren, Evan J. Burke, Chi-Yun Lin, Jeffrey W. Slater, Xiaojun Li, Irene Schaperdoth, Wei-Chen Chang, Elvira R. Sayfutyarova, Alexey Silakov, Carsten Krebs, J. Martin Bollinger

**Affiliations:** † Department of Chemistry, 8082 The Pennsylvania State University, University Park, Pennsylvania 16802, United States; ‡ Cornell High Energy Synchrotron Source, Wilson Laboratory, 5922 Cornell University, Ithaca, New York 14853, United States; § Department of Chemistry, 6798 North Carolina State University, Raleigh, North Carolina 27695, United States; ∥ Department of Biochemistry and Molecular Biology, 8082 The Pennsylvania State University, University Park, Pennsylvania 16802, United States

## Abstract

Incorporation of fluorine into pharmaceuticals, agrochemicals, and molecular-imaging agents is of growing importance. Multiple synthetic fluorination methods have recently emerged, and metalloenzymes that are potentially capable of C­(*sp* ^3^)–H fluorination have been reported. Nevertheless, direct, regioselective fluorination of aliphatic carbon centers remains an unsolved problem. Here, we show for the iron­(II) and 2-oxoglutarate-dependent (Fe/2OG) l-lysine 4-chlorinase, BesD, which might be envisaged to support C­(*sp* ^3^)–H fluorination by the direct cognate of its native chlorination mechanism, that the enzyme can (1) coordinate F^–^ at its Fe­(II) cofactor, (2) activate O_2_ to form a *cis*-Fe^IV^(O)­(F) (fluoroferryl) intermediate, and (3) use the intermediate to abstract hydrogen from its substrate. In what would be the key final step, fluorine (F•) coupling to the substrate radical is unable to compete with the hydroxyl-radical (HO•) “rebound” step characteristic of related hydroxylases. Electron paramagnetic resonance (EPR) and X-ray absorption spectroscopic (XAS) data establish that fluorine remains bonded to the iron cofactor through steps 1–3 and therefore available for transfer to the substrate radical. QM/MM calculations suggest that the F•-coupling step is associated with an activation barrier considerably higher than that of HO• rebound, consistent with the observed outcome. The findings experimentally verify prior proposals that the impediment to C­(*sp* ^3^)–H fluorination by the canonical mechanism of an Fe/2OG halogenase lies solely in the final radical-coupling step and set the stage for exploration of whether a potentially surmountable geometric barrier or an insurmountable electronic one is primarily responsible.

## Graphical abstract

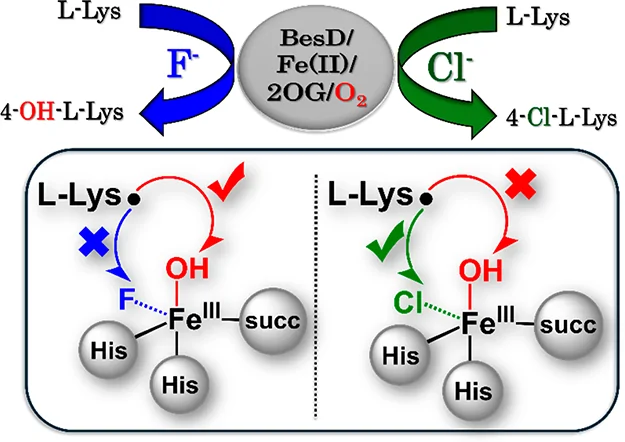

## Introduction

Fluorinated organic compounds are widely used as clinical diagnostics and pharmaceuticals (Scheme 1A).^1^ The presence of fluorine confers significant advantages to drugs in their pharmacokinetics, to materials in their physical properties and stabilities,^2^ to agrochemicals in their environmental persistence,^3,4^ and to catalysts in their reactivity and robustness.^5^ In recent years, an estimated 20% of the drugs and 50% of the agrochemicals that have reached the marketplace contain fluorine.^1,6^ As of 2018, almost half of the small-molecule drugs approved by the Food and Drug Administration (FDA) were fluorine-bearing compounds, and nine of 50 drugs approved in 2021 contain fluorine.^7^ The presence of fluorine in small molecules increases their metabolic stability and modulates their conformation, acidity, intrinsic potency, and membrane permeability. Additionally, ^18^F is extensively used in positron emission tomography (PET) because of its convenient half-life (*t*_*1/2*_ = 110 min) and amenability to high-resolution images.^8^ Consequently, methods for efficient C–^18^F bond formation (e.g., in [^18^F]-fluorodeoxyglucose) are in high demand.

**Scheme 1:**
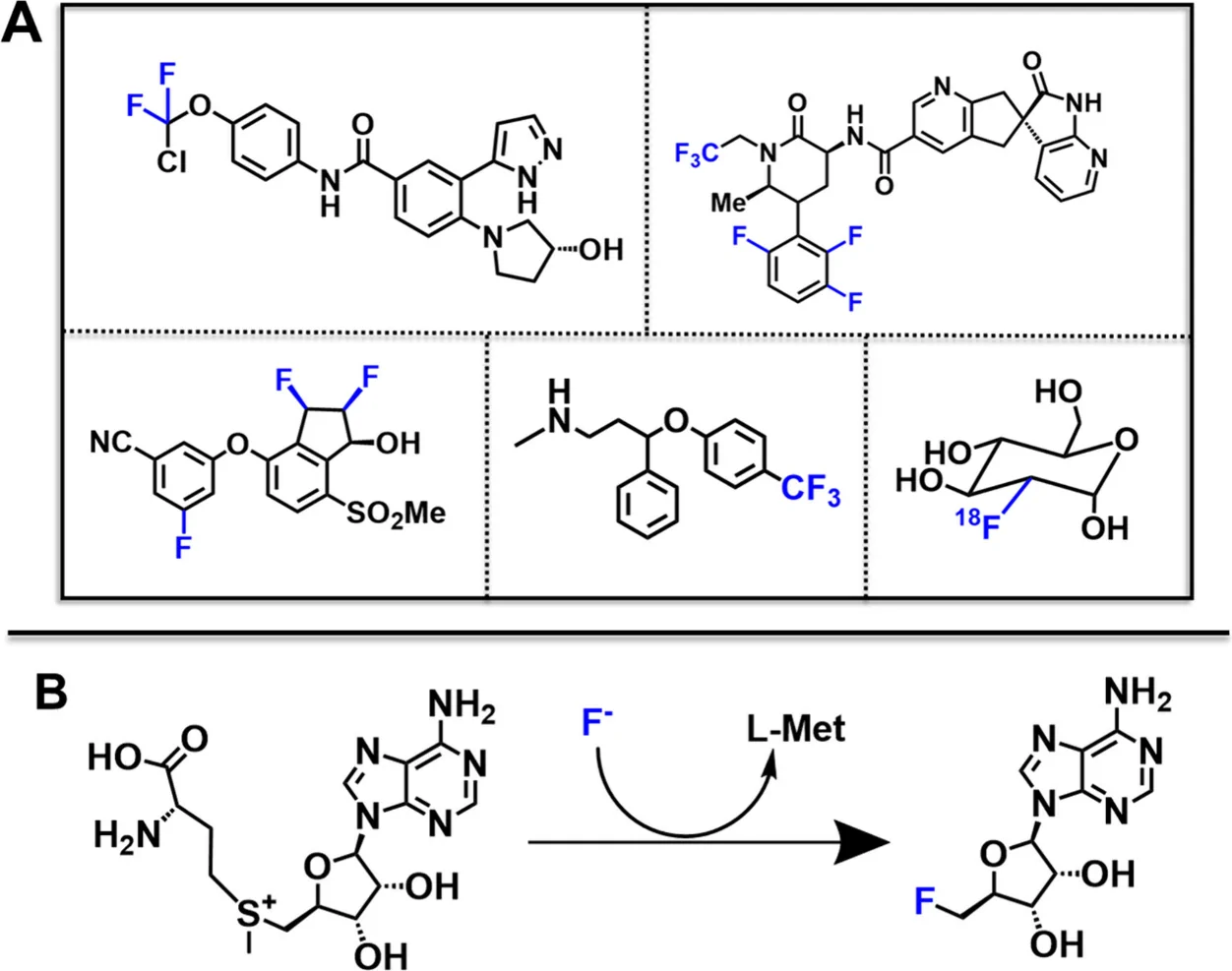
(A) FDA Approved Fluorine-Containing Drugs, (B) SAM-Based Enzymatic C­(*sp* ^3^)–H Fluorination

The unique properties of fluorine—its small size (1.47 Å), highest inherent electronegativity (3.98 on the Pauling scale), ability to form very strong (472 kJ/mol) bonds to carbon, and high reactivity in its elemental form (F_2_)—pose significant challenges for synthetic chemists seeking to develop methods to incorporate it into organic molecules. Additionally, the introduction of fluorine substantially changes the electronic structures (HOMO and LUMO energies) and reactivities of organic molecules, complicating intuition of postfluorination steps in synthetic schemes in which the halogen is installed early.^9^ Although several chemical methods to install C–F bonds are known,^10–19^ they are generally limited by requirements for some combination of a preactivated carbon center, an expensive fluorine donor, or a regioselectivity-directing group. Many also generate harmful waste products.^20^ Enzymatic C–H fluorination reactions could conceivably obviate these liabilities. In biosynthetic pathways,^21–26^ metalloenzymes install C–C,^27,28^ C–S,^29^ C–Cl/Br,^30,31^ C–O^32–34^ and C–N^35,36^ bonds to aliphatic, aromatic, and olefinic carbons in transformations that, on their surface, seem adaptable to the use of fluorine. Nevertheless, with the exception of one S-adenosyl-l-methionine (SAM) based fluorinase (Scheme 1B), which catalyzes a nucleophilic attack by F^–^, there are no known enzymes that effect C–F bond formation in their native reactions.^37^

Iron­(II)- and 2-oxoglutarate-dependent (Fe/2OG) aliphatic halogenases are found in diverse organisms, including bacteria, fungi, plants, and marine animals, where they catalyze reactions within biosynthetic pathways to natural products with potent biological activities.^38^ In an Fe/2OG halogenase, the Fe­(II) center is coordinated by two histidine (His) imidazoles and a halide ion (usually Cl^–^).^30,39–42^ In the generally accepted halogenation mechanism (Scheme 2),^43,44^ the Fe­(II) cofactor captures O_2_ to form a superoxoiron­(III) intermediate, which converts to a succinate-coordinated *cis*-Fe^IV^(O)­(Cl) (chloroferryl) complex by oxidative decarboxylation of the 2OG cosubstrate. The chloroferryl complex abstracts hydrogen (H•) from the substrate (R–H), producing a state with a *cis*-Fe^III^(OH)­(Cl) cofactor and carbon radical. This state can undergo either chlorine-radical (Cl•) transfer to give the chlorinated product (R–Cl) or hydroxyl-radical (HO•) transfer (known as “rebound″^45^) to give the alcohol (R–OH). Halogen transfer can occur with high (>95%) selectivity over rebound, though most characterized halogenases still mediate some hydroxylation.^39,46,47^ Avoiding the rebound step to allow Cl• coupling step to prevail is the central mechanistic imperative for halogenases, and the manner in which it is achieved has been extensively studied experimentally^14,48–62^ and computationally^63–68^ in both enzymes and inorganic model systems. Substrate-cofactor disposition^69,70^ and coordination dynamics^40,71,72^ have been suggested as controlling factors. In the past decade, the Fe/2OG halogenases have drawn interest from the biocatalysis community for their abilities to (1) install good leaving groups potentially suitable for late-stage fragment couplings, (2) promote diverse non-native reactions (Scheme 3) and (3) accommodate a wide structural range of substrates.^73–77^ Despite progress in utilizing Fe/2OG halogenases for synthetic purposes,^78–80^ aliphatic C­(*sp* ^3^)–H fluorination by a member of this enzyme class has not yet been demonstrated.

**Scheme 2:**
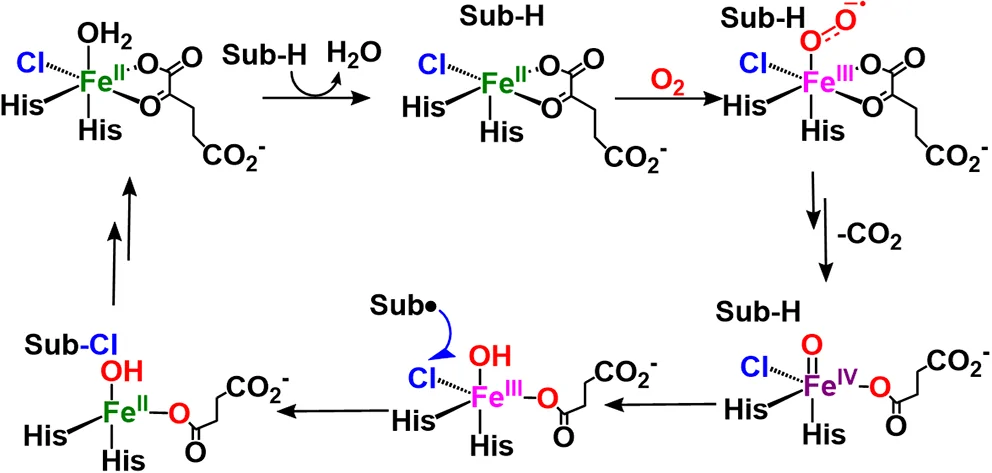
General Mechanism of Fe/2OG Halogenases

**Scheme 3:**
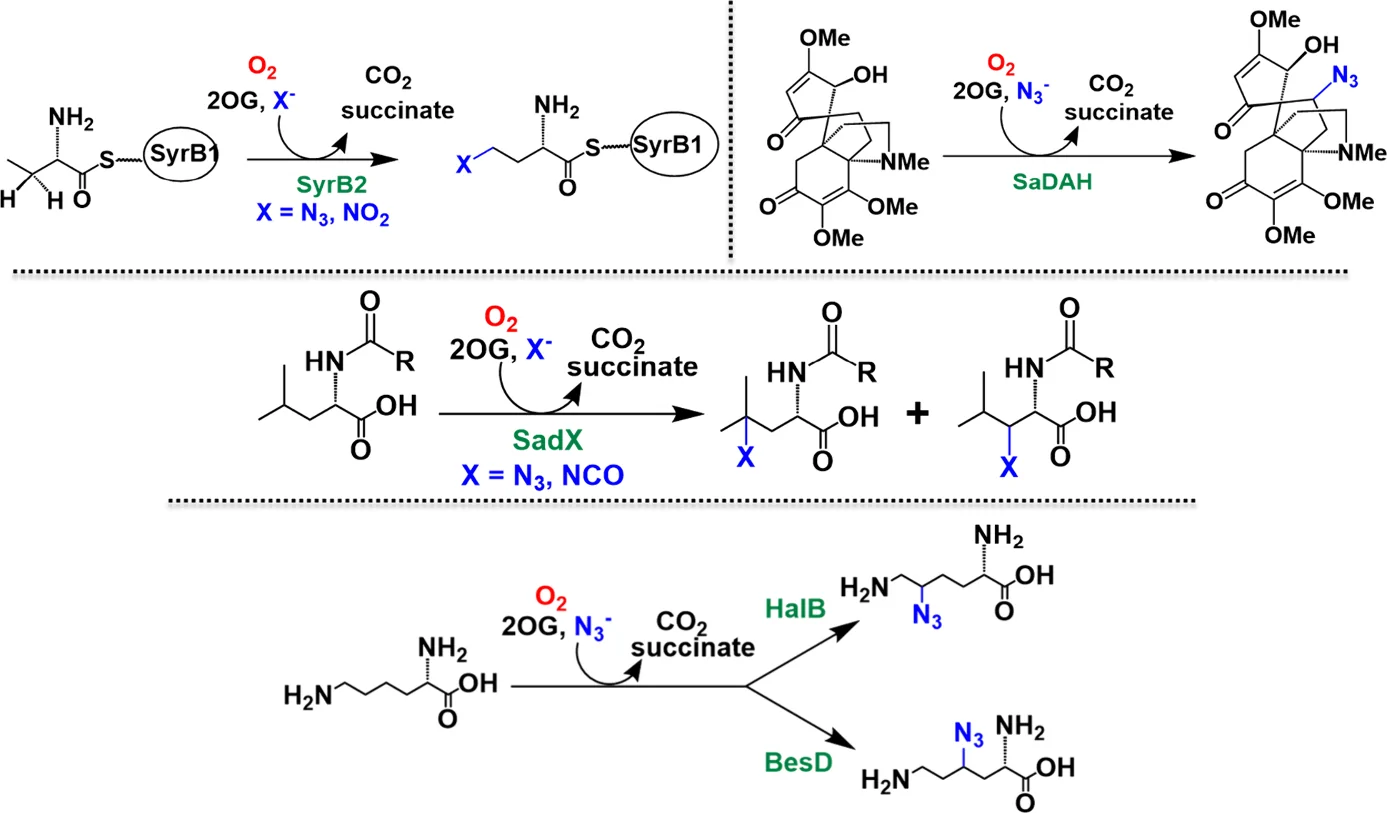
Non-Native Reactions Catalyzed by Fe/2OG Halogenases^39,47,85,98^

A recent computational study^81^ suggested that, for the Fe/2OG halogenases, the identity of the halide has a minimal effect on the barrier for hydrogen atom transfer (HAT) from the substrate to the *cis*-haloferryl intermediate [*cis*-Fe^IV^(O)­(X)]. This study also predicted that halogen transfer from the resultant *cis*-halo-hydroxoferric complex should have a considerably higher barrier for F than for the larger halogens, Cl and Br, potentially precluding fluorination by the canonical halogenase mechanism. The view that formal coupling of an Fe­(III)-coordinated fluoride to a carbon radical could have a prohibitively high barrier has recently been challenged, however, by separate studies of Huang^82^ and Yang,^83^ who reported enzyme mediated C–F-bond formation by evolved versions of the nonheme-iron enzymes, (*S*)-2-hydroxypropylphosphonate epoxidase (HppE) and 1-aminocyclopropane-1-carboxylic acid oxidase (ACCO) (Scheme 4). In both studies, the unnatural substrate contained an activated N–F moiety that the authors proposed to transfer F• to the Fe­(II) cofactor, generating an aminyl radical along with the fluoroferric cofactor. This radical, they proposed, generates a carbon-centered radical (R•) by HAT. The R• then couples to the coordinated fluorine (Scheme 4). Although these studies showed that radical R• ↔ •F coupling involving an Fe­(III)-coordinated fluoride is feasible, their use of a preactivated fluorine donor represents a major limitation relative to the coupling of free fluoride that can be envisaged to occur via the high-valent metal-oxo-mediated C–H-functionalization strategy of the Fe/2OG enzymes. C­(*sp* ^3^)-H fluorination initiated by metal-oxo complexes thus remains an untapped synthetic strategy that would represent a significant breakthrough in the field of biocatalysis.

**Scheme 4:**
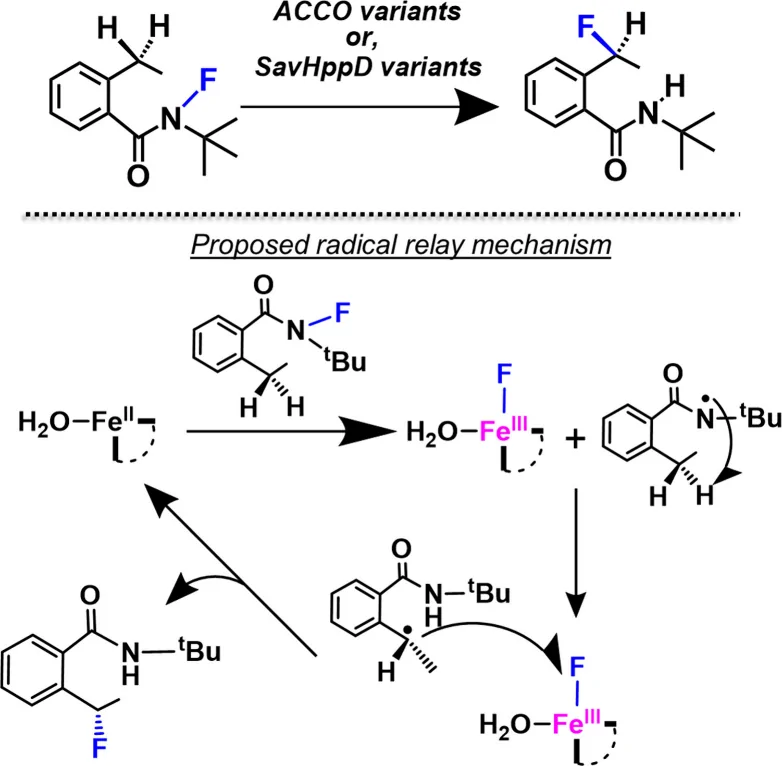
Radical Fluorination Using Nonheme Iron Enzymes and Their Proposed Mechanism

In 2019, Marchand et al. reported discovery of an Fe/2OG halogenase, BesD, that effects selective chlorination of C4 of l-lysine (l-Lys) to produce 4-Cl-l-Lys.^39,84^ In a later study, Smithwick et al. showed that BesD can also promote bromination (to produce 4-Br-l-Lys) and azidation (to produce 4-N_3_-l-Lys) when supplied with the appropriate anion.^85^ In this study, when the BesD reaction was carried out in the presence of excess F^–^, it produced exclusively the alcohol product (4-OH-l-Lys), with no trace of the corresponding fluorinated species (4-F-l-Lys) (Scheme 5). This observation is consistent with work of Lewis and co-workers, who elicited chlorination and bromination from evolved versions of the hydroxylase, SadA,^76^ but saw only hydroxylation in the presence of F^–^.^86^ To date, no experimental analysis of the failure of an Fe/2OG halogenase to install fluorine has been reported. In the absence of such an analysis, it remains possible that the barrier could arise from any one of the four major steps in the halogenation sequence. In other words, (1) poor F^–^ binding/coordination due to high hydration energy of fluoride in aqueous medium,^87^ (2) a failure of the fluoride-coordinated cofactor to activate O_2_ ^55^ and form the *cis*-Fe^IV^(O)­(F) complex, (3) insufficient stability or H•-abstraction potency of the *cis*-Fe^IV^(O)­(F) complex, or (4) a failure of F• to be transferred to the substrate radical (as favored by Kulik and Lewis^86^) could be responsible for the failure of Fe/2OG-halogenase-mediated fluorination.

**Scheme 5:**
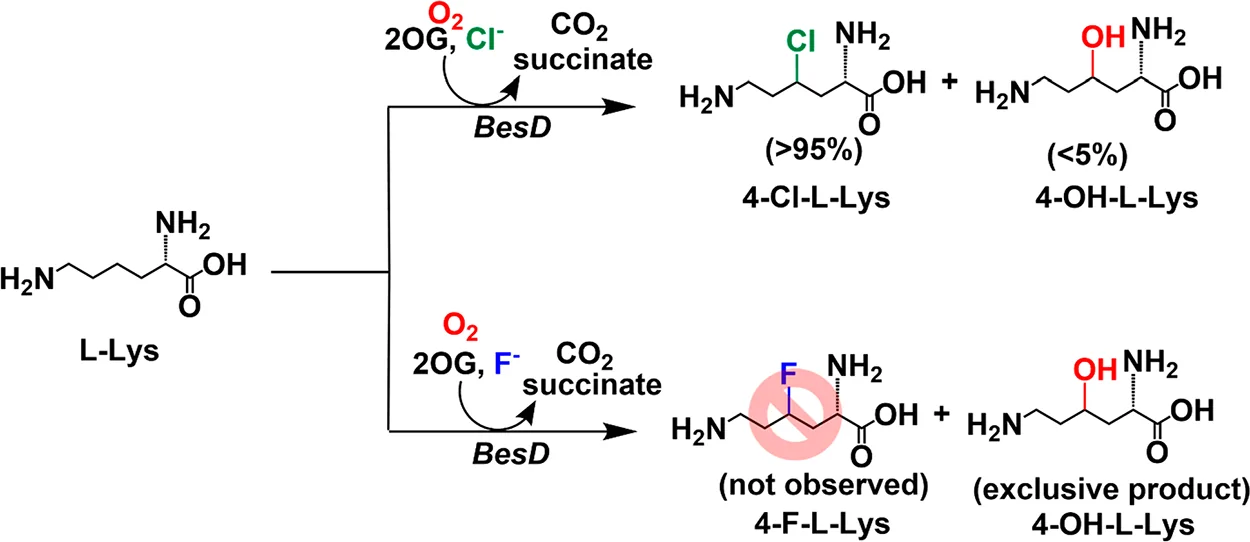
Anion Mediated Reaction Selectivity in BesD Halogenase

In this work, we used kinetic and spectroscopic methods to dissect the reaction of BesD in the presence of fluoride to address the enzyme’s apparent inability to couple fluorine to the target carbon (C4) of its prime substrate, l-Lys. The data show that (1) the reactant complex with fluoride in place of chloride can readily form, (2) this complex can activate dioxygen to form a fluoroferryl complex, and (3) the fluoroferryl complex can abstract hydrogen from the substrate. The formation of only alcohol product(s) thus implies that the sole barrier to realization of enzymatic fluorination by this strategy is the previously recognized competition between oxygen rebound and the desired halogen coupling step.^70,86,88^ Additional computational analysis sets the stage for efforts to modify the enzyme or substrate to enforce a geometry allowing R• ↔ •F coupling to compete.

## Results and Discussion

### Dependence of BesD Products on Added Anion

As expected from previous studies,^39,84^ analysis of the products from reactions containing BesD, Fe­(II), 2OG, l-Lys, chloride and O_2_ by liquid chromatography and mass spectrometry (LCMS) (Figure 1) revealed peaks at *m*/*z* values of 181.1 and 183.1 (in positive-ion mode) characteristic of 4-Cl-l-Lysine (Figure 1A; *green traces*). By contrast, we found that otherwise equivalent reactions containing fluoride in place of chloride formed exclusively a product with *m*/z = 163.1, characteristic of a hydroxylated species (*red trace*); we could not definitively detect a peak at the *m*/*z* value of 165.1 expected for a fluorinated product (Figure S1). In these reactions with fluoride, use of ^13^C_6_^15^N_2_-l-Lys shifted the primary peak to *m*/*z* = 171.1 (Figure S2), confirming that the product is derived from the “prime″ substrate, l-Lys. Comparisons of LCMS traces obtained from reactions with 4,4-[^2^H_2_]- and 5,5-[^2^H_2_]-l-Lys were performed in the presence of fluoride to probe the regioselectivity of hydroxylation. Reactions containing 4,4-[^2^H_2_]-l-Lys produced a mixture of 5-OH-4,4-[^2^H_2_]-l-Lysine (*m*/*z* 165.1) and 4-OH-4-[^2^H]-l-Lys (*m*/*z* 164.1) (Figure S3), whereas reactions with 5,5-[^2^H_2_]-l-Lys yielded exclusively 4-OH-5,5-[^2^H_2_]-l-Lysine (*m/z* 165.1) (Figure S4). Furthermore, reactions with 4,4,5,5-[^2^H_4_]-l-Lys produced a single product peak with a retention time corresponding to the C4-hydroxylated product (R_t_ = 15.9 min), rather than the C5-hydroxylated product (R_t_ = 16.3 min). Collectively, these data demonstrate that, even in the presence of fluoride, C4 remains the primary site of functionalization, consistent with the native chlorination regioselectivity of the enzyme. Omission of Fe­(II), 2OG, l-lysine or O_2_ from the fluoride-containing reactions eliminated or drastically suppressed the peak for 4-OH-l-Lys (Figure 1B), and use of ^18^O_2_ resulted in development of a new peak at *m/z* = 165.1, a shift of +2 relative to the *m*/*z* of the product formed with natural-abundance dioxygen (Figure 1C). Use of either ^18^O_2_ or H_2_^18^O in place of their natural-abundance forms gave peaks at both 163.1 and 165.1, implying hydroxylation by an HO•-rebound mechanism but with some solvent exchange of the iron-bound oxygen ligand, as is well-documented in other Fe/2OG enzyme reactions.^89^ (Figure S20). Additionally, in the reactions with fluoride, we did not detect the 2-amino-4-propylamine-γ-lactone/2-amino-4-hydroxy-ε-lactam products previously found to form from the native 4-Cl-Lys BesD product (Figure S21). This observation provides further evidence that the detected 4-OH-l-Lys product does not result from hydrolytic decomposition of an initially halogenated product, as was seen in the case of the native chlorination reaction.^39^ A screen of other anions showed that fluoride is uniquely potent in enhancing production of the alcohol product (Figure S5). Different fluoride salts (NaF, KF, CsF) (Figure S6) afforded this enhancement, establishing that the effect arises from the anion. Attempts to replace the Fe­(II) cofactor with a different transition metal failed to yield fluorinated or hydroxylated product(s) (Figure S7). The results show that BesD can promote (pseudo)­halogenation reactions with chloride, bromide, or azide, but not with fluoride.

**Figure 1:**
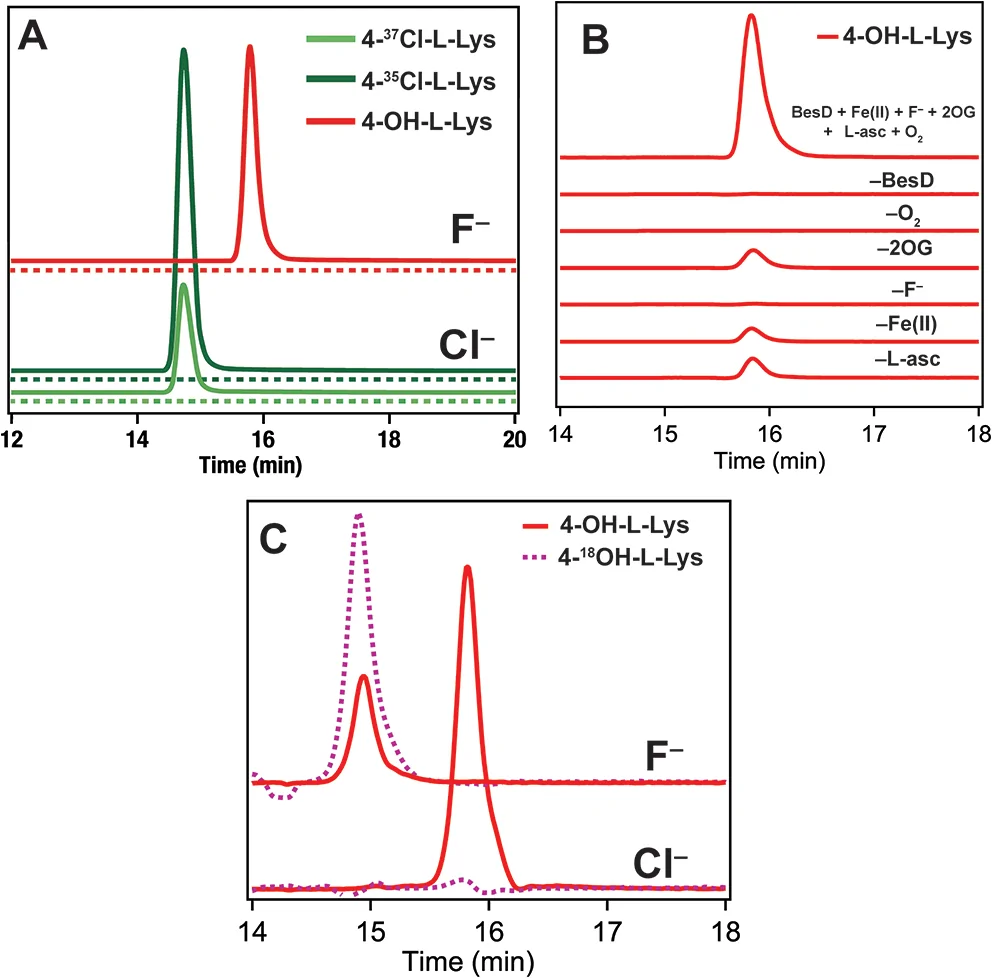
LCMS characterization (single-ion chromatograms) of the (A) 4-OH-l-lysine product (*m*/*z* = 163.1, red trace) and 4-Cl-l-lysine product (*m*/*z* = 181.1­(^35^Cl) and 183.1 (^37^Cl), green trace) formed by adding air saturated buffer (100 mM HEPES, pH 7.5) to BesD•Fe­(II)•l-Lys•F^–^ and BesD•Fe­(II)•l-Lys•Cl^–^ in the presence (solid trace) and absence (dotted trace) of 2OG; (B) 4-OH-l-Lysine product (*m*/*z* = 163.1) under different reaction conditions, establishing enzymatic hydroxylation; (C) LCMS characterization (single-ion chromatogram) of 4-OH-l-lysine (*m*/*z* = 163.1, red solid lines) and 4-^18^OH-l-lysine (*m*/*z* = 165.1, magenta dotted lines) formed by adding ^18^O_2_ saturated buffer (100 mM HEPES, pH 7.5) to BesD•Fe­(II)•2OG•l-Lys•F^–^ and BesD•Fe­(II)•2OG•l-Lys•Cl^–^.

Monitoring production of succinate provided a measure of the robustness of the reaction carried out in the presence of either the native chloride or the non-native fluoride anion. Under conditions of low turnover numbers, succinate yields were similar with the two anions (Figure S8, right), suggesting that the initial, ferryl-forming events are unimpeded by the non-native halide. However, under these conditions, the combined area of the two LCMS peaks of the 4-Cl-l-Lys product with the native anion was ∼ 8 times that of the peak for the 4-OH-l-Lys product in the presence of fluoride (Figure S8, left), suggestive of uncoupled 2OG consumption in the presence of fluoride. In addition, fluoride supported fewer turnovers (∼10) than chloride (∼ 60) at high l-Lys concentrations (Figure S9). These observations suggested that the reaction with fluoride could have multiple points of inefficiency or failure and led us to dissect the reaction to evaluate each step for compromise by the smaller halide.

### Evidence for Ligation of F^–^ to the Fe­(II) Cofactor by Visible Absorption Spectroscopy

We showed in prior work that BesD binds l-Lys and the iron-coordinating exogenous anion (Cl^–^, Br^–^, N_3_^–^) with strong heterotropic cooperativity, which is most likely related to their opposite charges.^85,90^ We anticipated that F^–^ binding/coordination would exhibit the same thermodynamic linkage to l-Lys binding and tested for it by monitoring the absorption spectrum arising from the metal-to-ligand charge-transfer (MLCT) transition of the Fe­(II)•2OG complex. As seen previously with other (pseudo)­halides, addition of F^–^ to the Fe­(II)•BesD•2OG ternary complex caused no change in the absorption spectrum (Figure S10, green trace). Similarly, addition of l-Lys by itself failed to perturb the spectrum. By contrast, addition of both F^–^ and l-Lys induced a significant bathochromic shift in the MLCT band (Figure S10, blue trace), indicating that F^–^ and l-Lys bind together with the heterotropic cooperativity seen with other anions.

### Evidence for Ligation of F^–^ to the Fe­(II) Cofactor by ^57^Fe Mössbauer Spectroscopy

We obtained further evidence of F^–^ binding/coordination by ^57^Fe Mössbauer spectroscopy. Frozen samples containing BesD, ^57^Fe­(II), 2OG, l-Lys and fluoride (from either CsF or NaF) exhibited a quadrupole doublet with an isomer shift (δ) of 1.12 mm/s and a quadrupole splitting parameter (ΔE_Q_) of 2.28 mm/s. These parameters are significantly different from those associated with the BesD•^57^Fe­(II)•2OG complex formed in the presence of l-Lys but absence of added anion (δ = 1.24 mm/s , |ΔE_Q_| = 2.94 mm/s), the BesD•^57^Fe­(II)•2OG•l-Lys•Cl^–^ quinary complex (δ = 1.09 mm/s,|ΔE_Q_| = 2.63 mm/s),^90^ or the BesD•^57^Fe­(II)•2OG•l-Lys•Br^–^ complex (δ = 1.08 mm/s,|ΔE_Q_| = 2.58 mm/s). An increase in [F^–^] from 2 mM to 1 M increased the relative area of this new δ = 1.12 mm/s/ΔE_Q_ = 2.30 mm/s quadrupole doublet from ∼ 75% to ∼ 98% (Figure S11), suggesting that the higher concentration is essentially saturating. An overlay of the spectra of the chloride-, fluoride-, and bromide-bound quinary complexes with that of the sample lacking added anion is shown in Figure 2A.

**Figure 2:**
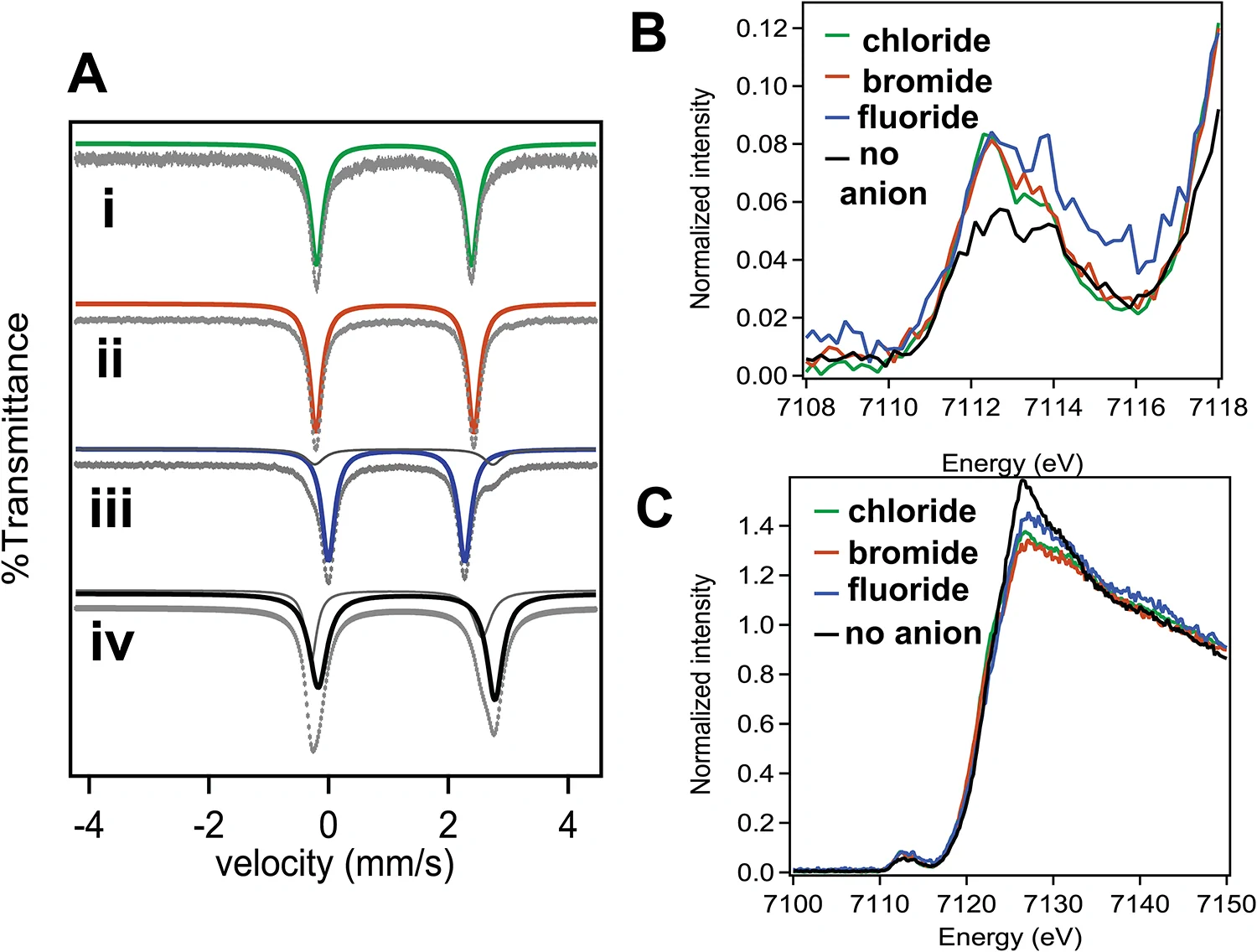
(A) Zero-field ^57^Fe Mössbauer spectra (4.2 K/0 mT) of an anoxic sample containing 2.0 mM BesD, 1.8 mM ^57^Fe^II^, 16 mM 2OG, and either (i) 30 mM l-Lys and 200 mM NaCl; (ii) 60 mM l-Lys and 300 mM NaBr; (iii) 30 mM l-Lys and 200 mM CsF or (iv) 30 mM l-Lys and no anion. The isomer shift (δ_Fe(II)_) and quadrupole splitting (|ΔE_Q,Fe(II)_|) parameters of the Fe^II^ species are δ_Fe(II)_ = 1.09 mm/s, ΔE_Q,Fe(II)_ = 2.63 mm/s for Fe^II^(Cl) (solid green line), δ_Fe(II)_ = 1.08 mm/s, ΔE_Q,Fe(II)_ = 2.58 mm/s for Fe^II^(Br) (solid orange line), δ_Fe(II)_ = 1.12 mm/s, |ΔE_Q,Fe(II)_| = 2.28 mm/s for Fe^II^(F) (solid blue line), and δ_Fe(II)_ = 1.24 mm/s, |ΔE_Q,Fe(II)_| = 2.94 mm/s for Fe^II^(no anion) (solid black line). In each of the above cases, the dotted lines show experimental data and the solid lines represent the best fit. An overlay of the near edge (left, (B)) and pre-edge (right, (C)) of BesD•Fe­(II)•2OG•l-Lys•X^–^ (X = F, Cl, Br) reveals a higher energy and intensity pre-edge for the fluoride adduct as compared to the other species. [BesD] = 2.0 mM, [^57^Fe^II^] = 1.8 mM, [2OG] = 16 mM 2OG, (A) [l-Lys] = 30 mM for F^–^, Cl^–^ and 60 mM for Br^–^, [F^–^/Cl^–^] = 500 mM, [Br^–^] = 300 mM.

### Evidence for Ligation of F^–^ to the Fe­(II) Cofactor by X-ray Absorption Spectroscopy

We further investigated fluoride binding/coordination by K-edge X-ray absorption spectroscopy (XAS). The X-ray absorption near-edge structure (XANES) spectrum of an iron complex arises from excitation of Fe core 1s electrons directly into the 3d orbitals. Therefore, XANES analysis enables interrogation of the composition and relative energies within the d-orbital manifold. Coordination of fluoride to iron is known to perturb the valence orbital composition relative to those in the isostructural chloride and bromide complexes. Fluoride coordination makes the iron center more ionic and confers an appreciably greater effective nuclear charge than coordination of a heavier halide.^91^

For maximal sensitivity to changes induced by fluoride coordination, we collected XAS data in high-energy-resolution fluorescence-detected (HERFD) mode, in which we used a high-resolution X-ray emission spectrometer to select a narrow bandwidth of Kα emission, resulting in increased energy resolution in comparison to conventional fluorescence detection. In the Fe K-edge HERFD XAS spectra of frozen samples containing BesD, Fe­(II), 2OG and either Cl^–^, Br^–^, F^–^, or no anion (Figure 2B,C), the edges have approximately the same energy, suggesting similar effective nuclear charge on the iron in all four cases. Indeed, all four of the XANES spectra are consistent with the energy expected for a protein-bound Fe­(II) ion^92,93^ (Figure 2B**/**C). However, the XANES spectrum of the fluoride adduct is distinctly different from the other spectra, possessing the highest intensity and intensity-weighted average energy (IWAE) of the four (Table 2). Fitting analysis (Figure S12) revealed that the pre-edge feature from the sample with fluoride has both a ∼ 10% greater area and 0.3–0.4 eV higher energy than those from the samples with the other halides or no anion. The differences imply that fluoride contributes to a stronger ligand field and induces lower symmetry when it coordinates.

**Table 1:** Summary of Fe­(II) Experimental and Calculated Mössbauer Parameters

	experimental		calculated	
species	δ (mm/s)	|ΔE_Q_| (mm/s)	δ (mm/s)	|ΔE_Q_| (mm/s)
BesD•Fe(II)•2OG•**Cl** ^**‑**^•l-Lys	1.09	2.63	1.06	2.94
BesD•Fe(II)•2OG•**Br** ^**‑**^•l-Lys	1.08	2.58	1.06	2.74
BesD•Fe(II)•2OG•**F** ^**‑**^•l-Lys	1.12	2.28	1.06	2.66
BesD•Fe(II)•2OG•l-Lys	1.24	2.94	-	-

**Table 2:** Summary of XAS Pre-Edge Fit Parameters

species	pre-edge area^†^	pre-edge IWAE	number of fits
BesD•Fe(II)•2OG•**Cl** ^**‑**^•l-Lys	22.7 (1.0)	7113.1 (0.1)	51
BesD•Fe(II)•2OG•**Br** ^**‑**^•l-Lys	22.4 (1.2)	7113.2 (0.1)	60
BesD•Fe(II)•2OG•**F** ^**‑**^•l-Lys	25.0 (1.1)	7113.5 (0.1)	62
BesD•Fe(II)•2OG•l-Lys	17.8 (0.7)	7113.3 (0.1)	57

To explore the pre-edge energy shift further, we calculated XAS pre-edges for the fluoride, chloride, and bromide complexes in ORCA using TD-DFT (Time-Dependent Density Functional Theory) methods. The calculated pre-edges (Figure S13) reproduce the experimentally observed change in intensity and IWAE between the fluoride complex and the complexes with the heavier halides. Whereas the chloride and bromide complexes are predicted to have identical areas and IWAEs, the fluoride complex is expected to have both ∼ 10% more pre-edge area and a 0.3 eV higher energy, differences that match well with those seen experimentally. Analysis of the individual XAS transitions reveals that the increased IWAE seen for the fluoride complex results from a redistribution of intensity toward the highest energy d orbitals (LUMO+4 and LUMO+5). The LUMO+4 orbitals in the chloride and bromide complexes are nonbonding with respect to the halide, whereas LUMO+4 of the fluoride complex acquires some Fe–F σ* character, resulting in a ∼ 0.25 eV shift to higher energy for the transition to it. Moreover, while the LUMO+5 has qualitatively similar bonding character for all three complexes, it possesses notably greater Fe *n*p character in the fluoride complex (2.8% for F vs 1.2% for Cl and 0.9% for Br), resulting in a highest-energy pre-edge transition that is ∼ 3 times as intense for the fluoride adduct.

### Evidence that F^–^ Remains Ligated after Cofactor Oxidation to Fe­(III)

To establish that F^–^ can remain bound after cofactor oxidation, we exposed the BesD•Fe­(II)•2OG•l-Lys•F^–^ complex to nitric oxide (NO•) to form the {FeNO}^7^ complex, which has been taken as a surrogate for the as-yet-undetected initial superoxoiron­(III) catalytic intermediate.^40,94–96^ We prepared samples by exposing an anoxic solution of either the BesD•Fe­(II)•2OG•l-Lys•Cl^–^ complex or the BesD•Fe­(II)•2OG•l-Lys•F^–^ complex to 0.3–0.5 atm nitric oxide gas and freezing the sample in an EPR tube after 10 min. The continuous wave X-band EPR spectrum of the resultant BesD•{FeNO}^7^•2OG•l-Lys•Cl^–^ complex exhibits an EPR signal with g_eff,y_ = 4.14, g_eff,x_ = 3.89, and g_eff,z_ = 2.00, indicative of a typical {FeNO}^7^ center with *S* = 3/2 electron-spin ground state. The spectrum of the BesD•{FeNO}^7^•2OG•l-Lys•F^–^ complex exhibits a signal at g_eff,y_ = 4.31, g_eff,x_ = 3.72, and g_eff,z_ = 1.98, with apparent ^19^F superhyperfine coupling (A_*y*_ = 336 MHz and A_*x*_ = 176 MHz), implying that fluorine remains bonded after the iron is oxidized. The spectrum of the control sample with no halide has a signal at g_eff,x,y_ = 4.00, g_eff,z_ = 1.99 that is more axial than the signals of the halide-bound complexes (Figure S14). The detection of ^19^F but not ^35^Cl or ^37^Cl superhyperfine coupling was also previously reported for {FeNO}^7^ complexes of photosystem II^97^ and is explained by the greater gyromagnetic ratio of the smaller halogen. To rule out the possibility that the additional features in the spectrum of the fluoride-bound complex result from heterogeneity rather than ^19^F superhyperfine splitting, we measured spectra also at Q-band. In the stronger magnetic field, g-anisotropy is accentuated as a result of the increased Zeeman splitting. By contrast, nuclear superhyperfine coupling is essentially independent of the magnetic field. The observation that the coupling is less obvious at Q-band (Figure 3, *compare top-right and bottom-right spectra*) than at X-band is consistent with attribution of the splitting of the major low-field feature to strong ^19^F superhyperfine splitting. Control experiments revealed no such splitting in the spectra of complexes lacking fluoride nor in samples lacking BesD (Figures S15–S16), further corroborating the conclusion that the small non-native halide remains coordinated after NO-mediated oxidation of the iron cofactor.

**Figure 3:**
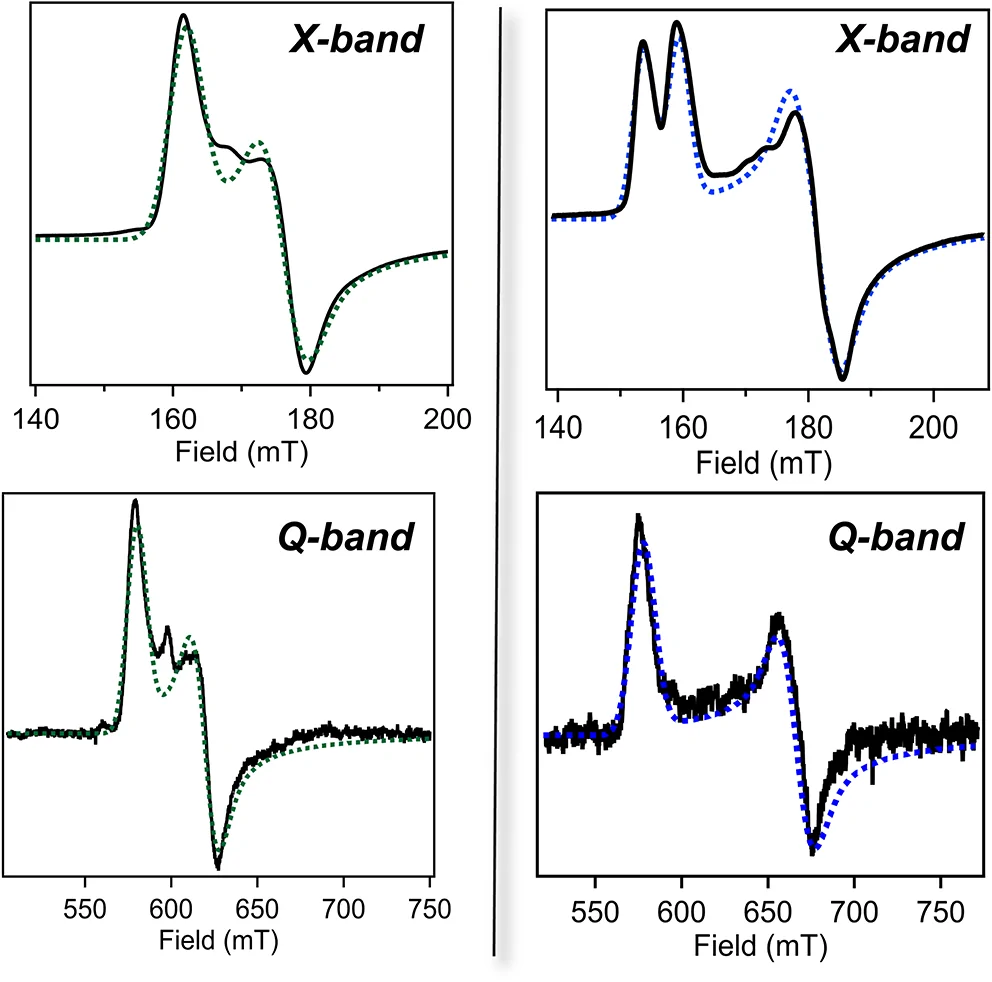
Continuous-wave, X-band (top row) and Q-band (bottom row) EPR spectra of the {Fe-NO}^7^ form of BesD in complexes with l-Lys, 2OG and F^–^ (right) or Cl^–^ (left). Experimental spectra are shown as black lines and simulations are shown as colored dashed lines.

### Evidence for Fluoroferryl Intermediate Accumulation by Stopped-Flow Absorption Spectroscopy

Previous studies on Fe/2OG halogenases have shown that halide coordination activates the Fe­(II) cofactor to capture dioxygen, leading to ferryl intermediate formation.^98,99^ To test whether fluoride binding similarly triggers O_2_ activation in BesD, we performed stopped-flow absorption experiments. Upon rapid mixing of a solution of the BesD•Fe­(II)•2OG•l-Lys•F^–^ complex with a solution containing limiting oxygen, we observed a transient absorption signal at 318 nm (Figure 4, blue trace), which in prior work on BesD and other Fe/2OG oxygenases has been diagnostic of the accumulation and subsequent decay of ferryl intermediates.^96,100^ Notably, in the analogous mixing of the BesD•Fe­(II)•2OG•l-Lys complex (lacking F^–^) with limiting oxygen, we observed no comparable transient 318 nm absorption (Figure 4, red trace), suggesting that halide coordination is essential for the efficient oxygen activation that leads to intermediate accumulation. Comparison of the kinetic traces from the reactions of the chloride- and fluoride-bound quinary complexes with O_2_ reveals that the presumptive fluoroferryl complex (proven below) forms ∼7 times less rapidly than the chloroferryl intermediate (*k*_form,Cl_ = 4.8 s^–1^ and *k*_form,F_ = 0.7 s^–1^). Relative to chloride, fluoride coordination thus slows either O_2_ capture or subsequent steps leading to ferryl formation (e.g., 2OG decarboxylation, O–O-bond cleavage). Additionally, decay of this presumptive fluoroferryl complex is also approximately four times slower than decay of the corresponding chloroferryl intermediate (*k*_decay,Cl_ = 0.8 s^–1^ and *k*_decay,F_ = 0.2 s^–1^).

**Figure 4:**
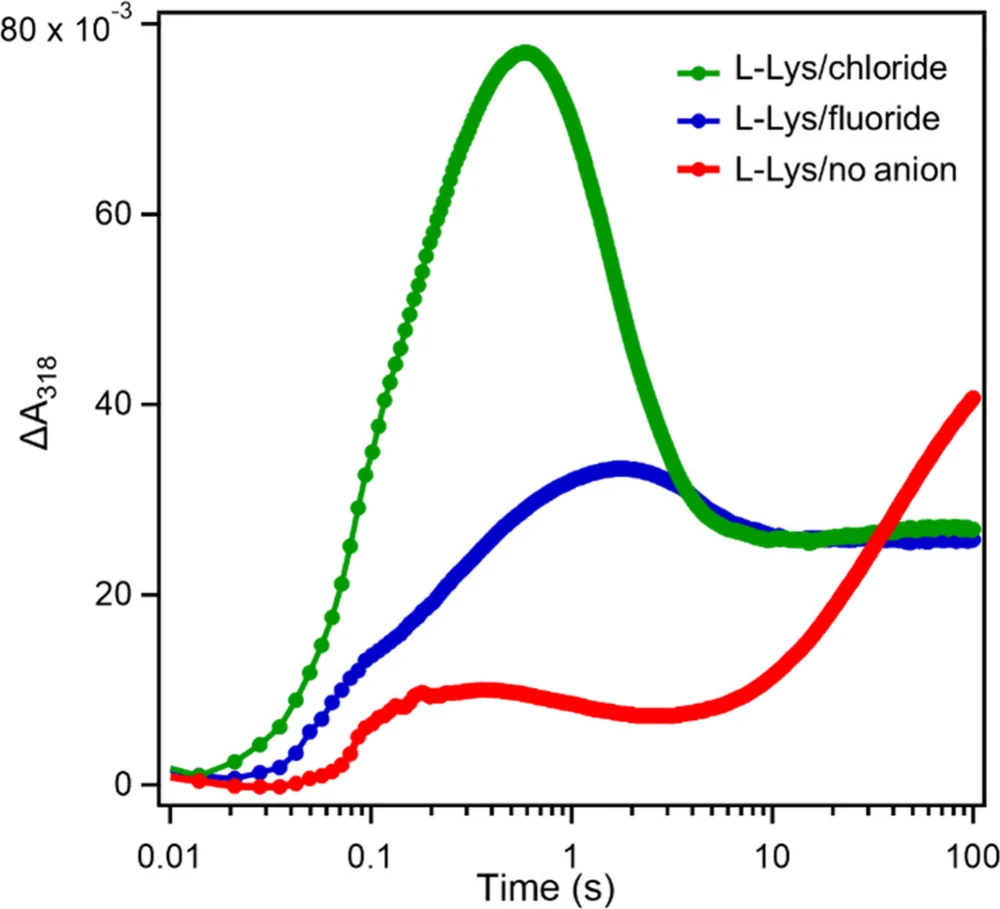
Kinetics of formation and decay of the BesD ferryl intermediate in reactions with with l-Lys and either Cl^–^ (green), F^–^(blue), or no halide (red), as monitored by its absorbance at 318 nm. An anoxic solution containing 0.55 mM BesD, 0.4 mM Fe^II^, 5 mM 2OG, 10 mM l-Lys, and 200 mM NaCl or CsF was mixed at 5 °C with an equal volume of air-saturated buffer (∼0.36 mM [O_2_]).

### Evidence for Fluoroferryl Intermediate Accumulation by Freeze-Quench ^57^Fe Mössbauer Spectroscopy

To verify the oxidation state of the 318 nm-absorbing species in the reaction with fluoride, we further characterized it by freeze-quench ^57^Fe Mössbauer spectroscopy. Previous studies have shown that the presence of deuterium at the targeted position of a substrate leads to increased accumulation of the ferryl intermediate.^90,96,101^ Therefore, we hypothesized that using 4,4,5,5-*d*_4_-l-Lys would similarly result in greater fluoroferryl intermediate accumulation in this case. The 4.2-K/0-mT Mössbauer spectrum of a sample prepared by mixing an O_2_-saturated buffer solution with an equal volume of the BesD•^57^Fe­(II)•2OG•*d*_4_-l-Lys•F^–^ complex and freeze-quenching at a reaction time of 7 s (near the Δ*A*_318_ maximum) exhibits a new quadrupole doublet with parameters δ = 0.24 mm/s and |ΔE_Q_| = 0.36 mm/s that accounts for ∼ 40% of the total spectral intensity (Figure 5). These parameters are typical of a high-spin nonheme ferryl complex, and we therefore attribute the spectrum to the BesD•Fe^IV^(O)•succinate•F^–^•*d*_4_-l-Lys (fluoroferryl) complex.^100^ The parameters are similar—but not identical—to the δ = 0.27 mm/s and |ΔE_Q_| = 0.73 mm/s previously reported for the *cis*-chloroferryl complex in BesD (BesD•Fe^IV^(O)•succinate•Cl^–^•*d*_4_-l-Lys).^90^ The fluoro- and chloroferryl complexes also exhibit distinct kinetic behavior: the former complex continues to accumulate between 7 and 100 s, whereas the latter decays within 2 s.^90^ However, the fluoroferryl complex does eventually decay (within 1000 s), leading to redevelopment of a quadrupole doublet with the same parameters (δ = 1.12 mm/s and |ΔE_Q_| = 2.28 mm/s) as the initial BesD•^57^Fe­(II)•2OG•*d*_4_-l-Lys•F^–^ reactant complex. The Mössbauer data thus establish the formation of a ferryl species from the fluoride-bound quinary complex and its subsequent decay back to the Fe­(II) reactant state. The reformation of an Fe­(II) species rather than decay to an Fe­(III) complex suggests that the intermediate is performing a productive oxidation.

**Figure 5:**
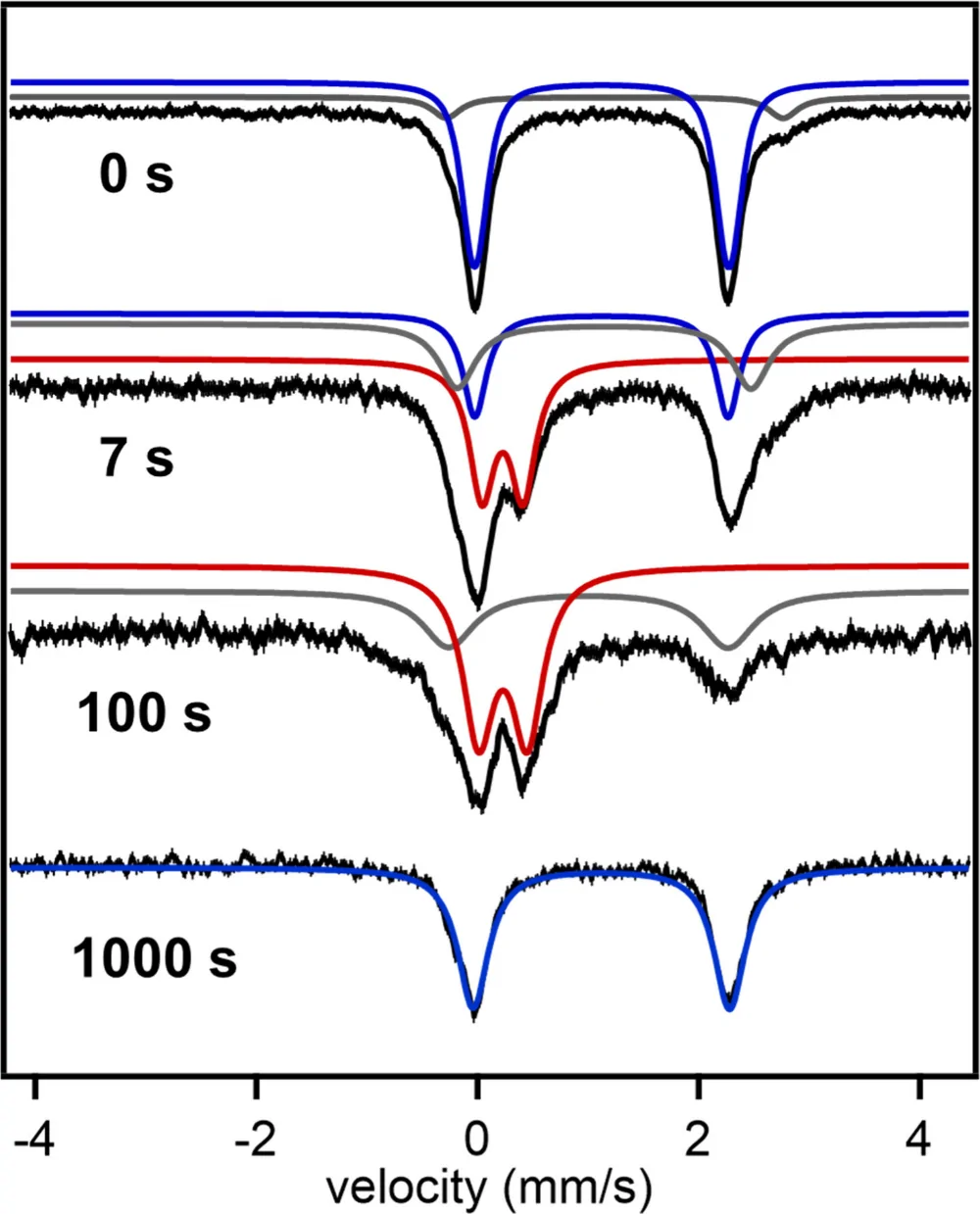
Mössbauer spectra (4.2 K/0 mT) of samples prepared either by freezing an anoxic solution containing 1.8 mM BesD, 1.5 mM ^57^Fe^II^, 16 mM 2OG, 30 mM *d*_4_-l-Lys, and 200 mM NaF (upper spectrum, vertical bars) or by mixing this solution at 5 °C with an equal volume of O_2_-saturated buffer (giving ∼0.9 mM [O_2_] after mixing) and freezing at the reaction times indicated in the figure (middle and lower spectra, vertical bars). Solid lines are simulations of the spectra of Fe^II^ (green) and Fe^IV^ (red) components. The isomer shift and quadrupole splitting parameters of the Fe^II^ and Fe^IV^ species are δ_Fe(II)_ = 1.13 mm/s, |ΔE_Q,Fe(II)_| = 2.28 mm/s, and δ_Fe(IV)_ = 0.24 mm/s, |ΔE_Q,Fe(IV)_| = 0.36 mm/s.

### Evidence for Fluoroferryl Intermediate Accumulation by EPR Detection of the Fe­(III) Product of Its Chemically Induced Decay

Although the freeze-quench ^57^Fe Mössbauer data establish formation of a ferryl complex in the presence of fluoride, they do not provide direct evidence of fluoride coordination to the Fe­(IV) ion. To verify the coordination of fluoride in the ferryl state, we quenched the intermediate by treating it with excess sodium azide, known from prior studies to reduce high-valent iron complexes and generate the azidyl radical.^102^ We hypothesized that excess azide would reduce the EPR-silent *cis*-Fe^IV^(O)­(F) species to an EPR-active high-spin *cis*-Fe^III^(O)­(F) species, thereby enabling direct detection of fluoride through its ^19^F superhyperfine coupling. The transformation was accompanied by distinct color changes: the light purple reactant complex turned pale yellow upon reaction with O_2_, and subsequent addition of azide produced a dark orange solution, consistent with further conversion of the high-valent intermediate. The X-band EPR spectrum of the resulting sample at 12 K has features characteristic of high-spin (S = 5/2) Fe­(III), with *g*-values at 9.22, 4.20, and 1.97 (Figure 6). Rhombogram analysis implies contributions from two spin manifolds: (1) an *M*_S_ = 1/2 ground state, giving rise to both the signal at *g*_eff_ ≈ 9.22, in which the observed splitting can be explained by ^19^F superhyperfine coupling, and a broad feature at *g*_eff_ = 1.98, and (2) an *M*_S_ = 3/2 excited state, responsible for the broad signal at *g*_eff_ = 4.20 (Figure 6A). The temperature dependence of the features supports these assignments: the *g*_eff_= 9.22 signal remains constant from 12 to 8 K, whereas the *g*_eff_ = 4.20 signal decreases in intensity from 12 to 8 K (Figure 6B). Importantly, the *g*_eff_ = 9.22 feature is absent from the spectra of control samples lacking azide (Figure 6A, *gray trace*), demonstrating that the observed high-spin Fe­(III) species arises from azide-mediated reduction of the *cis*-Fe^IV^(O)­(F) intermediate. Other spectral features match those of the autodecay product of the *cis*-Fe^IV^(O)­(F) complex (Figures S17–S19). These results establish that the apparent doublet splitting of the *g*_eff_= 9.22 signal does indeed arise from ^19^F hyperfine coupling to a high-spin Fe­(III) center, thereby providing evidence for the presence of coordinated fluoride in its ferryl precursor. The data thus establish that fluoride not only coordinates to the Fe­(II) cofactor but also remains bound in its higher oxidation states.

**Figure 6:**
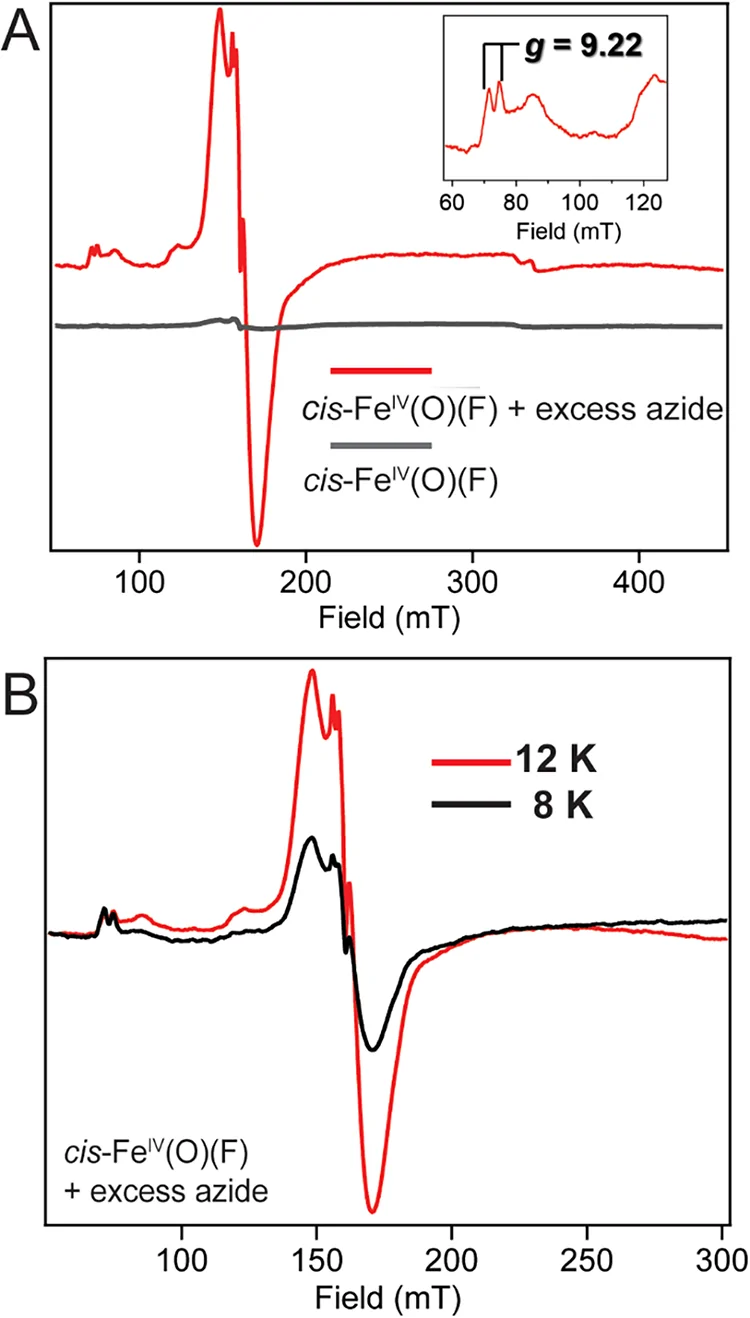
Continuous-wave, X-band (12 K) EPR spectrum of (A) BesD•(Fe^IV^(O)­(F))•l-*d*_4_-Lys•succ (generated by mixing BesD•(Fe^II^(F))•l-*d*_4_-Lys•2OG with O_2_ saturated 100 mM HEPES buffer at pH = 7.5, followed by quenching in liquid nitrogen at 8 s) without (gray trace) and with (red trace) 50 equiv of NaN_3_. Inset shows the g_eff_ = 9.22 region. (B) Overlay of the BesD•(Fe^IV^(O)­(F))•l-*d*_4_-Lys•succ + NaN_3_ (50 equiv) at different temperatures.

### Calculation of Mössbauer Parameters of the Reactant and Ferryl Complexes with Chloride or Fluoride Coordinated

To investigate the structure of the BesD•Fe­(II)•2OG•l-Lys•X^–^ (X = Cl, Br, F) complex, we performed molecular dynamics (MD) simulations along with quantum mechanics/molecular mechanics (QM/MM) calculations (see SI for computational details). A representative snapshot from the MD simulations was first selected, after which the coordinated Cl^–^ ligand was replaced with Br^–^ or F^–^. The resulting structures (Figure 7a–c) were then optimized at the QM/MM level, and the Mössbauer parameters for each species were computed using density functional theory (DFT) methods (further details in the SI). As shown in Table 1, we calculated the isomer shift for the Cl^–^-bound species to be 1.06 mm/s and the absolute value of its quadrupole splitting to be 2.94 mm/s. For the Br^–^ complex, we calculated δ = 1.06 mm/s and |Δ*E*_Q_| = 2.74 mm/s. These computed values are within the expected error range (∼0.06 mm/s for isomer shift and 0.3–0.5 mm/s for quadrupole splitting) of the experimental Mössbauer parameters.^103–105^ For the corresponding complex obtained by replacing chloride with fluoride in the same site (Figure 7c), our calculations predict no change in isomer shift (δ = 1.06 mm/s) but a diminution in quadrupole splitting (|Δ*E*_Q_| = 2.66 mm/s). The prediction is opposite to the experimentally observed increase in δ for the fluoride complex. This discrepancy suggests that the F^–^-coordinated complex may not be well-represented by simple replacement of the Cl^–^ in the native chloroferryl intermediate by F^–^.

**Figure 7:**
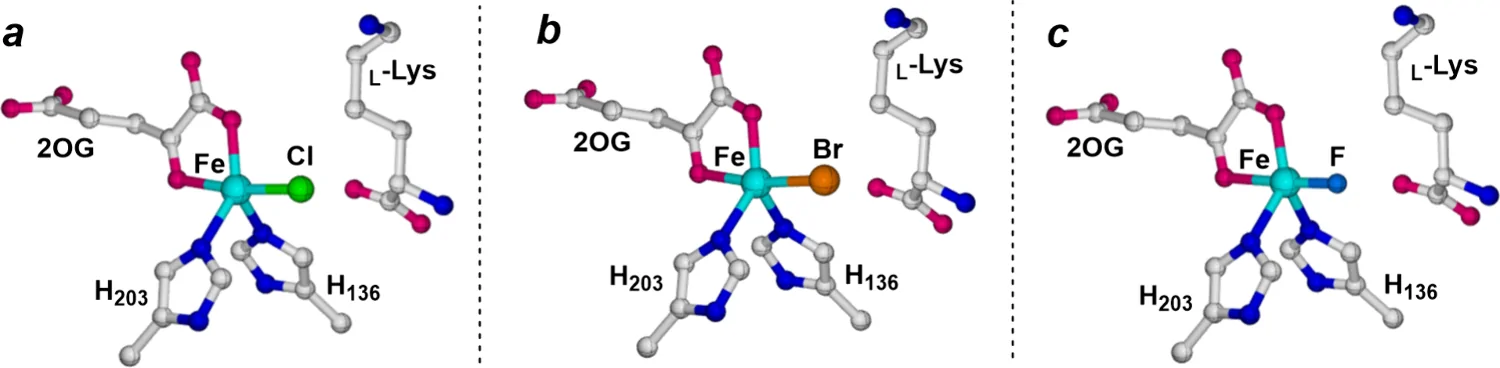
Computed Mössbauer parameters for BesD•Fe^II^(X)•2OG•_L_-Lys (X = Cl, Br, F) complexes. The corresponding geometries and spin populations can be found in the SI.

Therefore, we carried out QM/MM calculations to examine possible alternative structures of the *cis*-chloroferryl and *cis*-fluoroferryl complexes (Table 3). For the chloroferryl complex with the oxo ligand positioned “in-line,″ (Figure S35, *left*) opposite H203 (Figure 8a), the computed Mössbauer parameters are δ = 0.21 mm/s and |Δ*E*_Q_| = 1.00 mm/s, whereas, for the complex with the oxo ligand positioned “off-line,″ (Figure S35, **right**) opposite H136 (Figure 8c), the parameters are δ = 0.22 mm/s and |Δ*E*_Q_| = 1.34 mm/s. With fluoride coordinated, the complex with in-line oxo (Figure 8b) gives δ = 0.17 mm/s and |Δ*E*_Q_| = 0.87 mm/s, whereas the complex with off-line oxo (Figure 8d) gives δ = 0.18 mm/s and |Δ*E*_Q_| = 1.37 mm/s. Thus, regardless of the oxo location, the fluoroferryl complex is predicted to have a 0.04 mm/s smaller isomer shift parameter than its chloroferryl counterpart, in agreement with the 0.03 mm/s difference seen experimentally.

**Figure 8:**
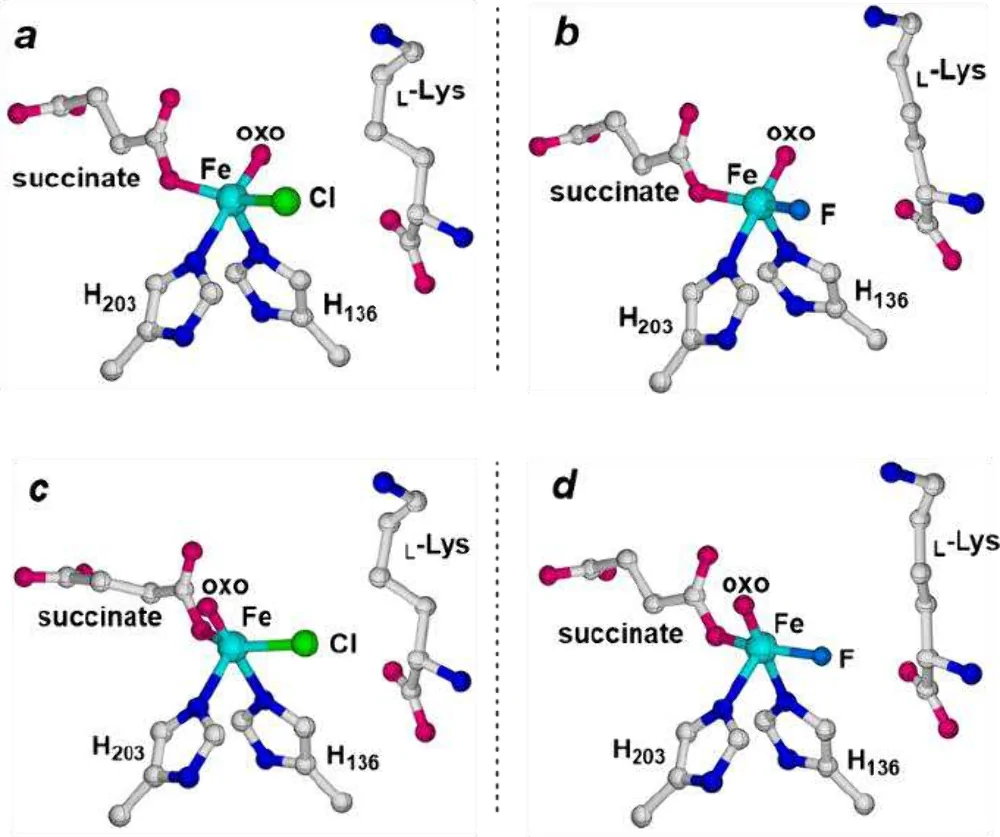
Calculated Mössbauer parameters for ferryl complexes based on QM/MM optimized structures. The corresponding geometries and spin populations can be found in the SI.

**Table 3:** Summary of *cis*-Fe^IV^(O)­(halide) Experimental and Calculated Mössbauer Parameters

	experimental		calculated (monodentate succinate)		calculated (bidentate succinate)	
species	δ (mm/s)	|ΔE_Q_| (mm/s)	δ (mm/s)	|ΔE_Q_| (mm/s)	δ (mm/s)	|ΔE_Q_| (mm/s)
BesD•Fe^IV^(O)•succinate•**Cl** ^**‑**^•l-lys	0.27	0.73	0.21^a^ (0.22^b^)	1.00^a^ (1.37^b^)	0.30^a^ (0.30^b^)	0.84^a^ (1.08^b^)
BesD•Fe^IV^(O)•succinate•**F** ^**‑**^•l-lys	0.24	0.36	0.17^a^ (0.18^b^)	0.87^a^ (1.34^b^)	0.28^a^ (0.24^b^)	0.80^a^ (0.74^b^)

a = in-line orientation of ferryl.

b = off-line orientation of ferryl.

Because a recent computational study invoked bidentate succinate coordination in the ferryl state,^81^ we also assessed the impact of this “carboxylate shift”^106^ on the overall geometric structures and Mössbauer parameters of the *cis*-fluoroferryl and *cis*-chloroferryl complexes, after structural optimization by QM/MM. Whereas the shift to the bidentate mode is predicted to diminish |Δ*E*_Q_| and increase δ for both the in-line and the off-line complexes (Figure S28), thus bringing the calculated values into even better agreement with the experimentally measured values, the predicted impact of replacing chloride with fluoride—diminished values of both |Δ*E*_Q_| and δ—is unchanged in the six-coordinate geometries (see SI for details).

### Characterization of the Vanadyl Complexes by EPR Spectroscopy

In past work, we used the stable vanadyl ion [(V^IV^O)^2+^] to mimic the reactive ferryl intermediate in Fe/2OG enzymes. This diatomic species has an electron-spin ground state of *S* = 1/2, and, when bound in the active site of an Fe/2OG enzyme, exhibits a *g* ≈ 2 EPR signal with strong, anisotropic ^51^V hyperfine coupling.^107^ Previous studies showed that the BesD•VO•succinate complex exhibits heterotropic cooperativity, in which l-Lys and chloride bind synergistically.^90^ We observed the same trend with fluoride (Figure 9A). Differences in the spectra of the BesD•VO•succinate•l-Lys, BesD•VO•succinate•Cl^–^•l-Lys, and BesD•VO•succinate•F^–^•l-Lys complexes are most evident in the low-field (Figure 9B) and high-field (not shown) regions. For the fluoride complex, the spectrum displays an additional splitting pattern that can be attributed to superhyperfine coupling to the ^19^F nucleus. Notably, we detected this splitting only in the presence of both fluoride and l-Lys, consistent with the known heterotropic cooperativity. The observation that fluoride can stably coordinate in the vanadyl complex, which multiple lines of structural,^107–109^ spectroscopic^90,101^ and biochemical^107^ evidence suggest to be an accurate ferryl mimic, is additional evidence that fluoride can also coordinate in the catalytically relevant ferryl state.

**Figure 9:**
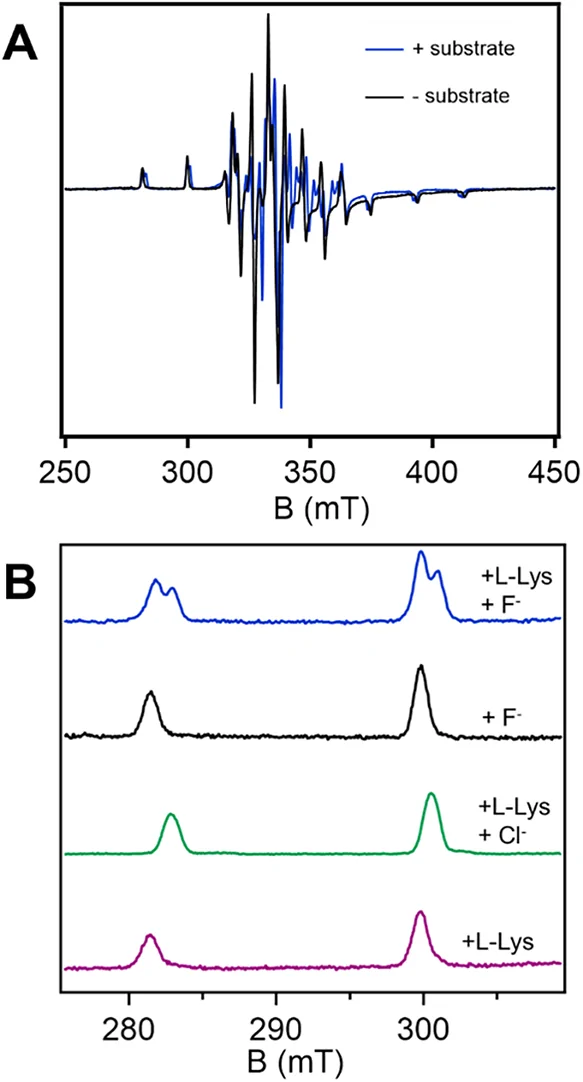
EPR spectra of the BesD•vanadyl•succinate complex in the presence and absence of substrates. (A) Spectra showing the effect of l-Lys binding. (B) Low-field regions of the spectrum of the BesD•vanadyl•succinate complex recorded without substrate or in the presence of l-Lys and/or NaCl or CsF.

### Apparent Substrate Deuterium Kinetic Isotope Effect on Decay of the *cis*-Fluoroferryl Intermediate Establishes Its HAT Competency

Prior studies have shown that the presence of deuterium in the scissile C–H bond of the prime substrate can delay decay of the ferryl complex by as much as 60-fold.^88,96^ In the reaction of the BesD•Fe­(II)•2OG•F^–^•*d*_4_-l-Lys complex with limiting O_2_, the absorbance at 318 nm develops to a greater extent and decays less rapidly (*k*_decay,H_ = 0.2 s^–1^ and *k*_decay,D_ = 0.05 s^–1^) at 5 °C, implying an *observed* deuterium kinetic isotope effect (^2^H-KIE) of ∼ 4 (Figure 10, top). This effect strongly supports the conclusion that a ferryl intermediate accumulates, and, at least for the protium-bearing substrate, abstracts H• from l-Lys in the majority of events. The unexpectedly small value of the observed D-KIE may be explained by the presence of an unproductive ferryl-decay pathway that does not require deuterium transfer and is unimpeded by the isotopic substitution, a mechanism of intrinsic D-KIE masking that was previously shown to occur in BesD.^90^ The unproductive (uncoupled) decay of the ferryl complex in an Fe/2OG enzyme often generates Fe­(III) species, which have absorption features that overlap with those of the ferryl intermediate.^90^ The result is that the absorbance at 318 nm never returns to its baseline value characteristic of the reactant complex, as seen in the stopped-flow traces of the BesD reaction in the presence of fluoride (Figure 10, top). LCMS analysis of the BesD reactions using the natural-isotopic-abundance l-Lys and the 4,4,5,5-*d*_4_-l-Lys isotopologue showed that the presence of deuterium does indeed diminish the yield of the major product. In the 4,4,5,5-*d*_4_-l-Lys reaction, this product gives a peak at *m*/*z* = 166.1, which coelutes with, but has a + 3 *m*/*z* shift relative to, the corresponding 4-OH-l-Lys product from the substrate of natural isotopic abundance. This +3 shift confirms that installation of the OH group proceeds with loss of one deuterium atom, yielding *d*_3_-4-OH-l-Lys. The yield of the *d*_3_-4-OH-l-Lys product is ∼ 4 times less than the yield of the 4-OH-Lys product at *m*/*z* = 163.1 from the natural abundance substrate (Figure 10, bottom). By contrast, the yields of the succinate coproduct are not substantially different in the two reactions. This dichotomy implies that unproductive ferryl-decay pathways dominate for the case of 4,4,5,5-*d*_4_-l-Lys. The relative yields of the hydroxylated products in a series of replicates under varying conditions (Figure S33) gave an effective D-KIE on substrate hydroxylation of 4.7, similar to the value estimated from the stopped-flow absorption traces. The intrinsic D-KIE on the HAT step must be considerably larger than this, because deuterium transfer to the ferryl complex contributes only ∼ 20% of the total decay flux, as shown by the diminished 4-OH-Lys yield from the 4,4,5,5-*d*_4_-l-Lys isotopologue.

**Figure 10:**
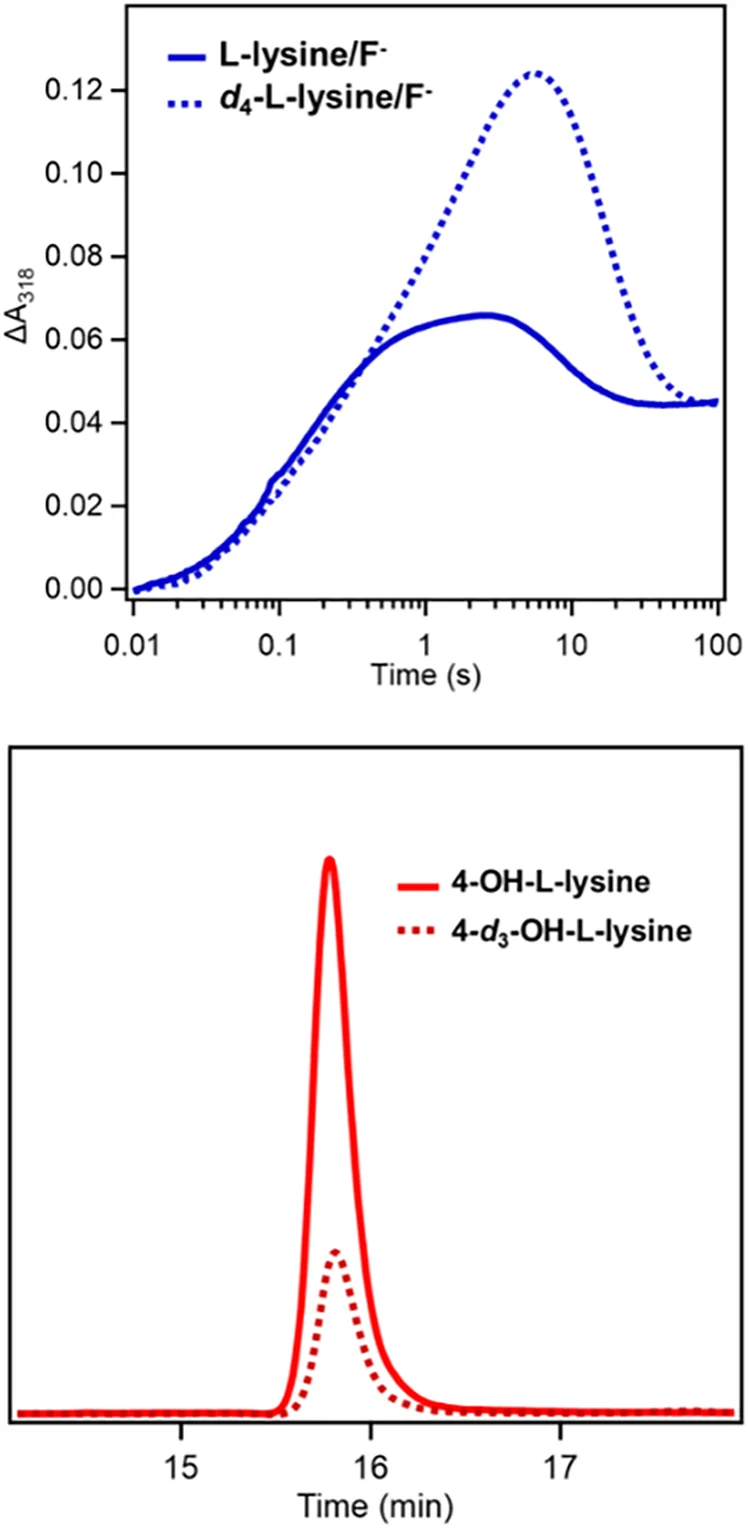
(top) Kinetics of formation and decay of the BesD haloferryl intermediate in reactions of BesD•Fe­(II)•2OG•F^–^ with l-Lys (blue solid line) and *d*_4_-l-Lys (blue dotted line) as monitored by its absorbance at 318 nm. An anoxic solution containing 0.55 mM BesD, 0.4 mM Fe^II^, 5 mM 2OG, 10 mM l-Lys/*d*_4_-l-Lys, and 200 mM of CsF was mixed at 5 °C with an equal volume of air-saturated buffer (∼0.36 mM [O_2_]); (bottom) LCMS characterization (single-ion chromatogram) of the hydroxylysine products formed by adding air saturated buffer (100 mM HEPES, pH 7.5) to BesD•Fe­(II)•2OG•F^–^ with l-Lys (4-OH-l-Lysine, *m*/*z* = 163.1, red solid trace) or *d*_4_-l-Lys (4-OH-*d*_3_-l-Lys, *m*/*z* = 166.1, red dotted trace).

### Computed Radical-Transfer Barriers

In the QM/MM calculations for the final radical-coupling step, we included the Asn218–succinate hydrogen bonding network in the QM region, as it has been suggested to maintain the coordinative unsaturation (i.e., overall penta-coordination) of the iron cofactor.^72^ As prior computational studies on the chloride complexes of BesD pointed to the importance of axial-to-equatorial isomerizations in controlling reaction selectivity,^72,110^ we also investigated different F–Fe­(III)–OH isomers. The calculations suggest that, for isomer **IM1** (Figure 11), in which the OH group is positioned opposite H203, •OH rebound has a lower activation barrier (14.3 kcal/mol) than F• coupling (37.6 kcal/mol). QM/MM scans along different reaction coordinates predicted that isomer **IM2** (Figure S34), in which the OH group is positioned opposite H136, could undergo further isomerization to form **IM3**, in which the OH group is positioned opposite succinate and the fluorine opposite His203. Our calculations suggest that **IM3** should be 2 kcal/mol lower in energy than **IM2**. Starting from **IM3**, the reaction barrier for F• coupling (22.8 kcal/mol) becomes less than that for •OH rebound (29.0 kcal/mol). These calculations hold out hope that fluorination by an Fe/2OG halogenase might indeed be possible by control of intermediate geometry.

**Figure 11:**
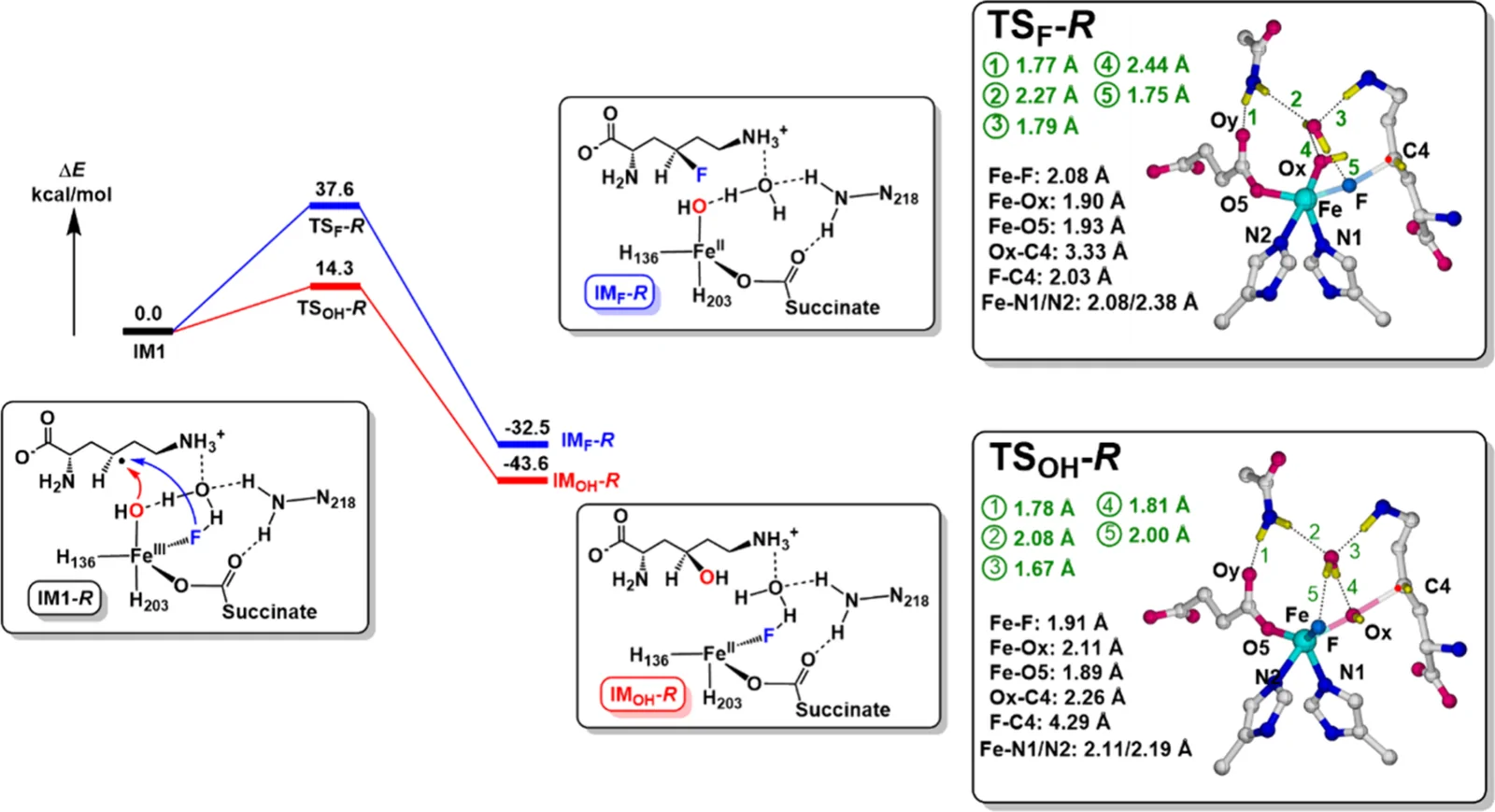
Potential energy profile for the rebound step with different F–Fe­(III)–OH isomeric complexes. Computational details can be found in the SI.

It has been proposed that, in the complex of BesD with chloride bound, conformational isomerization to an **IM3**-like structure drives chlorination selectivity.^72^ In the BesD–fluoride system, isomerization of the F–Fe­(III)–OH species to **IM3** lowers the activation barrier (Δ*E* ^*‡*^) for F• coupling relative to that for •OH rebound, making F• coupling the kinetically preferred channel from that intermediate. However, •OH rebound from **IM1** remains faster overall, with a lower barrier (Δ*E* ^*‡*^ = 14.3 kcal·mol^–1^) than •F transfer from **IM3** (Δ*E* ^*‡*^ = 22.8 kcal·mol^–1^). Thus, product distribution should be governed by kinetic control, and the hydroxylation pathway should dominate because its rate-limiting step has the lower Δ*E* ^*‡*^, consistent with experiment. Nevertheless, these results suggest that controlling ferryl complex isomerization after HAT to stabilize the F–Fe­(III)–OH complex in an **IM3**-like configuration could provide a competitive pathway for fluorine coupling. Here, it should be noted that the ferryl complex can abstract either of the two hydrogens of l-Lys to form F–Fe­(III)–OH intermediates in the *R* or *S* configuration (Figure S30). The computed barriers differ by 3.1 kcal/mol, indicating a stereoselectivity in the HAT step for the *pro-R* hydrogen (Figure S30).

To assess the impact of the boundary selected for the QM region on the predicted reaction barriers, we repeated QM/MM calculations with a smaller QM region lacking the hydrogen-bonding interactions. These calculations indicate that the •OH-rebound pathway still has a lower activation barrier (Δ*E* ^*‡*^) than the competing •F-coupling pathway, rationalizing the observation that hydroxylation is favored in the fluoride-bound BesD system. (see SI for full details).

### Screening of l-Lys Analogues for Different Chemoselectivity

Because prior mechanistic studies on the halogenase SyrB2 showed that the nature of the substrate can strongly influence the outcome of the reaction, owing to the different dispositions of substrate C–H bonds to the cofactor, we examined product yields with analogues of the native BesD prime substrate, l-Lys.^88^ We tested d-lysine, l-homolysine, d-homolysine, l-β-homolysine, l-ornithine and d-ornithine for transformation by BesD. It was previously shown that d-lysine and l-ornithine can also bind in the active site of BesD, albeit with higher dissociation constants than for the native l-Lys substrate.^90^ However, use of these substrates yielded only hydroxylated products (Δ*m*/*z* = +16) for all non-native substrates in the presence of fluoride as the exogenous-anion substrate (Figure S22, left). It thus appears that the suppression of fluorination is consistent across the range of l-Lys analogs. The measured ratios of hydroxylated products to succinate (1:1 in the ideal case) indicate that both native and non-native substrates induce ferryl formation to a comparable degree, resulting in similar levels of succinate production (Figure S22, right). The outcome of ferryl decay varies with the substrate, leading to varying amounts of hydroxylated products. Additionally, the reactions with the non-native substrates also incorporate ^18^O from ^18^O_2_ (50 – 60%) into the hydroxylated products, establishing an •OH-rebound mechanism for hydroxylation of the analogues.

### Site-Directed Mutagenesis Toward the Aim of Eliciting Fluorination

Previous studies suggested that residues in the second sphere can influence the fate of the X–Fe­(IV)=O and X–Fe­(III)–OH intermediates, as the H133A and N218A substitutions in BesD redirected the reaction from halogenation to hydroxylation.^39^ Motivated by these findings, we generated a library of BesD variants via site-directed mutagenesis (see Supporting Information) and evaluated their reactivities with fluoride as the anion. None of the variants produced detectable fluorinated products (Figure S23), suggesting that the barrier to productive C–H fluorination may not readily be overcome by single substitutions. Despite the absence of fluorination, several variants showed altered hydroxylation yields, indicating that even relatively conservative changes (Figure S24) to the second sphere can modulate the partition between rebound and nonrebound pathways. To probe the mechanistic consequences of these substitutions to the hydroxylation step, we performed ^18^O_2_ isotopic-tracer experiments on the wild-type enzyme and the H133A and N218A variants in the presence of fluoride. Incorporation of ^18^O from ^18^O_2_ into the succinate coproduct was >90% for all three variants, as expected for productive O_2_ activation. ^18^O incorporation into the OH-l-Lysine product varied significantly from 55 ± 3% for the wild-type enzyme, to 90 ± 2% for the H133A variant, to a mere 30 ± 2% for the N218A variant. These differences suggest that H133 and N218 impact the substrate-to-cofactor disposition that determines both HAT and •OH-rebound efficiencies.

## Summary and Conclusions

Examination of the reaction of the Fe/2OG l-lysine 4-chlorinase, BesD, with fluoride in place of chloride has shown that anion coordination, O_2_ activation, *cis*-haloferryl intermediate formation, and hydrogen abstraction can all proceed with the smaller non-native halide ion. Instead of F• coupling to the C4 radical produced by HAT, the oxygen rebound step characteristic of hydroxylases prevails, as shown by the incorporation of an O atom from O_2_ into the dominant alcohol product (Scheme 6). QM/MM calculations suggest that the barrier for F• transfer (Δ*E* ^‡^ = 38 kcal/mol) is substantially higher than for rebound (Δ*E* ^‡^ = 14 kcal/mol), but the additional prediction that the barrier depends significantly on the coordination geometry of the halo-hydroxoiron­(III) complex engenders hope that this selectivity could be reversed by active site re-engineering. The comprehensive mechanistic framework established in this work can potentially inform such efforts.

**Scheme 6:**
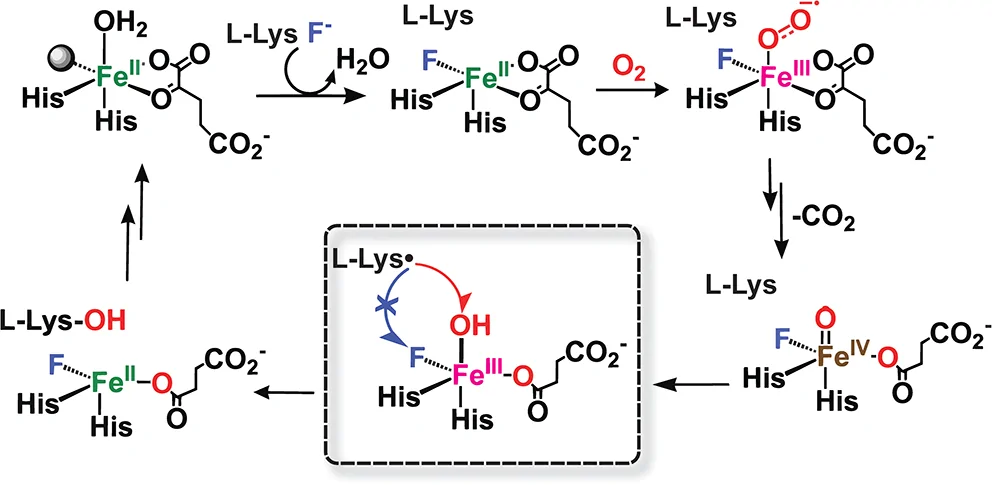
Effect of Fluoride Coordination in Fe/2OG Halogenase

## Supplementary Material



## Notes

Experimental procedures, including details of protein purification, mutation studies, supporting figures (S1–S33) providing additional analysis of SF-Abs, Mössbauer, EPR spectra, DFT calculations, and additional LCMS characterization of BesD reactivity; supporting tables (S1–S6) with fits of the XAS spectra (PDF); and Cartesian coordinates of the optimized geometries (PDF)

## References

[1] Inoue M., Sumii Y., Shibata N. (2020). Contribution of Organofluorine Compounds
to Pharmaceuticals. ACS Omega.

[2] Padamata S. K., Yasinskiy A., Stopic S., Friedrich B. (2022). Fluorination
of two-dimensional graphene: A review. J. Fluorine
Chem..

[3] Fujiwara T., O’Hagan D. (2014). Successful fluorine-containing herbicide agrochemicals. J. Fluorine Chem..

[4] Peng H. (2019). Synthesis
and Application of Fluorine-Containing Polymers with Low Surface Energy. Polym. Rev..

[5] Cahard D., Bizet V. (2014). The influence of fluorine in asymmetric
catalysis. Chem. Soc. Rev..

[6] Gillis E. P., Eastman K. J., Hill M. D., Donnelly D. J., Meanwell N. A. (2015). Applications
of Fluorine in Medicinal Chemistry. J. Med.
Chem..

[7] He J., Li Z., Dhawan G., Zhang W., Sorochinsky A. E., Butler G., Soloshonok V. A., Han J. (2023). Fluorine-containing
drugs approved by the FDA in 2021. Chin. Chem.
Lett..

[8] Halder R., Ritter T. (2021). ^18^F-Fluorination: Challenge and Opportunity
for Organic Chemists. J. Org. Chem..

[9] Chandra G., Singh D. V., Mahato G. K., Patel S. (2023). Fluorine-a small magic
bullet atom in the drug development: perspective to FDA approved and
COVID-19 recommended drugs. Chem. Pap..

[10] Bloom S., Pitts C. R., Miller D. C., Haselton N., Holl M. G., Urheim E., Lectka T. (2012). A Polycomponent Metal-Catalyzed Aliphatic,
Allylic, and Benzylic Fluorination. Angew. Chem.
Int. Ed..

[11] Bloom S., Pitts C. R., Woltornist R., Griswold A., Holl M. G., Lectka T. (2013). Iron­(II)-Catalyzed Benzylic Fluorination. Org. Lett..

[12] Capilato J. N., Pitts C. R., Rowshanpour R., Dudding T., Lectka T. (2020). Site-Selective
Photochemical Fluorination of Ketals: Unanticipated Outcomes in Selectivity
and Stability. J. Org. Chem..

[13] Pitts C. R., Bloom S., Woltornist R., Auvenshine D. J., Ryzhkov L. R., Siegler M. A., Lectka T. (2014). Direct, Catalytic
Monofluorination
of *sp*
^3^ C–H Bonds: A Radical-Based
Mechanism with Ionic Selectivity. J. Am. Chem.
Soc..

[14] Panda C., Anny-Nzekwue O., Doyle L. M., Gericke R., McDonald A. R. (2023). Evidence
for a High-Valent Iron-Fluoride That Mediates Oxidative C­(*sp*
^3^)-H Fluorination. JACS
Au.

[15] Buss J. A., Vasilopoulos A., Golden D. L., Stahl S. S. (2020). Copper-Catalyzed
Functionalization of Benzylic C–H Bonds with N-Fluorobenzenesulfonimide:
Switch from C–N to C–F Bond Formation Promoted by a
Redox Buffer and Bro̷nsted Base. Org.
Lett..

[16] Vasilopoulos A., Golden D. L., Buss J. A., Stahl S. S. (2020). Copper-Catalyzed
C–H Fluorination/Functionalization Sequence Enabling Benzylic
C–H Cross Coupling with Diverse Nucleophiles. Org. Lett..

[17] Szpera R., Moseley D. F. J., Smith L. B., Sterling A. J., Gouverneur V. (2019). The Fluorination
of C–H Bonds: Developments and Perspectives. Angew. Chem. Int. Ed..

[18] Lin A., Huehls C. B., Yang J. (2014). Recent advances
in C–H fluorination. Org. Chem. Front..

[19] Cheng Q., Ritter T. (2019). New Directions in C-H
Fluorination. Trends Chem..

[20] Patel C., André-Joyaux E., Leitch J. A., Martínez de Irujo-Labalde X., Ibba F., Struijs J., Ellwanger M. A., Paton R., Browne D. L., Pupo G., Aldridge S., Hayward M. A., Gouverneur V. (2023). Fluorochemicals from fluorspar via
a phosphate-enabled mechanochemical process that bypasses HF. Science.

[21] Herr C. Q., Hausinger R. P. (2018). Amazing
Diversity in Biochemical Roles of Fe­(II)/2-Oxoglutarate
Oxygenases. Trends Biochem. Sci..

[22] de
Munnik M., Brasnett A., Zhou T., Myers W., Wang Y., Chatterjee K., Tumber A., Marshall S. A., Simon P. S., Aller P. (2026). Structural basis of the promiscuity
of the unusual Fe­(II) and 2-oxoglutarate dependent human aspartate/asparagine-β-hydroxylase. Nat. Commun..

[23] Zhao S., Wu L., Xu Y., Nie Y. (2025). Fe­(ii) and
2-oxoglutarate-dependent
dioxygenases for natural product synthesis: molecular insights into
reaction diversity. Nat. Prod. Rep..

[24] Islam M. S., Leissing T. M., Chowdhury R., Hopkinson R. J., Schofield C. J. (2018). 2-Oxoglutarate-Dependent Oxygenases. Annu. Rev. Biochem..

[25] Jia B., Jia X., Kim K. H., Jeon C. O. (2017). Integrative view of 2-oxoglutarate/Fe­(II)-dependent
oxygenase diversity and functions in bacteria. Biochim. Biophys. Acta- General Subjects.

[26] Wang H., Mori T., Abe I. (2026). Harnessing
Fe­(II)/α-ketoglutarate-dependent
oxygenases for sustainable synthesis. Curr.
Opin. Green Sustainable Chem..

[27] Su Y., Lai W. (2024). Unraveling
the Mechanism of the Oxidative C–C Bond Coupling
Reaction Catalyzed by Deoxypodophyllotoxin Synthase. Inorg. Chem..

[28] Chang W.-c., Yang Z.-J., Tu Y.-H., Chien T.-C. (2019). Reaction Mechanism
of a Nonheme Iron Enzyme Catalyzed Oxidative Cyclization via C–C
Bond Formation. Org. Lett..

[29] Dunbar K. L., Scharf D. H., Litomska A., Hertweck C. (2017). Enzymatic Carbon–Sulfur
Bond Formation in Natural Product Biosynthesis. Chem. Rev..

[30] Blasiak L. C., Vaillancourt F. H., Walsh C. T., Drennan C. L. (2006). Crystal
structure
of the non-haem iron halogenase SyrB2 in syringomycin biosynthesis. Nature.

[31] Bleher K., Comba P., Faltermeier D., Gupta A., Kerscher M., Krieg S., Martin B., Velmurugan G., Yang S. (2022). Non-Heme-Iron-Mediated Selective
Halogenation of Unactivated Carbon–Hydrogen
Bonds. Chem.—Eur. J..

[32] Pan J., Bhardwaj M., Zhang B., Chang W.-c., Schardl C. L., Krebs C., Grossman R. B., Bollinger J. M. (2018). Installation of the Ether Bridge
of Lolines by the
Iron- and 2-Oxoglutarate-Dependent Oxygenase, LolO: Regio- and Stereochemistry
of Sequential Hydroxylation and Oxacyclization Reactions. Biochemistry.

[33] Panth N., Wenger E. S., Krebs C., Bollinger J. M., Grossman R. B. (2022). Synthesis of 6,6-
and 7,7-Difluoro-1-acetamidopyrrolizidines
and Their Oxidation Catalyzed by the Nonheme Fe Oxygenase LolO. ChemBioChem..

[34] Li J., Liao H.-J., Tang Y., Huang J.-L., Cha L., Lin T.-S., Lee J. L., Kurnikov I. V., Kurnikova M. G., Chang W.-c., Chan N. -L., Guo Y. (2020). Epoxidation Catalyzed
by the Nonheme Iron­(II)- and 2-Oxoglutarate-Dependent Oxygenase, AsqJ:
Mechanistic Elucidation of Oxygen Atom Transfer by a Ferryl Intermediate. J. Am. Chem. Soc..

[35] Cha L., Paris J. C., Zanella B., Spletzer M., Yao A., Guo Y., Chang W.-c. (2023). Mechanistic
Studies of Aziridine Formation Catalyzed
by Mononuclear Non-Heme Iron Enzymes. J. Am.
Chem. Soc..

[36] Shen Y., Sun A., Guo Y., Chang W.-c. (2025). Discovery of Noncanonical Iron and
2-Oxoglutarate Dependent Enzymes Involved in C–C and C–N
Bond Formation in Biosynthetic Pathways. ACS
Bio Med. Chem. Au.

[37] Neti S. S., Wang B., Iwig D. F., Onderko E. L., Booker S. J. (2023). Enzymatic
Fluoromethylation Enabled by the S-Adenosylmethionine Analog Te-Adenosyl-L-(fluoromethyl)­homotellurocysteine. ACS Cent. Sci..

[38] Latham J., Brandenburger E., Shepherd S. A., Menon B. R. K., Micklefield J. (2018). Development
of Halogenase Enzymes for Use in Synthesis. Chem. Rev..

[39] Neugebauer M. E., Sumida K. H., Pelton J. G., McMurry J. L., Marchand J. A., Chang M. C. Y. (2019). A family of radical halogenases for
the engineering
of amino-acid-based products. Nat. Chem. Biol..

[40] Mitchell A. J., Zhu Q., Maggiolo A. O., Ananth N. R., Hillwig M. L., Liu X., Boal A. K. (2016). Structural basis for halogenation by iron- and 2-oxo-glutarate-dependent
enzyme WelO5. Nat. Chem. Biol..

[41] Kissman E. N., Neugebauer M. E., Sumida K. H., Swenson C. V., Sambold N. A., Marchand J. A., Millar D. C., Chang M. C. Y. (2023). Biocatalytic
control of site-selectivity and chain length-selectivity in radical
amino acid halogenases. Proc. Natl. Acad. Sci.
U. S. A..

[42] Dai L., Zhang X., Hu Y., Shen J., Zhang Q., Zhang L., Min J., Chen C.-C., Liu Y., Huang J.-W., Guo R. -T. (2022). Structural and Functional Insights
into a Nonheme Iron- and α-Ketoglutarate-Dependent Halogenase
That Catalyzes Chlorination of Nucleotide Substrates. Appl. Environ. Microbiol..

[43] Galonić
Fujimori D., Barr E. W., Matthews M. L., Koch G. M., Yonce J. R., Walsh C. T., Bollinger J. M., Krebs C., Riggs-Gelasco P. J. (2007). Spectroscopic Evidence for a High-Spin
Br-Fe­(IV)-Oxo Intermediate in the α-Ketoglutarate-Dependent
Halogenase CytC3 from Streptomyces. J. Am. Chem.
Soc..

[44] Srnec M., Solomon E. I. (2017). Frontier Molecular Orbital Contributions to Chlorination
versus Hydroxylation Selectivity in the Non-Heme Iron Halogenase SyrB2. J. Am. Chem. Soc..

[45] Groves J. T., Nemo T. E. (1983). Aliphatic hydroxylation
catalyzed by iron porphyrin
complexes. J. Am. Chem. Soc..

[46] Matthews M. L., Krest C. M., Barr E. W., Vaillancourt F. H., Walsh C. T., Green M. T., Krebs C., Bollinger J. M. (2009). Substrate-Triggered
Formation and Remarkable Stability of the C–H Bond-Cleaving
Chloroferryl Intermediate in the Aliphatic Halogenase, SyrB2. Biochemistry.

[47] Kim C. Y., Mitchell A. J., Glinkerman C. M., Li F.-S., Pluskal T., Weng J.-K. (2020). The chloroalkaloid
(−)-acutumine is biosynthesized
via a Fe­(II)- and 2-oxoglutarate-dependent halogenase in Menispermaceae
plants. Nat. Commun..

[48] Planas O., Clémancey M., Latour J.-M., Company A., Costas M. (2014). Structural
modeling of iron halogenases: synthesis and reactivity of halide-iron­(iv)-oxo
compounds. Chem. Commun..

[49] Puri M., Biswas A. N., Fan R., Guo Y., Que L. (2016). Modeling Non-Heme
Iron Halogenases: High-Spin Oxoiron­(IV)–Halide Complexes That
Halogenate C–H Bonds. J. Am. Chem. Soc..

[50] Chatterjee S., Paine T. K. (2016). Hydroxylation versus Halogenation of Aliphatic C–H
Bonds by a Dioxygen-Derived Iron–Oxygen Oxidant: Functional
Mimicking of Iron Halogenases. Angew. Chem.
Int. Ed..

[51] Yadav V., Rodriguez R. J., Siegler M. A., Goldberg D. P. (2020). Determining the
Inherent Selectivity for Carbon Radical Hydroxylation versus Halogenation
with Fe^III^(OH)­(X) Complexes: Relevance to the Rebound Step
in Non-heme Iron Halogenases. J. Am. Chem. Soc..

[52] Yadav V., Siegler M. A., Goldberg D. P. (2021). Temperature-Dependent Reactivity
of a Non-heme Fe^III^(OH)­(SR) Complex: Relevance to Isopenicillin
N Synthase. J. Am. Chem. Soc..

[53] Yadav V., Wen L., Rodriguez R. J., Siegler M. A., Goldberg D. P. (2022). Nonheme Iron­(III)
Azide and Iron­(III) Isothiocyanate Complexes: Radical Rebound Reactivity,
Selectivity, and Catalysis. J. Am. Chem. Soc..

[54] Yadav V., Wen L., Yadav S., Siegler M. A., Goldberg D. P. (2023). Selective Radical
Transfer in a Series of Nonheme Iron­(III) Complexes. Inorg. Chem..

[55] Yadav V., Wen L., Yadav S., Siegler M. A., Goldberg D. P. (2025). Nonheme Mononuclear
and Dinuclear Iron­(II) and Iron­(III) Fluoride Complexes and Their
Fluorine Radical Transfer Reactivity. Inorg.
Chem..

[56] Bower J. K., Cypcar A. D., Henriquez B., Stieber S. C. E., Zhang S. (2020). C­(*sp*
^3^)–H Fluorination with a Copper­(II)/(III)
Redox Couple. J. Am. Chem. Soc..

[57] Hintz H., Bower J., Tang J., LaLama M., Sevov C., Zhang S. (2023). Copper-catalyzed electrochemical C–H fluorination. Chem. Catal..

[58] Panda C., Doyle L. M., Gericke R., McDonald A. R. (2021). Rapid Iron­(III)–Fluoride-Mediated
Hydrogen Atom Transfer. Angew. Chem. Int. Ed..

[59] Rana S., Biswas J. P., Sen A., Clémancey M., Blondin G., Latour J.-M., Rajaraman G., Maiti D. (2018). Selective C–H halogenation over hydroxylation by non-heme
iron­(iv)-oxo. Chem. Sci..

[60] Jana R. D., Sheet D., Chatterjee S., Paine T. K. (2018). Aliphatic C–H
Bond Halogenation by Iron­(II)-α-Keto Acid Complexes and O2:
Functional Mimicking of Nonheme Iron Halogenases. Inorg. Chem..

[61] Jang E. S., McMullin C. L., Käß M., Meyer K., Cundari T. R., Warren T. H. (2014). Copper­(II) Anilides
in *sp*
^3^ C-H Amination. J. Am. Chem. Soc..

[62] Iovan D. A., Betley T. A. (2016). Characterization
of Iron-Imido Species Relevant for
N-Group Transfer Chemistry. J. Am. Chem. Soc..

[63] de
Visser S. P., Latifi R. (2009). Carbon Dioxide: A Waste Product in
the Catalytic Cycle of α-Ketoglutarate Dependent Halogenases
Prevents the Formation of Hydroxylated By-Products. J. Phys. Chem. B.

[64] Gérard E. F., Yadav V., Goldberg D. P., de Visser S. P. (2022). What Drives
Radical Halogenation versus Hydroxylation in Mononuclear Nonheme Iron
Complexes? A Combined Experimental and Computational Study. J. Am. Chem. Soc..

[65] Quesne M. G., de Visser S. P. (2012). Regioselectivity
of substrate hydroxylation versus
halogenation by a nonheme iron­(IV)–oxo complex: possibility
of rearrangement pathways. JBIC J. Biol. Inorg.
Chem..

[66] Timmins A., De Visser S. P. (2018). A Comparative Review on the Catalytic
Mechanism of
Nonheme Iron Hydroxylases and Halogenases. Catalysts.

[67] Timmins A., Fowler N. J., Warwicker J., Straganz G. D., de Visser S. P. (2018). Does Substrate
Positioning Affect the Selectivity and Reactivity in the Hectochlorin
Biosynthesis Halogenase?. Front. Chem..

[68] Timmins A., Quesne M. G., Borowski T., de Visser S. P. (2018). Group Transfer
to an Aliphatic Bond: A Biomimetic Study Inspired by Nonheme Iron
Halogenases. ACS Catal..

[69] Matthews M. L., Neumann C. S., Miles L. A., Grove T. L., Booker S. J., Krebs C., Walsh C. T., Bollinger J. M. (2009). Substrate
positioning controls the partition between halogenation and hydroxylation
in the aliphatic halogenase, SyrB2. Proc. Natl.
Acad. Sci. U. S. A..

[70] Kastner D. W., Nandy A., Mehmood R., Kulik H. J. (2023). Mechanistic Insights
into Substrate Positioning That Distinguish Non-heme Fe­(II)/α-Ketoglutarate-Dependent
Halogenases and Hydroxylases. ACS Catal..

[71] Huang J., Li C., Wang B., Sharon D. A., Wu W., Shaik S. (2016). Selective
Chlorination of Substrates by the Halogenase SyrB2 Is Controlled by
the Protein According to a Combined Quantum Mechanics/Molecular Mechanics
and Molecular Dynamics Study. ACS Catal..

[72] Zhang J., Li Y., Yuan W., Zhang X., Si Y., Wang B. (2024). Conformational
Isomerization of the Fe­(III)–OH Species Enables Selective Halogenation
in Carrier-Protein-Independent Halogenase BesD and Hydroxylase-Evolved
Halogenase. ACS Catal..

[73] Hayashi T., Ligibel M., Sager E., Voss M., Hunziker J., Schroer K., Snajdrova R., Buller R. (2019). Evolved Aliphatic Halogenases
Enable Regiocomplementary C–H Functionalization of a Pharmaceutically
Relevant Compound. Angew. Chem. Int. Ed..

[74] Papadopoulou A., Meyer F., Buller R. M. (2023). Engineering Fe­(II)/α-Ketoglutarate-Dependent
Halogenases and Desaturases. Biochemistry.

[75] Neugebauer M. E., Kissman E. N., Marchand J. A., Pelton J. G., Sambold N. A., Millar D. C., Chang M. C. Y. (2022). Reaction pathway engineering converts
a radical hydroxylase into a halogenase. Nat.
Chem. Biol..

[76] Mitchell A. J., Dunham N. P., Bergman J. A., Wang B., Zhu Q., Chang W.-c., Liu X., Boal A. K. (2017). Structure-Guided
Reprogramming of a Hydroxylase To Halogenate Its Small Molecule Substrate. Biochemistry.

[77] Papadopoulou A., Meierhofer J., Meyer F., Hayashi T., Schneider S., Sager E., Buller R. (2021). Re-Programming and
Optimization of
a L-Proline *cis*-4-Hydroxylase for the *cis*-3-Halogenation of its Native Substrate. ChemCatChem..

[78] Gomez C. A., Mondal D., Du Q., Chan N., Lewis J. C. (2023). Directed
Evolution of an Iron­(II)- and α-Ketoglutarate-Dependent Dioxygenase
for Site-Selective Azidation of Unactivated Aliphatic C–H Bonds. Angew. Chem. Int. Ed..

[79] Kayrouz C. S., Manske J. L., Garçon M., Joyner I. A., Wang Y., Ge Y., Paton A. E., Narayan A. R. H., Hartwig J. F. (2025). Site-, Stereo-,
and Chemoselective Enzymatic Halogenation of Terpenoids by a Substrate
Masquerade. J. Am. Chem. Soc..

[80] Hillwig M. L., Zhu Q., Ittiamornkul K., Liu X. (2016). Discovery of a Promiscuous Non-Heme
Iron Halogenase in Ambiguine Alkaloid Biogenesis: Implication for
an Evolvable Enzyme Family for Late-Stage Halogenation of Aliphatic
Carbons in Small Molecules. Angew. Chem. Int.
Ed..

[81] Vennelakanti V., Li G. L., Kulik H. J. (2023). Why Nonheme Iron Halogenases Do Not
Fluorinate C–H Bonds: A Computational Investigation. Inorg. Chem..

[82] Zhao Q., Chen Z., Soler J., Chen X., Rui J., Ji N. T., Yu Q. E., Yang Y., Garcia-Borràs M., Huang X. (2024). Engineering
non-haem iron enzymes for enantioselective C­(*sp*
^3^)–F bond formation via radical fluorine
transfer. Nat. Synth..

[83] Zhao L.-P., Mai B. K., Cheng L., Gao F., Zhao Y., Guo R., Wu H., Zhang Y., Liu P., Yang Y. (2024). Biocatalytic
enantioselective C­(*sp*
^3^)–H fluorination
enabled by directed evolution of non-haem iron enzymes. Nat. Synth..

[84] Marchand J. A., Neugebauer M. E., Ing M. C., Lin C. I., Pelton J. G., Chang M. C. Y. (2019). Discovery of a pathway for terminal-alkyne
amino acid
biosynthesis. Nature.

[85] Smithwick E. R., Wilson R. H., Chatterjee S., Pu Y., Dalluge J. J., Damodaran A. R., Bhagi-Damodaran A. (2023). Electrostatically
Regulated Active
Site Assembly Governs Reactivity in Nonheme Iron Halogenases. ACS Catal..

[86] Chan N. H., Gomez C. A., Vennelakanti V., Du Q., Kulik H. J., Lewis J. C. (2022). Non-Native Anionic Ligand Binding
and Reactivity in
Engineered Variants of the Fe­(II)- and α-Ketoglutarate-Dependent
Oxygenase. SadA. Inorg. Chem..

[87] Zhan C.-G., Dixon D. A. (2004). Hydration of the Fluoride Anion: Structures and Absolute
Hydration Free Energy from First-Principles Electronic Structure Calculations. J. Phys. Chem. A.

[88] Matthews M. L., Neumann C. S., Miles L. A., Grove T. L., Booker S. J., Krebs C., Walsh C. T., Bollinger J. M. (2009). Substrate
positioning controls the partition between halogenation and hydroxylation
in the aliphatic halogenase, SyrB2. Proc. Natl.
Acad. Sci. U. S. A..

[89] Pan J., Wenger E. S., Matthews M. L., Pollock C. J., Bhardwaj M., Kim A. J., Allen B. D., Grossman R. B., Krebs C., Bollinger J. M. (2019). Evidence for Modulation of Oxygen
Rebound Rate in Control of Outcome by Iron­(II)- and 2-Oxoglutarate-Dependent
Oxygenases. J. Am. Chem. Soc..

[90] Slater J. W., Lin C.-Y., Neugebauer M. E., McBride M. J., Sil D., Nair M. A., Katch B. J., Boal A. K., Chang M. C. Y., Silakov A., Krebs C., Bollinger J. M. (2023). Synergistic Binding of the Halide and Cationic Prime
Substrate of l-Lysine 4-Chlorinase, BesD, in Both Ferrous and Ferryl
States. Biochemistry.

[91] Yu D., Xu L., Fu K., Liu X., Wang S., Wu M., Lu W., Lv C., Luo J. (2024). Electronic structure modulation of
iron sites with fluorine coordination enables ultra-effective H_2_O_2_ activation. Nat. Commun..

[92] Cutsail G. E., Blaesi E. J., Pollock C. J., Bollinger J. M., Krebs C., DeBeer S. (2020). High-resolution iron
X-ray absorption
spectroscopic and computational studies of non-heme diiron peroxo
intermediates. J. Inorg. Biochem..

[93] Castillo R. G., Banerjee R., Allpress C. J., Rohde G. T., Bill E., Que L., Lipscomb J. D., DeBeer S. (2017). High-Energy-Resolution Fluorescence-Detected
X-ray Absorption of the Q Intermediate of Soluble Methane Monooxygenase. J. Am. Chem. Soc..

[94] Martinie R. J., Livada J., Chang W.-c., Green M. T., Krebs C., Bollinger J. M., Silakov A. (2015). Experimental Correlation of Substrate
Position with Reaction Outcome in the Aliphatic Halogenase, SyrB2. J. Am. Chem. Soc..

[95] Zhang Y., Oldfield E. (2004). On the Mössbauer
Spectra of Isopenicillin N
Synthase and a Model {FeNO}^7^ (*S* = 3/2)
System. J. Am. Chem. Soc..

[96] Price J. C., Barr E. W., Tirupati B., Bollinger J. M., Krebs C. (2003). The First Direct Characterization
of a High-Valent Iron Intermediate
in the Reaction of an α-Ketoglutarate-Dependent Dioxygenase:
A High-Spin Fe­(IV) Complex in Taurine/α-Ketoglutarate Dioxygenase
(TauD) from Escherichia coli. Biochemistry.

[97] Sanakis Y., Petasis D., Petrouleas V., Hendrich M. (1999). Simultaneous Binding
of Fluoride and NO to the Nonheme Iron of Photosystem II: Quantitative
EPR Evidence for a Weak Exchange Interaction between the Semiquinone
Q_A_
^–^ and the Iron-Nitrosyl Complex. J. Am. Chem. Soc..

[98] Matthews M. L., Chang W.-c., Layne A. P., Miles L. A., Krebs C., Bollinger J. M. (2014). Direct
nitration and azidation of aliphatic carbons
by an iron-dependent halogenase. Nat. Chem.
Biol..

[99] Pratter S. M., Light K. M., Solomon E. I., Straganz G. D. (2014). The Role of Chloride
in the Mechanism of O_2_ Activation at the Mononuclear Nonheme
Fe­(II) Center of the Halogenase HctB. J. Am.
Chem. Soc..

[100] Krebs C., Galonić Fujimori D., Walsh C. T., Bollinger J. M. (2007). Non-Heme
Fe­(IV)–Oxo Intermediates. Acc. Chem.
Res..

[101] Wenger E. S., Martinie R. J., Ushimaru R., Pollock C. J., Sil D., Li A., Hoang N., Palowitch G. M., Graham B. P., Schaperdoth I., Burke E. J., Maggiolo A. O., Chang W. -c., Allen B. D., Krebs C., Silakov A., Boal A. K., Bollinger J. M. (2024). Optimized Substrate
Positioning Enables Switches in the C–H Cleavage Site and Reaction
Outcome in the Hydroxylation–Epoxidation Sequence Catalyzed
by Hyoscyamine 6β-Hydroxylase. J. Am.
Chem. Soc..

[102] Kalyanaraman B., Janzen E. G., Mason R. P. (1985). Spin Trapping of
the Azidyl Radical in Azide/Catalase/H_2_O_2_ and
Various Azide/Peroxidase/H_2_O_2_ Peroxidizing Systems. J. Biol. Chem..

[103] Römelt M., Ye S., Neese F. (2009). Calibration
of Modern
Density Functional Theory Methods for the Prediction of ^57^Fe Mössbauer Isomer Shifts: Meta-GGA and Double-Hybrid Functionals. Inorg. Chem..

[104] Wang C., Chen H. (2017). Convergent Theoretical Prediction
of Reactive Oxidant Structures in Diiron Arylamine Oxygenases AurF
and CmlI: Peroxo or Hydroperoxo?. J. Am. Chem.
Soc..

[105] Santra G., Neese F., Pantazis D. A. (2024). Extensive reference
set and refined computational protocol for calculations of ^57^Fe Mössbauer parameters. Phys. Chem.
Chem. Phys..

[106] Rardin R. L., Tolman W. (1991). MONODENTATE CARBOXYLATE COMPLEXES
AND THE CARBOXYLATE SHIFT: IMPLICATIONS FOR POLYMETALLOPROTEIN STRUCTURE
AND FUNCTION. New J. Chem..

[107] Martinie R. J., Pollock C. J., Matthews M. L., Bollinger J. M., Krebs C., Silakov A. (2017). Vanadyl as a Stable Structural Mimic
of Reactive Ferryl Intermediates in Mononuclear Nonheme-Iron Enzymes. Inorg. Chem..

[108] Mitchell A. J., Dunham N. P., Martinie R. J., Bergman J. A., Pollock C. J., Hu K., Allen B. D., Chang W.-c., Silakov A., Bollinger J. M., Krebs C., Boal A. K. (2017). Visualizing
the Reaction Cycle in an Iron­(II)- and 2-(Oxo)-glutarate-Dependent
Hydroxylase. J. Am. Chem. Soc..

[109] Kissman E. N., Kipouros I., Slater J. W., Stone E. A., Yang A. Y., Braun A., Ensberg A. R., Whitten A. M., Chatterjee K., Bogacz I., Yano J., Martin Bollinger J., Chang M. C. Y. (2026). Dynamic Metal Coordination Controls Chemoselectivity
in a Radical Halogenase. Nat. Chem. Biol..

[110] Mehmood R., Vennelakanti V., Kulik H. J. (2021). Spectroscopically
Guided Simulations Reveal Distinct Strategies for Positioning Substrates
to Achieve Selectivity in Nonheme Fe­(II)/α-Ketoglutarate-Dependent
Halogenases. ACS Catal..

