# Correction to “Experimental and Computational Elucidation of C(*sp* ^3^)–H Fluorination Barriers in an Iron(II)- and 2‑Oxoglutarate-Dependent Halogenase”

**DOI:** 10.1021/jacs.6c18611

**Published:** 2026-10-08

**Authors:** Vishal Yadav, Chao Wang, Christopher J. Pollock, Jiang Ren, Evan J. Burke, Chi-Yun Lin, Jeffrey W. Slater, Xiaojun Li, Irene Schaperdoth, Wei-Chen Chang, Elvira R. Sayfutyarova, Alexey Silakov, Carsten Krebs, J. Martin Bollinger

**Correction**

The Acknowledgment section of the published article was incomplete. The following text should be added to the Acknowledgment section:

″This work was supported by the National Institutes of Health (R35GM127079 to C.K). A.S. acknowledges grant support from the National Institute of General Medical Sciences (R01GM141284). J.W.S. acknowledges a training fellowship from the National Institutes of Health (F32GM136156). This work is based on research conducted at the Center for High-Energy X-ray Sciences (CHEXS), which is supported by the National Science Foundation (BIO, ENG, and MPS Directorates) under award DMR-2342336. The calculations were performed using the computational resources provided by The Pennsylvania State University’s Institute for Computational and Data Sciences (ICDS).″

