# Supplementary material for: Experimental and Computational Elucidation of C(sp 3)–H Fluorination Barriers in an Iron(II)- and 2‑Oxoglutarate-Dependent Halogenase
Source: J Am Chem Soc. 2026 Jul 30;148(32):34138–53. doi: 10.1021/jacs.6c03646 (PMC13495762; doi:10.1021/jacs.6c03646)
Supplement: Supplementary file 1 [file ja6c03646_si_001.pdf]

Supporting Information

Experimental and Computational Elucidation of C(sp^3)–H Fluorination Barriers in an Iron(II)- and 2-Oxoglutarate-Dependent Halogenase

Vishal Yadav,*[,a] Chao Wang,[a] Christopher J. Pollock,[c] Jiang Ren,[a] Evan J. Burke,[a] Chi-Yun Lin,[a] Jeffrey W. Slater,[a] Xiaojun Li,[d] Irene Schaperdoth,[a] Wei-Chen Chang,[d] Elvira Sayfutyarova,[a] Alexey Silakov,[a, b] Carsten Krebs,*[, a, b] J. Martin Bollinger, Jr.*[, a ,b]

Department of Chemistry[a] and Department of Biochemistry and Molecular Biology[b], The Pennsylvania State University, University Park, Pennsylvania 16802, United States.

Cornell High Energy Synchrotron Source, Wilson Laboratory, Cornell University, Ithaca, New York 14853, United States.[c]

Department of Chemistry, North Carolina State University, Raleigh, North Carolina 27695, United States. [d]

***Address corresponds to**

Vishal Yadav: vpy5018@psu.edu, Carsten Krebs: cdk10@psu.edu, J. Martin Bollinger, Jr. : jmb21@psu.edu

Table of Contents

Experimental Procedures ………………………………………………………………………………… ...S3 – S10

Supporting Figures ………………………………………………………………………………………..S11 – S44

Supporting Tables……………………………………………………………………………………………..S45 – S48

Cartesian Coordinates of the Optimized Structures……………………………………………………….S49 – S123

References……………………………………………………………………………………………………S124 – S129

Experimental Procedures

Materials

All materials were obtained from Sigma-Aldrich, Cambridge Isotope, Fisher Scientific, or VWR (cities, states) unless otherwise noted. All solutions were prepared using 18.2 MΩ/cm deionized water (Barnstead, city).

Methods

Expression, and Purification of BesD.

A plasmid for direct overexpression of N-terminal His_6-tagged BesD was designed and synthesized by Twist Bioscience. The gene encoding *Streptomyces lavenduligriseus* BesD (NCBI Reference: WP_030791981) was codon-optimized for expression in *E. coli*. The synthetic gene was cloned into the pET-28a(+) expression vector using the NdeI and XhoI restriction sites located within the vector multiple cloning site, positioning the insert in-frame with the vector-encoded N-terminal His_6 tag. The insert was introduced with NdeI at the 5′ end (encompassing the ATG start codon) and XhoI at the 3′ end downstream of the stop codon. The final construct was verified by Sanger DNA sequencing at the Genomics Core Facility at Penn State University.

His_6-BesD

ATGCGTCCGGCGTAGAGGATCGAGATCTCGATCCCGCGAAATTAATACGACTCACTATAGGGGAATTGTG
AGCGGATAACAATTCCCCTCTAGAAATAATTTTGTTTAACTTTAAGAAGGAGATATACCATGGGCCACCATC
ACCATCACCATGAAAACCTGTACTTCCAATCCATGAGCAGCAATGGGCAGGAAAGCACCGTCGTCAATCC
TCTGGAGCAGGGCGCACTGCGCCGTATGGCGCATCACTACCACCGGTACGGCATCGCCACCGTCACCG
ATCTGATTCGGGAAGACGTCCGCAAAAACGTGCGGGCGGAGGCGGACCGGCTGCTGGAGAAATACGCC
GAGCGGCGTGATCTCCGCCTCCAGACCACCGGATACACCCGCCGCTCGATGTCCGTGGTGCAGAGCGA
GACGATCGCGGGCAACAGCGAGCTGGTCACCTCGATCTACGCGAATCCGGAACTGCTCGGCGCGCTGG
AGCGCATAGCCGGCGAGAAACTGCACCCCTGCCCCAAGGCCGACGAGGAATTCCTCATCACCCGGCAG
GAACGGAGCGGGGACACGCACGGCTGGCACTGGGGCGATTTCAGCTTCGCCCTCATCTGGGTGCTCCA
GGCCCCGCCCATCGACATCGGCGGCATGCTCCAGTGCGTACCGCACACGGAGTGGGACAAGTCCGATC
CGCGGATCCACCAGTATCTCGTCGACAATCCCATCCACACGTACCACTTCCAGTCGGGCGACGTGTATTT
CCTGCGCACCGACACCACGCTCCACCGCACGGTCCCGCTGCGCGAGGACACCACCCGCATCATCCTGA
ACATGACCTGGGCGGGCGAGCGGGACCTGCGGCGCGAGCTCAAGGGCGACGACCGCTGGTGGGAGGA
CGCCGACGTGCCGGCAGCCGCTGCACTGGATGACTGACTCGAGCACCACCACCACCACCACTGAGATCC
GGCTGCTAACAAAGCCCGAAAGGAA

Protein Sequence

MG<u>HHHHHH</u>ENLYFQS**M**SSNGQESTVVNPLEQGALRRMAHHYHRYGIATVTDLIREDVRKNVRAEADRLLEKY AERRDLRLQTTGYTRRSMSVVQSETIAGNSELVTSIYANPELLGALERIAGEKLHPCPKADEEFLITRQERSGDT HGWHWGDFSFALIWVLQAPPIDIGGMLQCVPHTEWDKSDPRIHQYLVDNPIHTYHFQSGDVYFLRTDTTLHRT VPLREDTTRIILNMTWAGERDLRRELKGDDRWWEDADVPAAAALDD

Protein Purification

The plasmid encoding the His_6-tagged BesD construct was introduced via heat-shock transformation into BL21 (DE3) E. coli cells (New England Biolabs location). A single colony was used to inoculate a starter culture of rich Luria-Bertani (LB) growth medium (35 g/L tryptone, 20 g/L yeast extract, and 5 g/L sodium chloride supplemented with 70 µg/mL kanamycin antibiotic. The culture was incubated for 16-18 h at 37 °C with shaking at 200 rpm. Large culture flasks containing rich LB and kanamycin were inoculated with 35 mL of starter culture per 1 L growth medium and grown at 37 °C with shaking at 200 rpm until an OD_{600} of 1.2-1.5 was reached. Flasks were cooled on ice for 30 min before BesD overexpression was initiated by the addition of IPTG to a final concentration of 0.25 mM. Cultures were left to overexpress for 16-18 h at 18 °C with shaking at 200 rpm. Cells were harvested by centrifugation at 5,000 × *g* and flash frozen in liquid N_2 before storage at -80 °C.

All purification steps were performed at 4 °C. Cell paste was resuspended in 4 mL/g of lysis buffer containing 50 mM sodium HEPES,160 mM $MgSO_4$, and 10 mM imidazole at pH 7.5. Lysozyme (from chicken egg white) (1 mg/g of pellet) and DNase (bovine pancreas) (0.5 mg/g of pellet) were added to the thawed cell suspension. Cells were lysed by sonication (QSonica 750 W, 20 kHz, 5 s pulse, 15 s off, 60% amplitude, 8 min total sonication time). Cell debris was removed by centrifugation for 60 min at 22,000 × *g*, and the supernatant was loaded onto a Ni‑NTA agarose column in lysis buffer (~ 1 mL Ni‑NTA resin per g cell paste). The column was washed twice with 5 column volumes (CVs) of the same buffer (50 mM sodium HEPES,160 mM $MgSO_4$, and 10 mM imidazole at pH 7.5), and the protein was eluted by repeated applications of 5 CVs of the same buffer containing 50 mM sodium HEPES,160 mM $MgSO_4$, and 250 mM immidazole at pH 7.5. Samples from each step were analyzed by SDS-PAGE with Coomassie Brilliant Blue staining; the BesD fusion protein was identified by its molecular weight (MW) of 30.8 kDa. Elution fractions containing BesD were pooled and concentrated in a centrifugal concentrator with a 10 kDa molecular-weight-cutoff (MWCO) filter (Pall Corp., Port Washington, NY) before being dialyzed against 4 L of buffer containing 100 mM sodium HEPES (pH 7.5), and 10 mM EDTA for 4 hours to remove iron. Two additional rounds of diaLysis (4 L for 4 hours) with equivalent buffer lacking EDTA were carried out to remove the metal chelator. Protein purity was assessed by SDS‑PAGE with Coomassie Brilliant Blue staining, and protein concentration was determined by its absorbance at 280 nm using a calculated molar absorptivity of 52,035 $M^{-1}cm^{-1}$ (https://web.expasy.org/protparam/). Typical protein yields were ~ 0.2 g BesD per L of culture medium.

To generate BesD variants, we used the plasmid encoding the His_6-tagged wild-type BesD as a PCR template for site-directed mutagenesis.[1] We used a published protocol that utilizes "inverse PCR" and type IIS restriction enzymes. Primers were designed to include the mutagenized codon and a leading BsaI restriction site. Q5® High-Fidelity DNA Polymerase was used for the PCR reaction, and the product was purified with a PCR cleanup kit before being subjected to several enzymatic digests in a single tube without intervening purification. First, digestion by BsaI-HF®v2 removed the incorporated recognition sequences and created 4-base overhangs of complementary sequence enabling ligation with the vector upon treatment with T4 DNA Ligase. Heat denaturation of the two enzymes was followed by digestion with DpnI to remove template strands. After a second heat denaturation of the DpnI, the reaction mix was used directly to transform NEB® 5-alpha Competent E. coli cells. Subsequent amplification and plasmid purification were carried out according to the manufacturer's protocols. The primers used are listed in **Table S6** below. The coding sequences of all expression vectors were verified by Sanger sequencing at the Genomics Core Facility at Penn State University.

Anion Exchange Chromatography to Remove Chloride Ions from Lysine Stocks

To ensure non-native fluoride binding in various experiments, it was essential to remove Cl^- from commercial L-Lysine stocks. While L-Lysine with natural isotopic abundance was accessible (Sigma-Aldrich) in its free amino acid form (i.e., chloride-free), the deuterium isotopologs were available only as hydrochloride salts.The d_4-4,4,5,5-L-Lys•2HCl stock (Cambridge Isotopes, location) was dissolved in a 25:75 solution of 1M NaOH:100 mM sodium HEPES, pH 7.5 buffer to a final concentration of 0.5 M, yielding a solution of pH 6-8. The final pH was confirmed to be near neutrality by use of pH-indicator strips (EMD Millipore). A 5-mL FF Q-column (Cytiva) was prepared by rinsing the column with 25 mL of 1 M ammonium acetate, pH 6.5, followed by 25 mL of H_2O using a syringe or peristaltic pump. The high excess of the more weakly adsorbing acetate outcompeted and exchanged with the resin-bound Cl^-. The d_4-L-Lys stock was loaded onto and washed off the column with 10 mL H_2O, and the flowthrough was collected. Chloride ions in the L-Lysine solution were expected to bind to the Q resin and release the previously resin-bound acetate ions. The flowthrough solution was flash frozen in liquid N_2 and lyophilized for > 18 h. The solid (presumably the d_4-4,4,5,5-L-Lys •2CH_3COONa saltwas redissolved in H_2O. A similar method was to other substrates used in this work that were available only as chloride salts.

Absorption Spectroscopy

Ultraviolet-visible absorption spectra were recorded with an Agilent 8453 UV-visible spectrophotometer housed in an MBraun (Stratham, NH) anoxic chamber. The absorbance at 800 nm was subtracted from each spectrum to set its baseline to zero.

Stopped-flow Ultraviolet-visible Absorption Spectroscopy (SF-Abs)

SF-Abs experiments were carried out at 5 °C in an Applied Photophysics Ltd. (Leatherhead, UK) SX20 instrument housed in an anoxic chamber (Labmaster, MBraun, Stratham, MA, USA). The instrument was configured for single-mixing with an optical path length of 1 cm, and the data were acquired using a

photomultiplier tube (PMT) detector. In a typical experiment, BesD reactant complex was formed by mixing BesD with aqueous ferrous ammonium sulfate, 2-oxoglutarate (2OG), substrate (e.g, L-Lysine) and halide ion (originating from the most appropriate salt) dissolved in the reaction buffer (100 mM HEPES, pH = 7.5). The light purple solution was loaded into one syringe to be rapidly mixed with an air-saturated solution of the reaction buffer (~0.18 mM O_2) in the opposite syringe. Reactant concentrations are provided in the figure legends.

Liquid Chromatography Mass Spectrometry (LC-MS)

Activity assays were carried out to characterize our preparations of BesD under single- and multiple-turnover conditions. Multiple-turnover assays contained 5 mM substrate, 0.1 mM $(NH_4)_2Fe(SO_4)_2{\bullet}6H_2O$, 0.1 mM BesD, 8 mM $Na_2$2OG, 5 mM sodium ascorbate, and 200 mM the desired halide. Reactions were initiated by addition of 2OG, mixed thoroughly to allow for proper oxygenation in ambient air, and allowed to proceed for 4 h at room temperature before being quenched by addition of an equal volume of acetonitrile/0.1% formic acid. After quenching, internal standards of the substrate isotopologue ($^{13}C_6$-$^{15}N_2$-L-Lys ($^{13}C_6$, 99%; $^{15}N_2$, 99%) for most reactions but 4,4,5,5-d_4-L-Lys (^2H, 96-98%) for reactions with $^{13}C_6$-$^{15}N_2$-L-Lys as substrate, Cambridge Isotope Laboratories, Inc) and sodium d_4-succinate were added to each reaction before they were filtered by using centrifugal filters (VWR, 10 kDa, 500 μL). The samples were immediately transferred to screw cap vials (MICROSOLV, 300 μL) and analyzed by Liquid Chromatography Mass Spectrometry (LC-MS), as described below. Single-turnover assays contained 1 mM substrate, 0.5 mM $(NH_4)_2Fe(SO_4)_2{\bullet}6H_2O$, 0.7 mM BesD, 0.375 mM $Na_2$2OG, and 200 mM of the desired halide. Reactions were prepared inside an anoxic chamber and initiated by the addition of O_2-saturated buffer (5 °C) for a final O_2 concentration of 0.6 mM. Samples were treated identically to the multi-turnover assays. The samples were immediately transferred to screw cap vials (MICROSOLV, 300 μL) and analyzed by LC-MS, as described below.

Analysis by liquid chromatography coupled to mass spectrometry (LC-MS) was carried out on a 1200 series LC system connected to a 6400 series triple quadrupole mass spectrometer (Agilent Technologies). Analysis of L-Lys-based products by LC-MS was performed by injection (5 µL) of the reaction solution onto a SeQuant ZIC-HILIC (3.5 µM, 150 x 2.1 mm; EMD Millipore) column with a mobile phase constituted from (A) 10:90 (v/v) MeCN:H_2O, 10 mM NH_4HCO_2, pH 5.0 and (B) 90:10 (v/v) MeCN:H_2O, 10 mM NH_4HCO_2, pH 5.0. The column was developed at a flow rate of 0.2 mL/min with a linear gradient of 95% B to 30% B over 25 min followed by a linear gradient of 30% B to 95% B over 5 min. Samples were analyzed in positive-ion mode. Evaluation of succinate present in reactions was performed by injection (2 µL) onto an Extend-C18 (1.8 µM, 50 x 4.6 mm; Agilent) column with a mobile phase constituted from (A) 0.1% formic acid in H_2O and (B) MeCN. Isocratic elution with 5% B at 0.3 mL/min for 5 min was to elute analytes. Negative-ion mode was used for MS detection. Retention times and *m/z* values for each analyte are shown in main figures as well as in SI figures below.

Freeze-Quench Mössbauer Spectroscopy

Freeze-quench Mössbauer samples were prepared according to previously published procedures.[2] Mössbauer spectra were recorded on a spectrometer from SEECO (Edina, MN) equipped with a Janis SVT-400 variable-temperature cryostat. The reported isomer shift (δ) is given relative to the centroid of the spectrum of α-iron metal at room temperature. External magnetic fields were applied parallel to the direction of propagation of the γ-radiation. Mössbauer spectra were simulated with WMOSS spectral analysis software from SEECO (www.wmoss.org, SEE Co., Edina, MN). Samples were generated either directly from anoxic solutions of BesD, $^{57}Fe(II)$, $Na_2$2OG, d_4-L-Lys, and NaCl/NaF in a buffer solution of 100 mM sodium HEPES (pH 7.5) or by (1) rapidly mixing this reactant solution at 5 ±1 °C with an equal volume of the same buffer that had been saturated with O_2 (~1.8 mM) and (2) rapidly freezing the reaction at the desired reaction time (**Figure 5**) by injecting it into cryogenically cooled (-150 °C) 2-methylbutane (for reaction times < 30 s). Samples representing longer reaction times (>30 seconds) were frozen by pipetting the reaction solution into a Mössbauer cell cooled on a metal block that was in contact with liquid N_2. Reactant concentrations after mixing are provided in the figure legends.

Preparation of the $\{FeNO\}^7$ Samples

After removal of O_2 from the stock solutions, BesD, Fe(II), X^- (X = Cl, Br or F), 2OG, and L-Lysine substrate were mixed in a MBraun (Stratham, NH) anoxic chamber to final concentrations of 0.7, 0.5, 1000, 5, and 10 mM, respectively. The resulting solution was returned to the Schlenk line; the flask was briefly evacuated to ∼100 Torr and slowly refilled with nitric oxide gas (Messer) to ~300 Torr (*caution: pressure higher than 300 Torr caused protein precipitation*). Under this atmosphere, the protein was incubated on ice with brisk stirring for 10 min, as the dark yellow color developed. The resulting solution was transferred back into the anoxic chamber and divided to fill both an X-band and a Q-band EPR tube. The solutions were then frozen in liquid nitrogen.

Preparation of the Vanadyl Samples

Samples of the BesD•vanadyl complex contained 0.8 mM vanadyl sulfate, 0.7 mM BesD, 20 mM sodium succinate, and 0.4 M sucrose (cryosolvent) in 100 mM sodium HEPES, pH 7.5. Concentrations of L-Lys and NaCl/CsF are given in the figures.

Electron Paramagnetic Resonance Spectroscopy

EPR samples were transferred into X-band EPR tubes (Quartz Scientific, Inc., Fairport Harbor, Ohio), and continuous-wave X-band EPR spectra were recorded on a Magnettech MS5000X spectrometer equipped with an Oxford Instruments ESR-900 continuous flow cryostat and an Oxford Instruments ITC-300 temperature controller. For samples of BesD•vanadyl complexes, data were collected at 35 K from 250 to 450 mT. For samples with iron, data were collected at 10 K from 50 to 425 mT. Spectra were collected with a modulation amplitude of 1 mT, a microwave power of 0.1 mW, and a microwave frequency of 9.43516 GHz. Reactant concentrations are provided in the figure legends. The collected spectra were baseline corrected by subtraction

of a fitted polynomial in Kazan Viewer.[3] The baseline-corrected EPR spectra were simulated with the EasySpin toolbox[4] within MATLAB.

X-ray Absorption Spectroscopy

HERFD XAS Data Collection and Processing

HERFD XAS data were collected at the Photon-In Photon-Out X-ray Spectroscopy (PIPOXS) beamline at the Cornell High Energy Synchrotron Source (CHESS), operating under conditions of 125 mA at 6 GeV. The incident x-ray beam was energy selected using a cryogenically-cooled Si(311) monochromator and was calibrated to the first inflection point of an Fe metal foil (7112.0 eV). The beam was focused to a size of 250 x 500 μm at the sample using a pair of in-hutch Rh-coated focusing mirrors. All samples were maintained at 80 K in a liquid nitrogen flow cryostat and placed at 45° relative to the incident beam. The peak of the Fe $K\alpha_1$ emission line was selected using five spherically-bent Ge(440) analyzer crystals and detected using a Pilatus 100K detector aligned on Rowland geometry using the DAVES spectrometer.[5] The space between the sample, analyzers, and detector was filled with helium to minimize signal attenuation. All samples were checked for damage by collecting rapid XANES scans on the same sample spot and monitoring for changes in the edge shape or position. No damage was observed for any sample after 21 minutes of exposure, and so XAS scans lasting for 430 seconds were collected in continuous scanning mode over the range from 7000 – 7450 eV; three of these scans were performed on each sample spot. All scans were assessed for homogeneity and averaged together using PyMCA,[6] after which the spectra were imported to Athena[7] and normalized to an edge jump of 1. These normalized spectra were fit with BlueprintXAS 4.1.4[8] using a minimum number of pseudo-Voight peaks; several dozen individual fits were generated for each sample spectrum, and the average pre-edge areas and intensity-weighted average energies (IWAE) of all physically reasonable fits are reported.

Computational Methods

System Setup. The initial structure of the L-lysine 4-chlorinase, BesD, was obtained from the X-ray crystal structure (PDB code: 6NIE, chain A of the tetramer).[9] Missing hydrogen atoms were added using the Leap module of the Amber18 software package.[10] The protonation states of the titratable residues (Asp, Glu, His) were assigned based on predicted pK_a values from the PROPKA program and through inspection of hydrogen-bonding networks and metal-binding environments.[11] In BesD, all histidine residues were singly protonated (His27, His112, His136 and His203 at the δ position; His41, His96, His133, His164, and His187 at the ε position). All Asp and Glu residues were deprotonated in 6NIE.

The L-Lys and 2OG cosubstrates were parameterized using the generalized AMBER force field (GAFF),[12] with partial atomic charges derived by the restrained electrostatic potential (RESP) method at the HF/6-31G* level using the Gaussian 16 program.[13-17] The Amber ff14SB force field[18] was applied to standard protein residues, while the Metal Center Parameter Builder (MCPB.py v3.0) in AmberTools18[19, 20] was used to generate force field parameters for the iron active site, both in the presence and absence of molecular oxygen. To ensure charge

neutrality for the system, Na^+ counterions were added to the surface of the protein. Finally, the entire system was solvated in a TIP3P water box, ensuring a minimum distance of 10 Å between the protein surface and the box boundary.

MD Simulations. Following the initial system setup, energy minimization was performed using a combination of the steepest descent and conjugate gradient methods.[21] The system was then gradually heated from 0 to 300 K over 50 ps in the NVT ensemble, with a positional restraint of 15 kcal/mol·Å² applied to the enzyme. Density equilibration was conducted for 1 ns in the NPT ensemble to achieve a uniform density. This step employed the Langevin thermostat (collision frequency: 2 ps)[22] to maintain a temperature of 300 K and the Berendsen barostat (pressure relaxation time: 1 ps)[23] to regulate the pressure at 1 atm. Subsequently, all systems underwent an additional 4 ns equilibration in the NPT ensemble without restraints. The production MD simulation was performed for 200 ns with a 2 fs time step under NPT conditions. The SHAKE algorithm[24] was applied to constrain bonds involving hydrogen atoms. All MD simulations were conducted in the Amber18 software package.

QM/MM Simulations. To obtain representative structures for QM/MM studies, clustering analysis was performed on the MD trajectories. The analysis confirmed that the selected snapshots closely resembled those from the most populated clusters. QM/MM simulations were conducted using the ChemShell program,[25, 26] which uses DL_POLY for the MM region and Turbomole for the QM region.[27, 28] The polarizing effect of the protein environment on the QM region was accounted for using an electrostatic embedding scheme.[29] The QM/MM boundaries were capped with hydrogen link atoms following the charge-shift model.[30] The active region included all residues within 16 Å of the Fe atom.

The QM region was treated with unrestricted density functional theory (DFT) using the B3LYP functional.[31-33] Geometry optimization and potential energy scans were performed with the def2-SVP basis set,[34] while single-point energy calculations employed the larger def2-TZVP basis set.[34] Dispersion corrections were included using Grimme's D4 method.[35] Geometry optimizations were conducted with the DL-FIND optimizer.[36] Transition states were identified from the highest points of the potential energy surface (PES), which was scanned with a small increment of 0.01 Å along the reaction coordinate. This approach is widely used in computational studies of metalloenzyme mechanisms.[37-41] In previous studies,[42] the high-spin open-shell quintet state (S_{total} = 2) was determined to be the ground state, and the same spin state was used for all intermediates and transition states. Structural visualizations were performed in the VMD and CYLview software packages.[43, 44]

QM Region. In the QM/MM calculations, we used various QM regions. In **Figures 7 and 8**, the QM region includes Fe, 2OG, two histidines (His136, His203), the L-Lys substrate, and, when necessary, different anionic ligands: chloride, bromide, or fluoride. In the calculations for Figure 8, the QM region encompasses Fe, two histidines (His136, His203), one asparagine (Asn218), a water molecule, and an oxo atom. Depending on the

specific complex and catalytic step under consideration, it also includes different anionic ligands—either a chloride or a fluoride ion—together with one succinate and the L-Lys substrate. In all QM/MM calculations, methylimidazole was used to model histidines, and acetamide was used to model asparagine.

Mössbauer Spectroscopic Parameters. We computed the Mössbauer isomer shifts (δ) and quadrupole splittings (ΔE_Q) within the ORCA program (version 6.0.1) using DFT with the TPSS0 functional,[45, 46] for the geometries optimized by QM/MM. In these calculations, the scalar relativistic X2C Hamiltonian was employed together with the DefGrid3 integration grid,[47] TightSCF convergence criteria, the RIJCOSX approximation,[48] and first-order picture change effects.[49-52] The Fe atom was described using the *s*-decontracted aug-cc-pVTZ-J basis set,[53] while the x2c-TZVPPall basis set was applied for all other atoms.[54] The ^{57}Fe Mössbauer isomer shift was determined from the electron density at the Fe nucleus, using calibration parameters previously obtained at this level of theory[55]: $\delta_{calc} = \alpha(\rho - C) + \beta$, where α = -0.28 mm s^{-1} au^3, β = 1.1425 mm s^{-1}, and C = 14908 au^3. A standard deviation of ~0.060 mm/s for the isomer shift is expected in DFT calculations of ^{57}Fe Mössbauer spectroscopic parameters.[55] Modeling quadrupole splitting is more challenging, with deviations generally ranging from 0.3 to 0.5 mm/s.[37]

***DFT Calculated XAS Spectra*.** Pre-edge spectra for all species were calculated using time-dependent density functional theory (TD-DFT)[56] as implemented in ORCA v5.0.3.[57, 58] Geometry optimized structures were obtained from PLACE and used without further modification. TD-DFT calculations were performed using the BP86 functional,[59, 60] def2-TZVP basis set,[61] and auxiliary def2/J basis set[61] on all atoms except Fe, for which the expanded CP(PPP) basis set was used.[62] Solvation was included via the CPCM model.[63] A dense integration grid (defGrid3) was used, together with increased integration accuracy on Fe. A sample input file can be found in the Supporting Information. Spectra were plotted using the orca_mapspc utility, with an applied Gaussian broadening of 1 eV to match the experimental resolution, and were shifted by 182.1 eV to account for the known scalar offset between calculated and experimental data.[56, 64, 65]

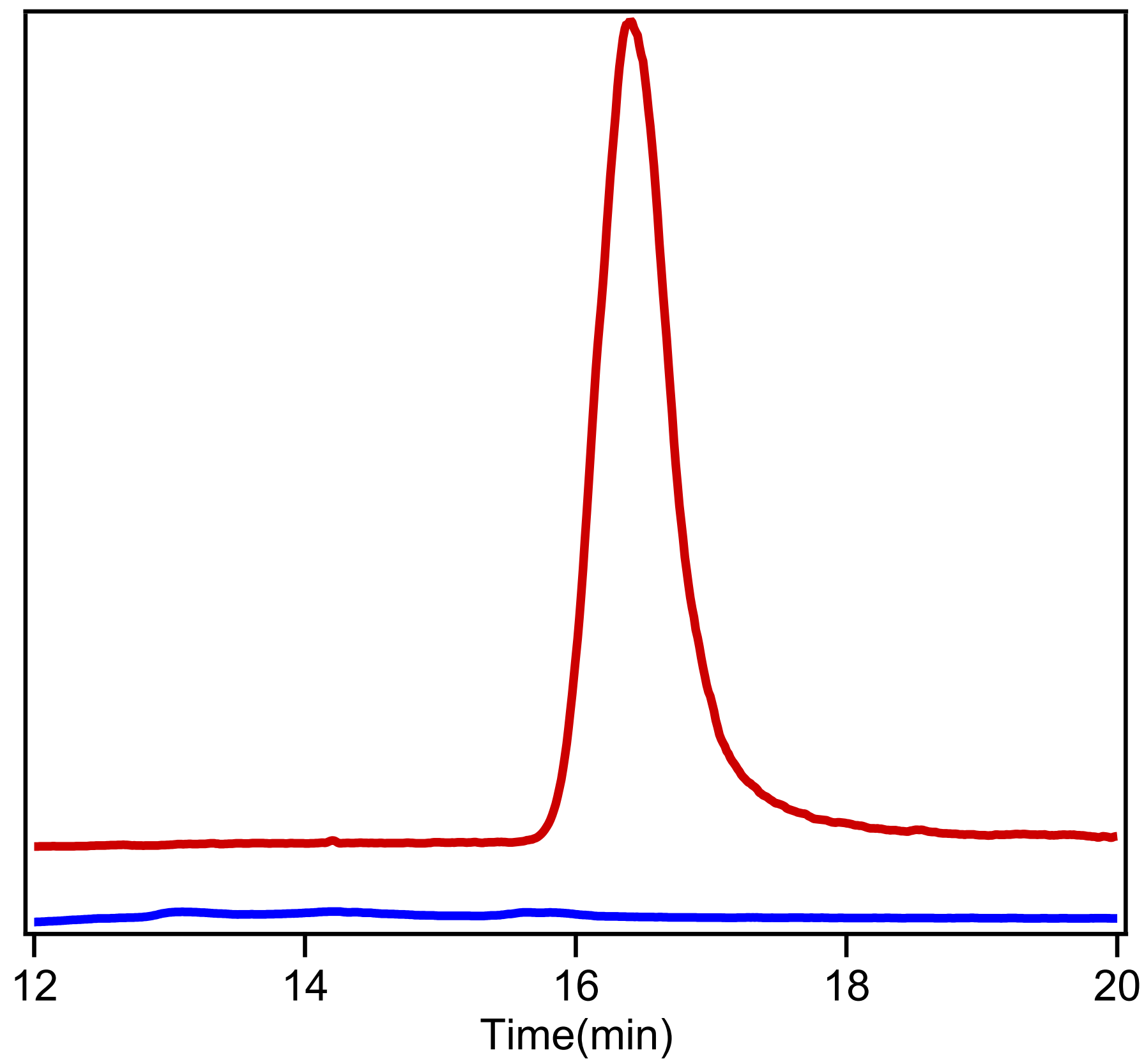

Figure S1. Single-ion LC-MS chromatograms monitoring the 4-OH-L-Lysine product (*m/z* 163.1, red line) and 4-F-L-lysine potential product (*m/z* 165.1, blue) from a reaction initially containing 0.1 mM BesD, 0.1 mM Fe(II), 1 mM L-Lys, 200 mM CsF, 5 mM L-asc, 8 mM $Na_2$2OG, and 0.9 mM O_2 in 100 mM HEPES buffer (pH 7.5) that was incubated at 20 °C for 4 h before being quenched and analyzed as described in Liquid Chromatography Mass Spectrometry (LC-MS) section above.

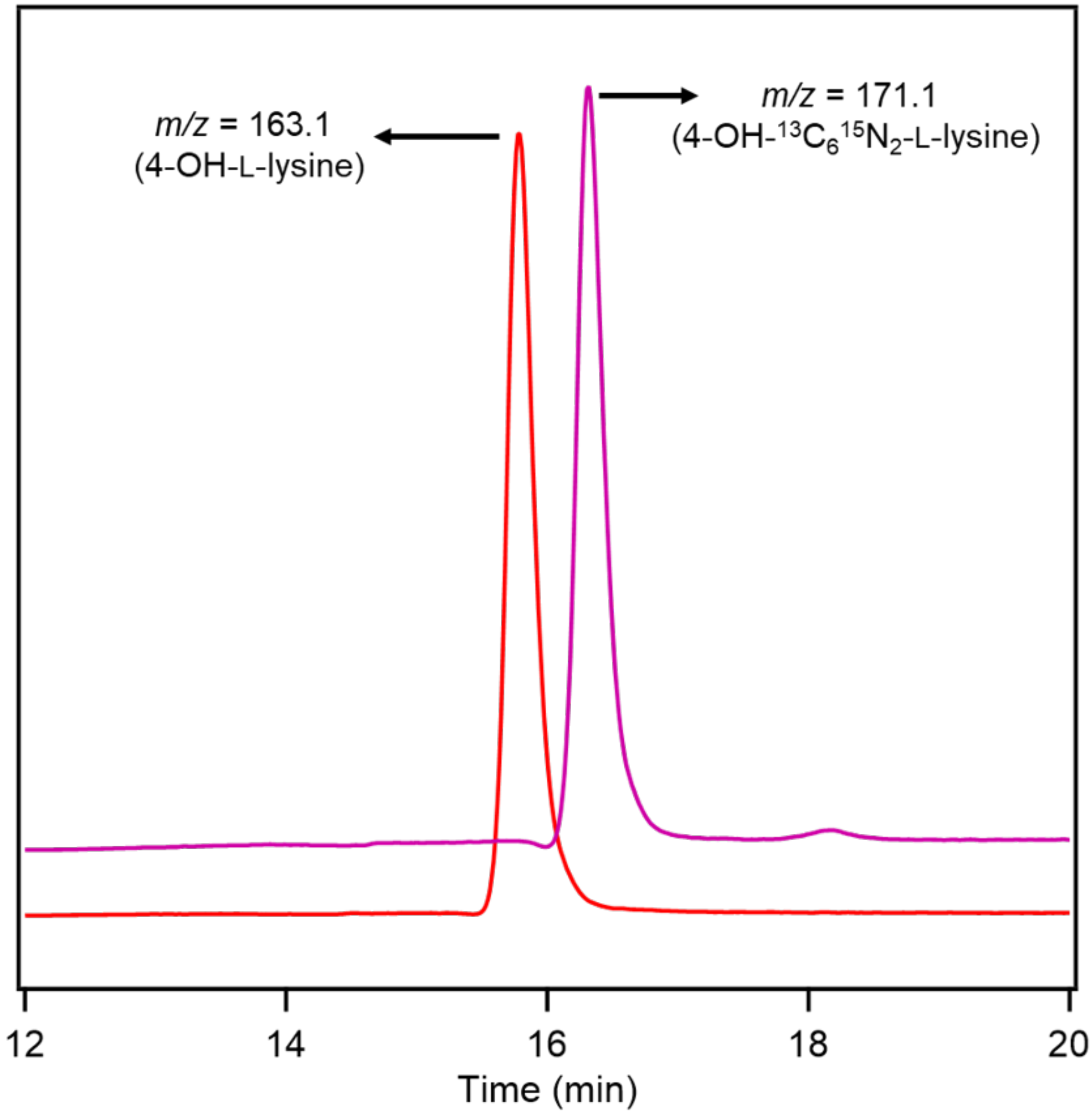

Figure S2. Single-ion LC-MS chromatograms monitoring the 4-OH-L-Lysine product (*m/z* 163.1, red line) and 4-OH-$^{13}C_6$-$^{15}N_2$-L-Lysine (*m/z* 171.1, magenta line) products from a reaction initially containing 0.1 mM BesD, 0.1 mM Fe(II), 1 mM L-Lys, 200 mM CsF, 5 mM L-asc, 8 mM $Na_2$2OG, and 0.9 mM O_2 in 100 mM HEPES buffer (pH 7.5) that was incubated at 20 °C for 4 h before being quenched and analyzed as described in Liquid Chromatography Mass Spectrometry (LC-MS) section above**.**

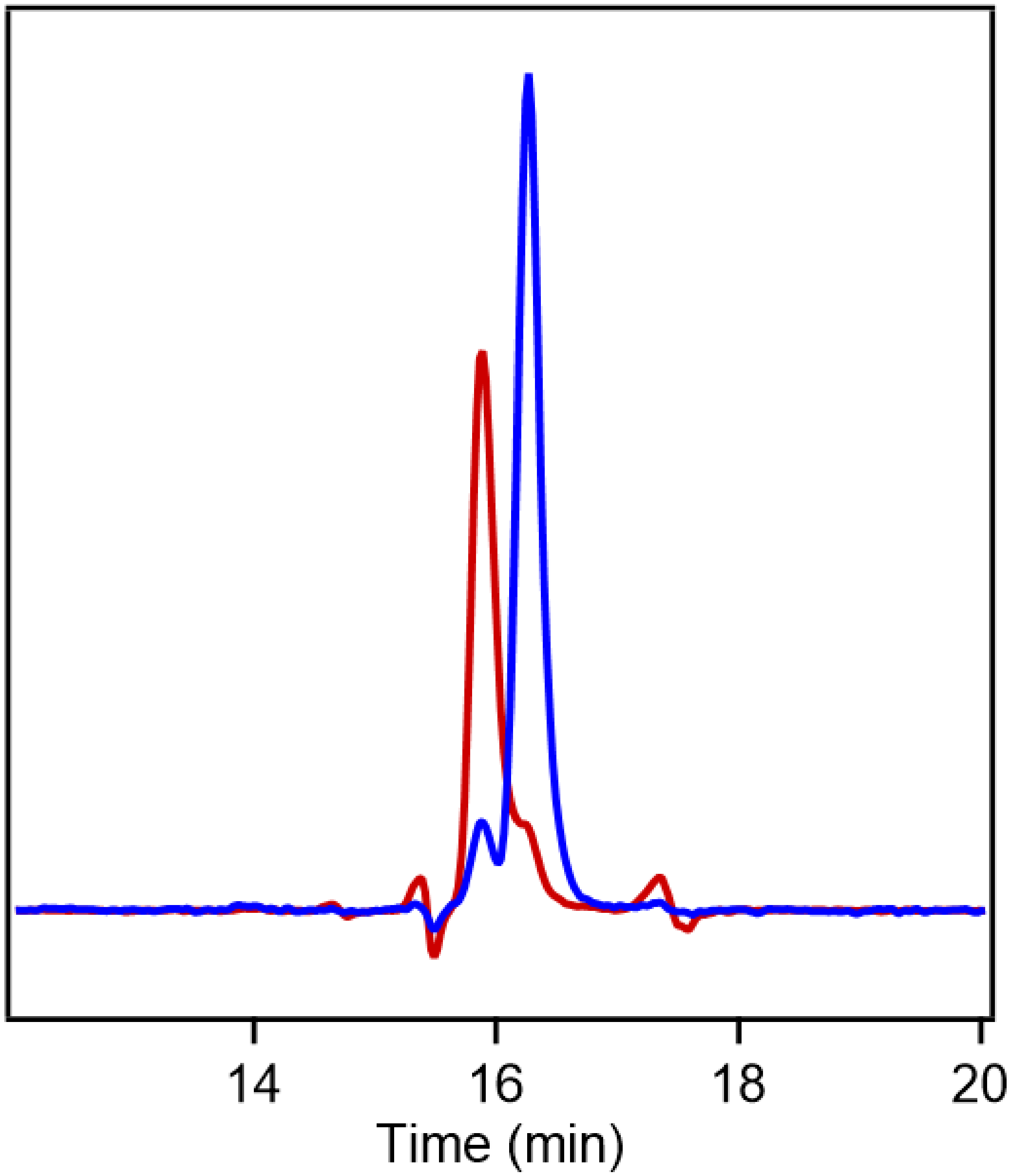

Figure S3. Single-ion LC-MS chromatograms monitoring the 4-OH-4-[^{2}H]-L-Lysine (*m/z* 164.1, red line) and 5-OH-4,4-[^{2}H]$_2$-L-Lysine (*m/z* 165.1, blue line) products from a reaction initially containing 0.1 mM BesD, 0.1 mM Fe(II), 1 mM 4,4-[^{2}H]$_2$-L-Lys, 200 mM CsF, 5 mM L-asc, 8 mM $Na_2$2OG, and 0.9 mM O_2 in 100 mM HEPES buffer (pH 7.5) that was incubated at 20 °C for 4 h before being quenched and analyzed as described in Liquid Chromatography Mass Spectrometry (LC-MS) section above**.**

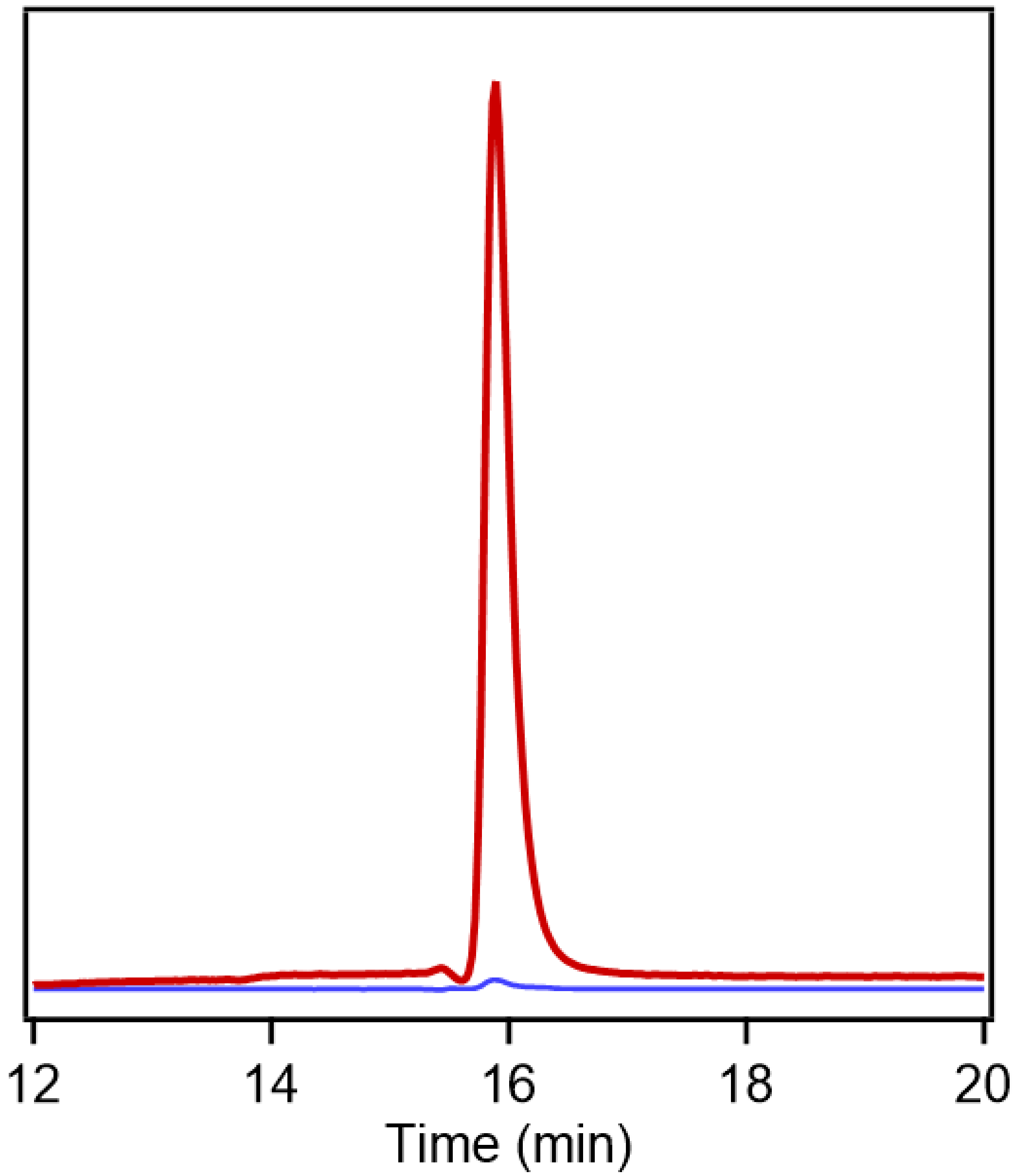

Figure S4. Single-ion LC-MS chromatograms monitoring the 4-OH-5,5-[^{2}H]$_2$-L-Lysine (*m/z* 165.1, red line) and 5-OH-4-[^{2}H]-L-Lysine (*m/z* 164.1, blue line) products from a reaction initially containing 0.1 mM BesD, 0.1 mM Fe(II), 1 mM 5,5-[^{2}H]$_2$-L-Lys, 200 mM CsF, 5 mM L-asc, 8 mM Na$_2$2OG, and 0.9 mM O_2 in 100 mM HEPES buffer (pH 7.5) that was incubated at 20 °C for 4 h before being quenched and analyzed as described in Liquid Chromatography Mass Spectrometry (LC-MS) section above**.**

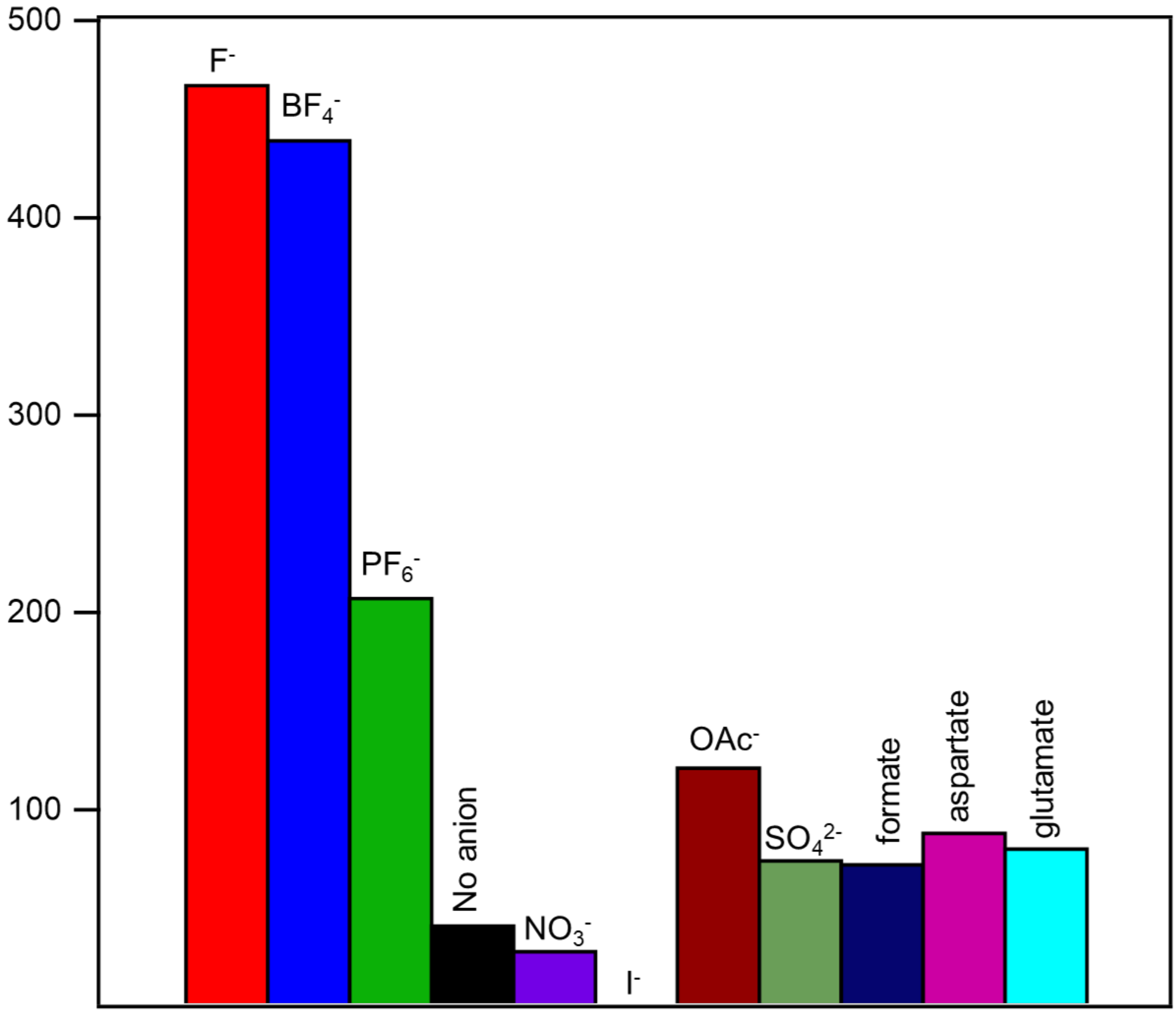

Figure S5. Relative yields of the 4-OH-L-Lysine (*m/z* 163.1) product from a reaction initially containing 0.1 mM BesD, 0.1 mM Fe(II), 1 mM L-Lys, 200 mM X^- (specified anion), 5 mM L-asc, 8 mM $Na_2$2OG, and 0.9 mM O_2 in 100 mM HEPES buffer (pH 7.5) that was incubated at 20 °C for 4 h before being quenched and analyzed as described in Liquid Chromatography Mass Spectrometry (LC-MS) section above**.**

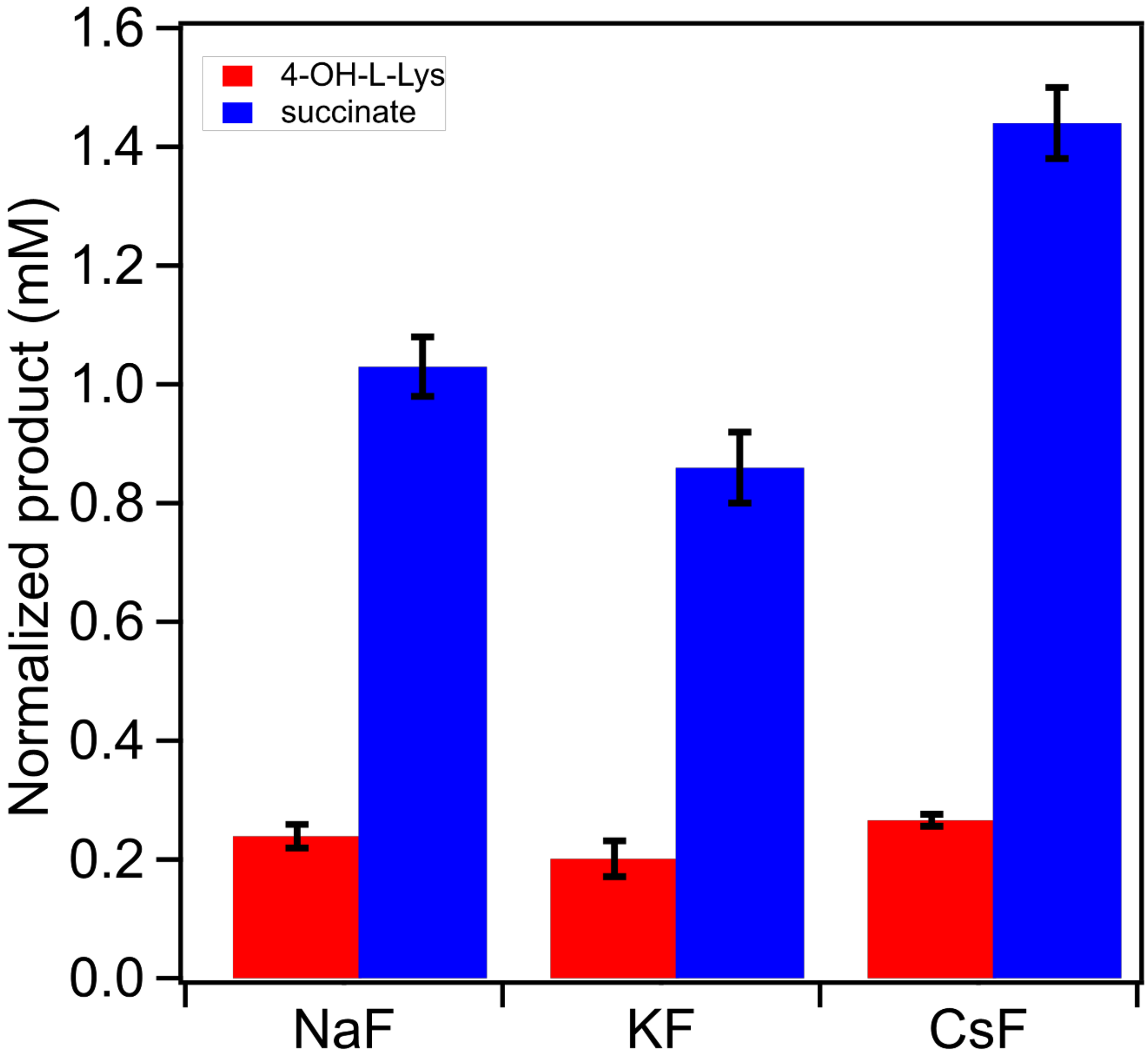

Figure S6. Relative yields of the 4-OH-L-Lysine (*m/z* 163.1) and succinate (*m/z* 117.1) products from a reaction initially containing 0.1 mM BesD, 0.1 mM Fe(II), 1 mM L-Lys, 200 mM M^+F^- (M+ = alkali metal ion) , 5 mM L-asc, 8 mM $Na_2$2OG, and 0.9 mM O_2 in 100 mM HEPES buffer (pH 7.5) that was incubated at 20 °C for 4 h before being quenched and analyzed as described in Liquid Chromatography Mass Spectrometry (LC-MS) section above**.**

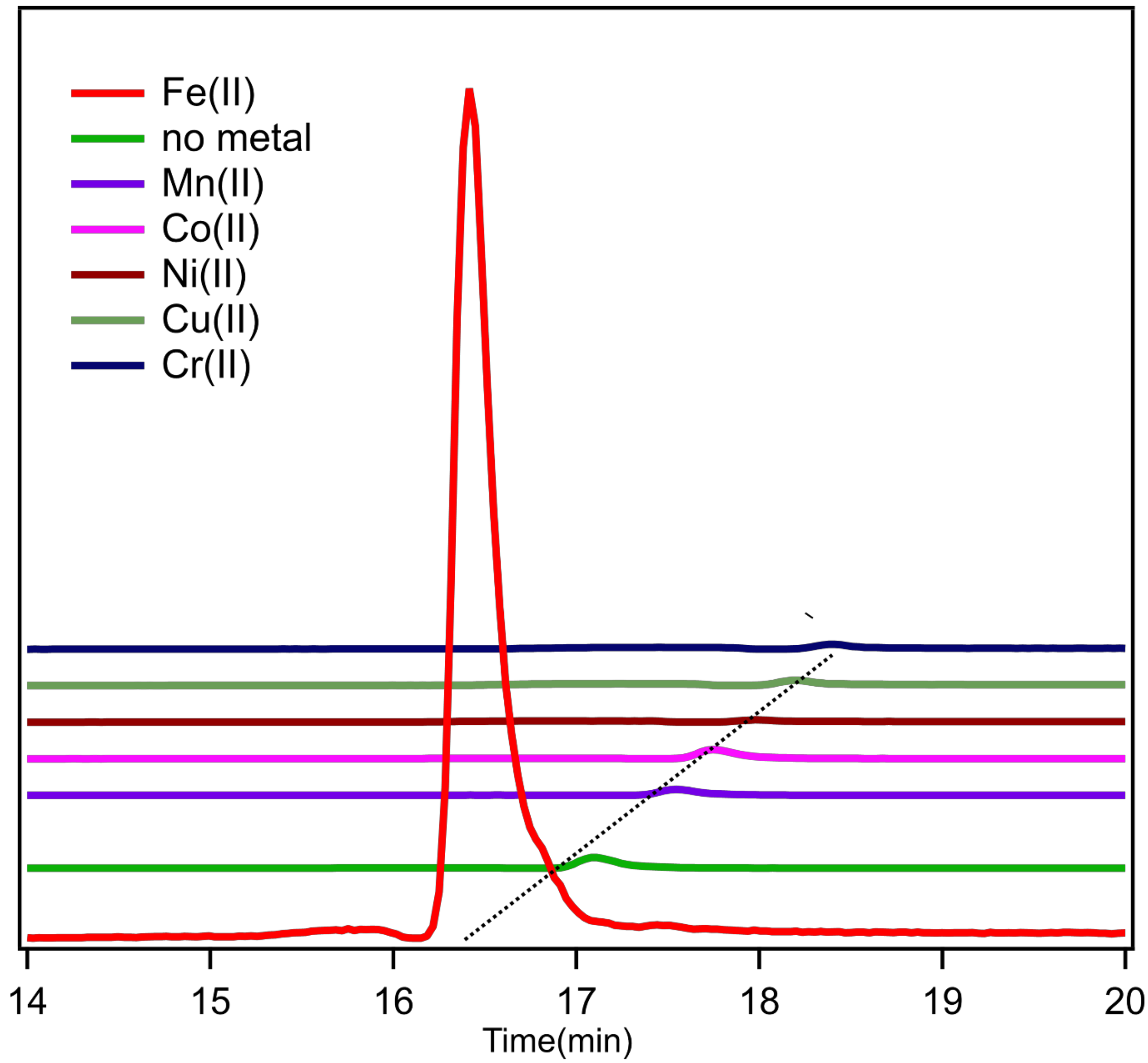

Figure S7. Single-ion LC-MS chromatograms monitoring the 4-OH-L-Lysine (*m/z* 163.1) product from a reaction initially containing 0.1 mM BesD, 0.1 mM M(II) (M = various transition metals), 1 mM L-Lys, 200 mM CsF, 5 mM L-asc, 8 mM $Na_2$2OG, and 0.9 mM O_2 in 100 mM HEPES buffer (pH 7.5) that was incubated at 20 °C for 4 h before being quenched and analyzed as described in Liquid Chromatography Mass Spectrometry (LC-MS) section above.

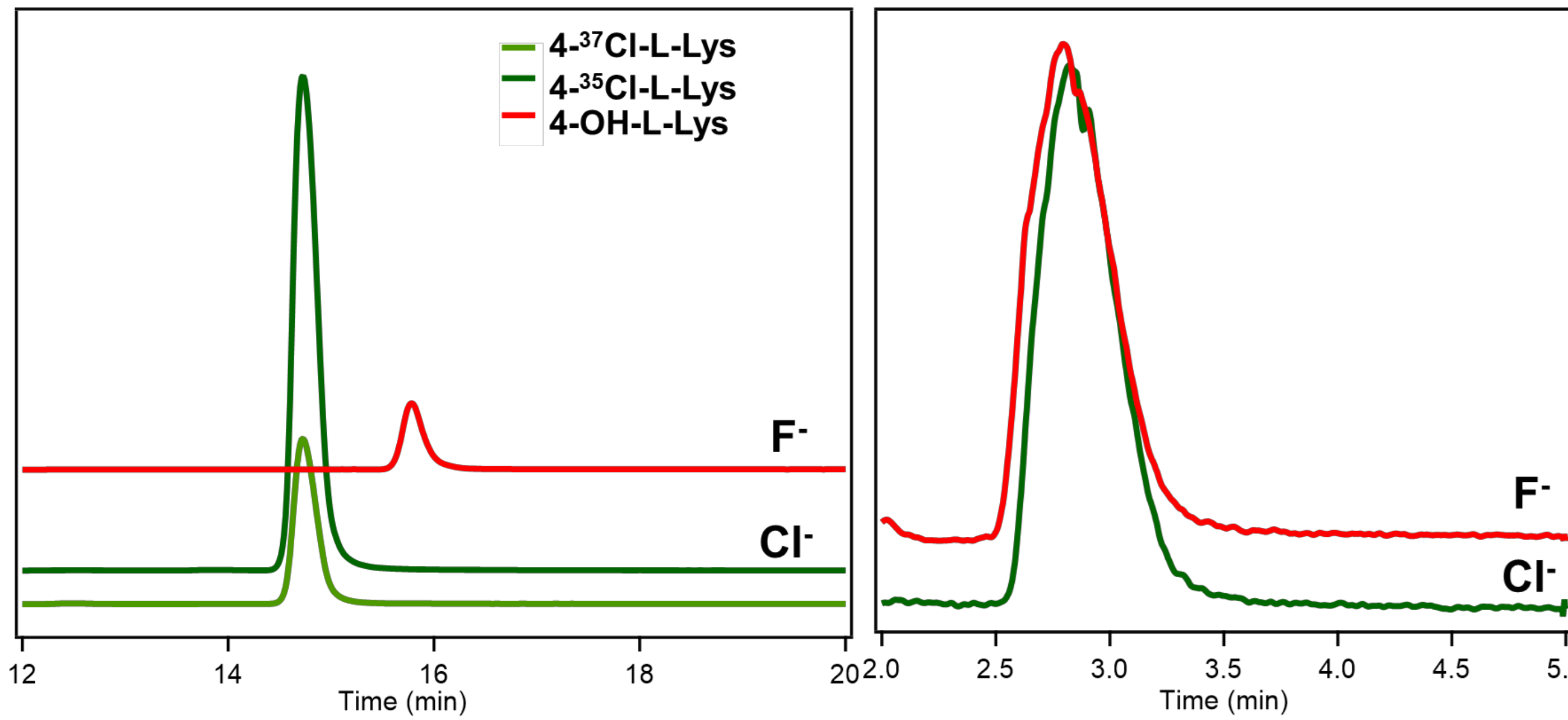

Figure S8. Single-ion LC-MS chromatograms monitoring (*left*) the 4-Cl-L-Lysine (*m/z* 181.1 for ^{35}Cl and 183.1 for ^{37}Cl, green solid traces) and 4-OH-L-Lysine (*m/z* 163.1, red solid trace) and (*right*) succinate (*m/z* 117.1, green and red lines in presence of Cl^- and F^- (from the Cs salt), respectively) products in reactions that initially contained containing 0.1 mM BesD, 0.1 mM M(II) (M = various transition metals), 1 mM L-Lys, 200 mM CsF, 5 mM L-asc, 8 mM $Na_2$2OG, and 0.9 mM O_2 in 100 mM HEPES buffer (pH 7.5) that was incubated at 20 °C for 20 min (for chloride) and 4 h (for fluoride) before being quenched and analyzed as described in Liquid Chromatography Mass Spectrometry (LC-MS) section above**.**

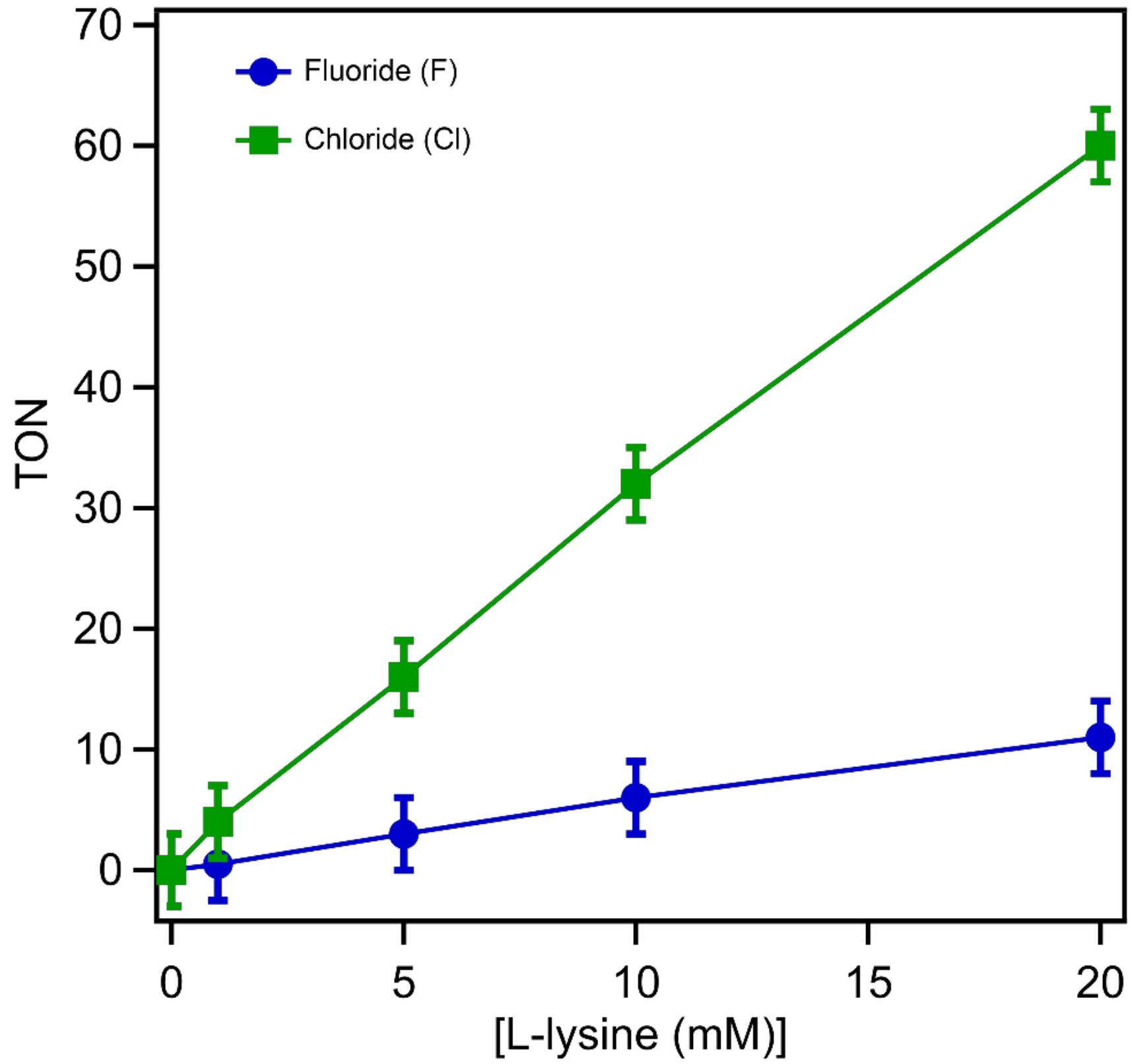

Figure S9. Equivalents of chlorinated (green square) and hydroxylated (blue circle) products in the reactions that initially contained BesD•Fe(II)•L-Lys•NaCl•2OG and BesD•Fe(II)•L-Lys•CsF•2OG respectively with oxygen saturated buffer (100 mM HEPES at pH 7.5) at 25 ^{o}C at different L-Lys concentration. [BesD] = 0.1 mM, [Fe^{II}] = 0.1 mM, [Cl^- or F^-] = 200 mM, [L-asc] = 5 mM, [2OG] = 8 mM and were incubated at 20 °C for 20 min (for chloride) and 4 h (for fluoride) before being quenched and analyzed as described in Liquid Chromatography Mass Spectrometry (LC-MS) section above**.**

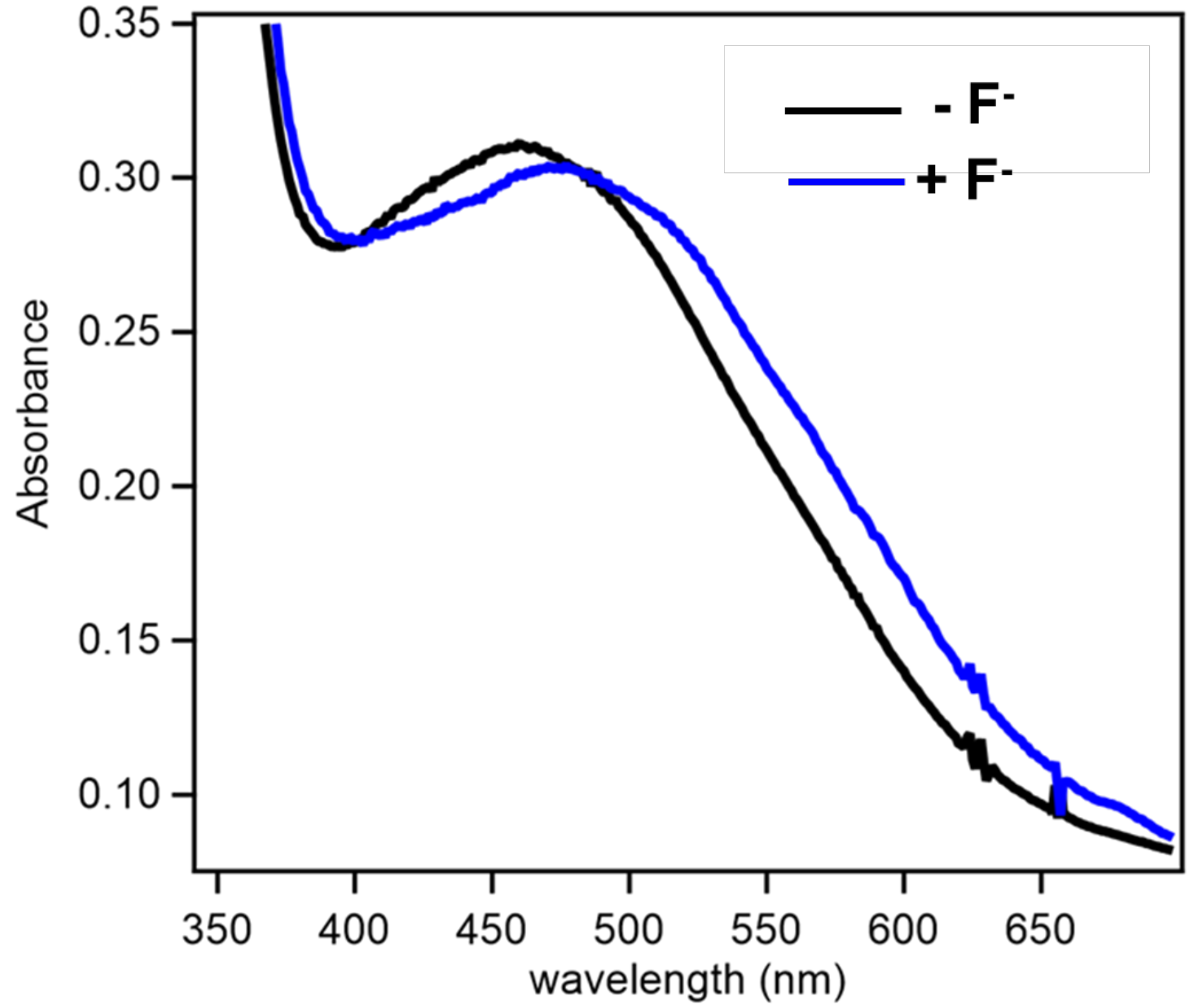

Figure S10. UV-visible absorption spectra of an anoxic solution of 0.5 mM BesD, 0.4 mM Fe^{II}, 5 mM $Na_2$2OG, 10 mM L-Lys with 200 mM CsF (blue line) and without fluoride source (black).

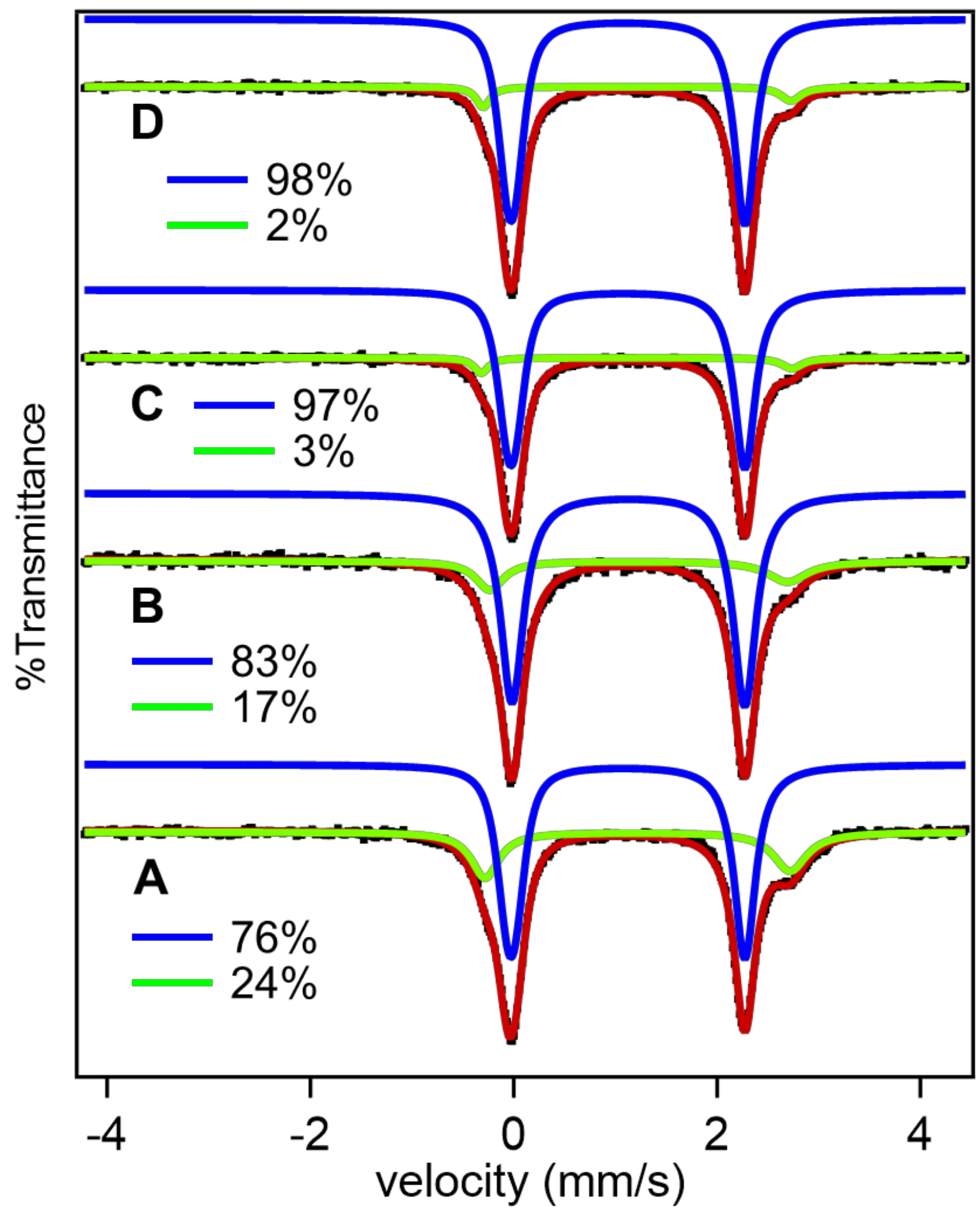

Figure S11. Mössbauer spectra (4.2 K/0 mT) of samples prepared by freezing anoxic solutions containing 1.8 mM BesD, 1.5 mM $^{57}Fe^{II}$, 16 mM 2OG, 30 mM L-Lys, and either 2 mM NaF (***A***), 500 mM NaF (***B***), 1 M NaF (***C***), or 2 M NaF (***D***). Experimental data are represented by black vertical bars, and the red solid lines represent the fits of the experimental spectra as summations of the component spectra shown in blue and green. These component spectra have the parameters: (*blue*) isomer shift (δ) = 1.12 mm/s; quadrupole splitting $|\Delta E_Q|$ = 2.30 mm/s; line widths Γ_L = 0.25 and Γ_R = 0.19 and (*green*) isomer shift (δ) = 1.23 mm/s; quadrupole splitting $|\Delta E_Q|$ = 3.00 mm/s; ine widths Γ_L = 0.17 and Γ_R = 0.25.

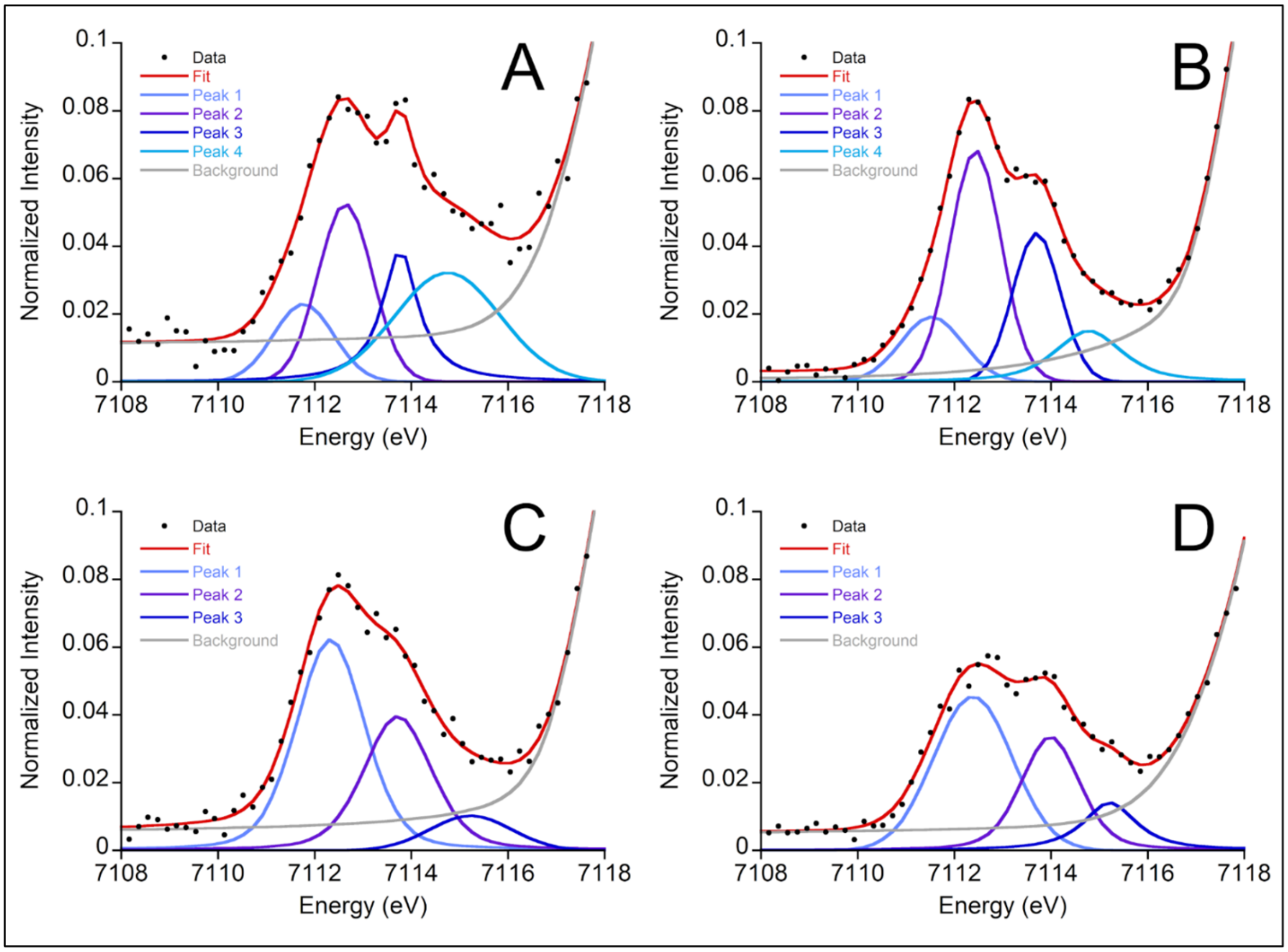

Figure S12. Pre-edge fits of the XAS spectra for the BesD•Fe(II)•2OG•X•L-Lys complex. Panels correspond to X = Cl^- (**A**), Br^- (**B**), F^- (**C**), and no added anion (**D**). Black points represent experimental data, and the red curves show the total fits. Individual Gaussian components used to model the pre-edge features are shown as colored traces, and the gray trace represents the background function. These fitted Gaussian components were used to determine the pre-edge integrated area and the intensity-weighted average energy (IWAE) values reported in **Table 2** in the main text**.**

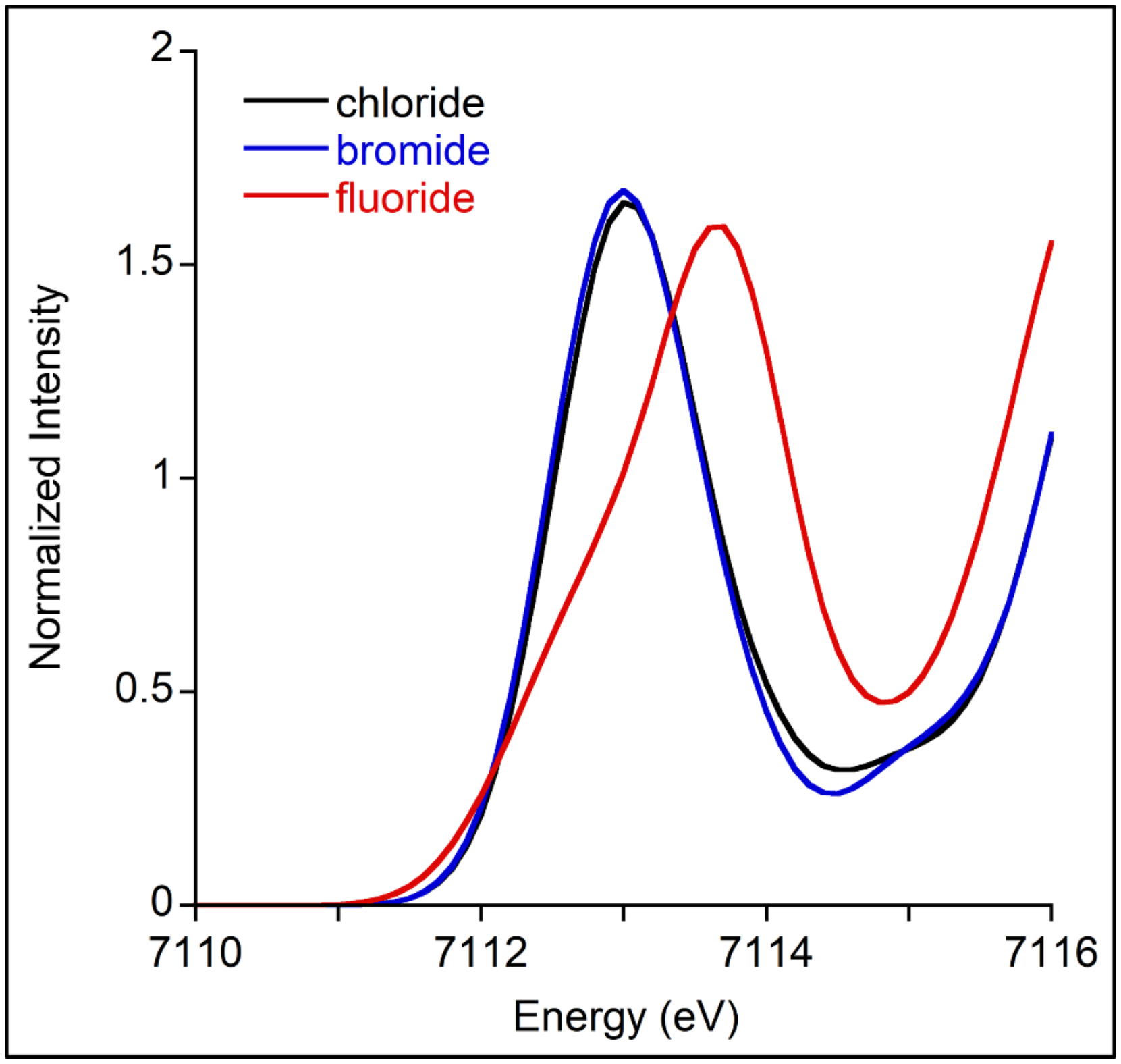

Figure S13. An overlay of the calculated Fe pre-edge spectra of BesD•Fe(II)•2OG• X(X = F, Cl, Br)•L-Lys.

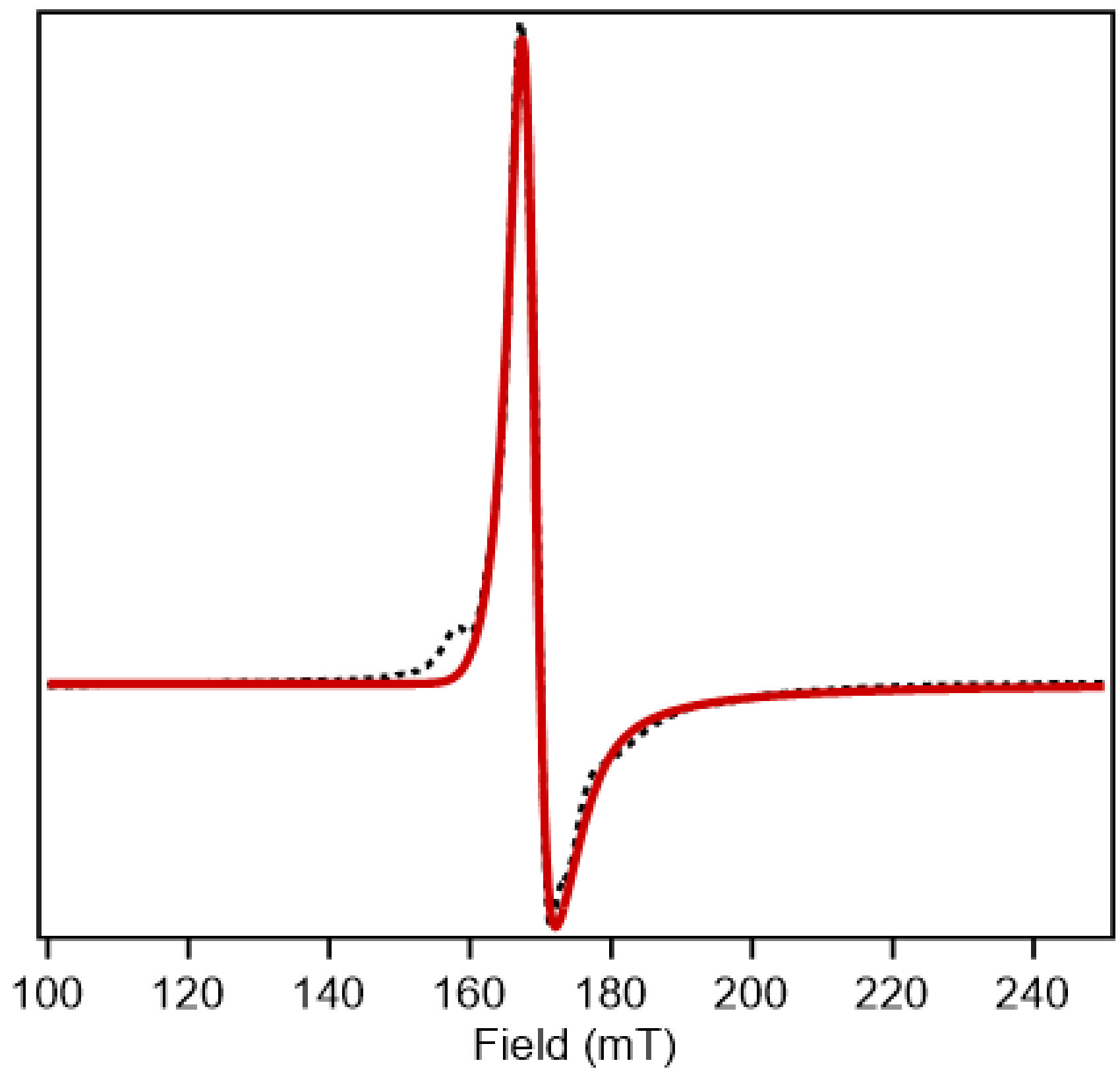

Figure S14. Continuous-wave, X-band EPR spectrum the BesD•{FeNO}[7]•2OG•L-Lys. The experimental spectrum is shown as black dots, and the simulation is shown as the red solid line. The spectrum was collected at 12 K with a 1 mT modulation amplitude, 20 dB microwave power, and 9.435165 GHz microwave frequency. Simulation parameters: $g_x = g_y = 4.231$, $g_z = 2.001$, g-strain-x = g-strain-y = 0.007, g-strain-z = 0.005.

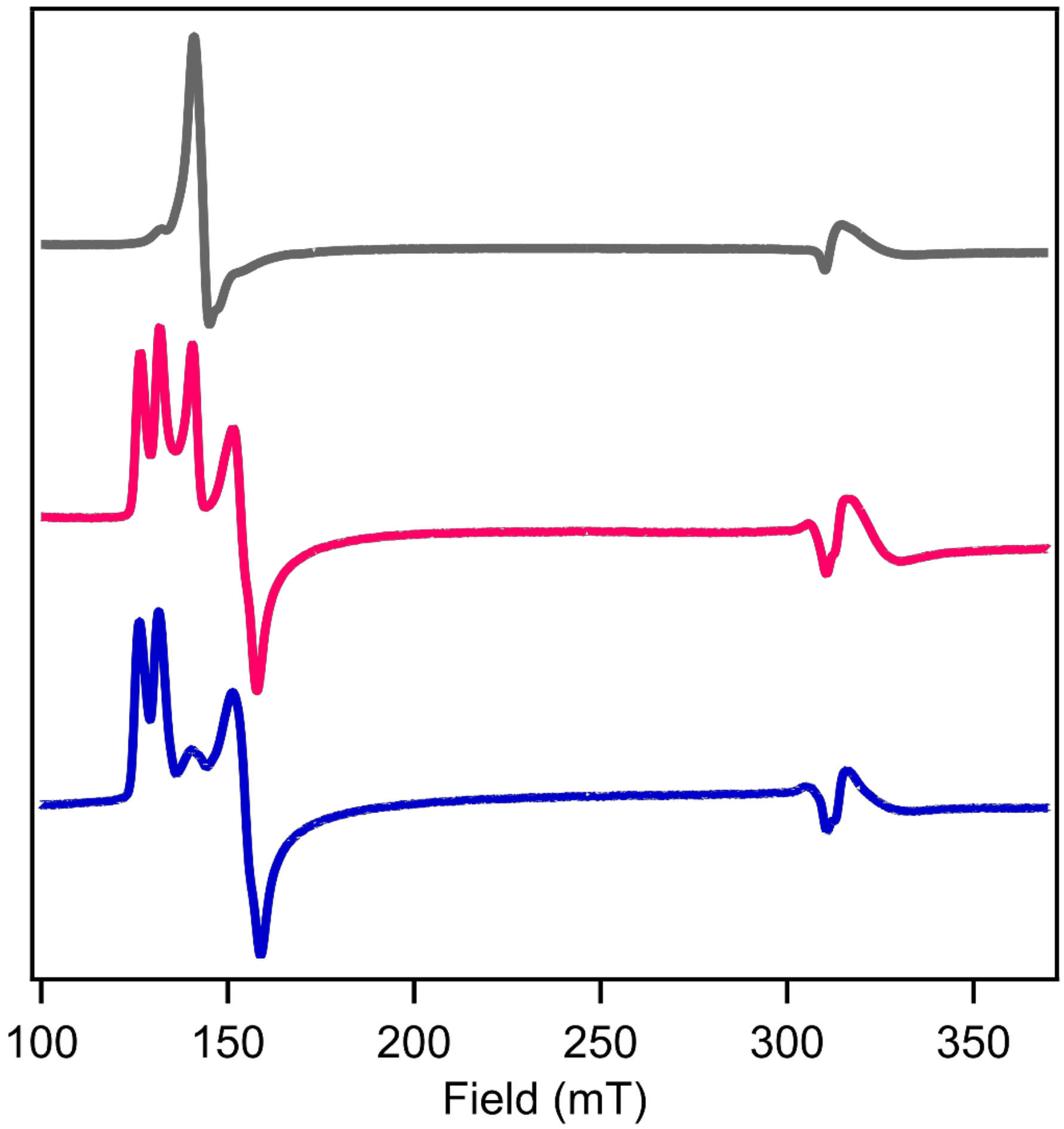

Figure S15. Continuous-wave, X-band EPR spectrum of the give the exact description of what is in there $\{Fe\text{-}NO\}^7$ control experiments: (*top*) without L-Lys/F^-, (*middle*) no L-Lys/100 mM F^-, and (*bottom*) 10 mM L-Lys/100 mM F^-. The data reveal that both L-Lys and fluoride are important for the complete formation of the fluoride bound BesD•$\{FeNO\}^7$•2OG•F^-•L-Lys species, corroborating the synergistic effect of substrate and halide in BesD. Spectra were collected at 12 K with 1 mT modulation amplitude, 20 dB microwave power, and 9.435179 GHz microwave frequency.

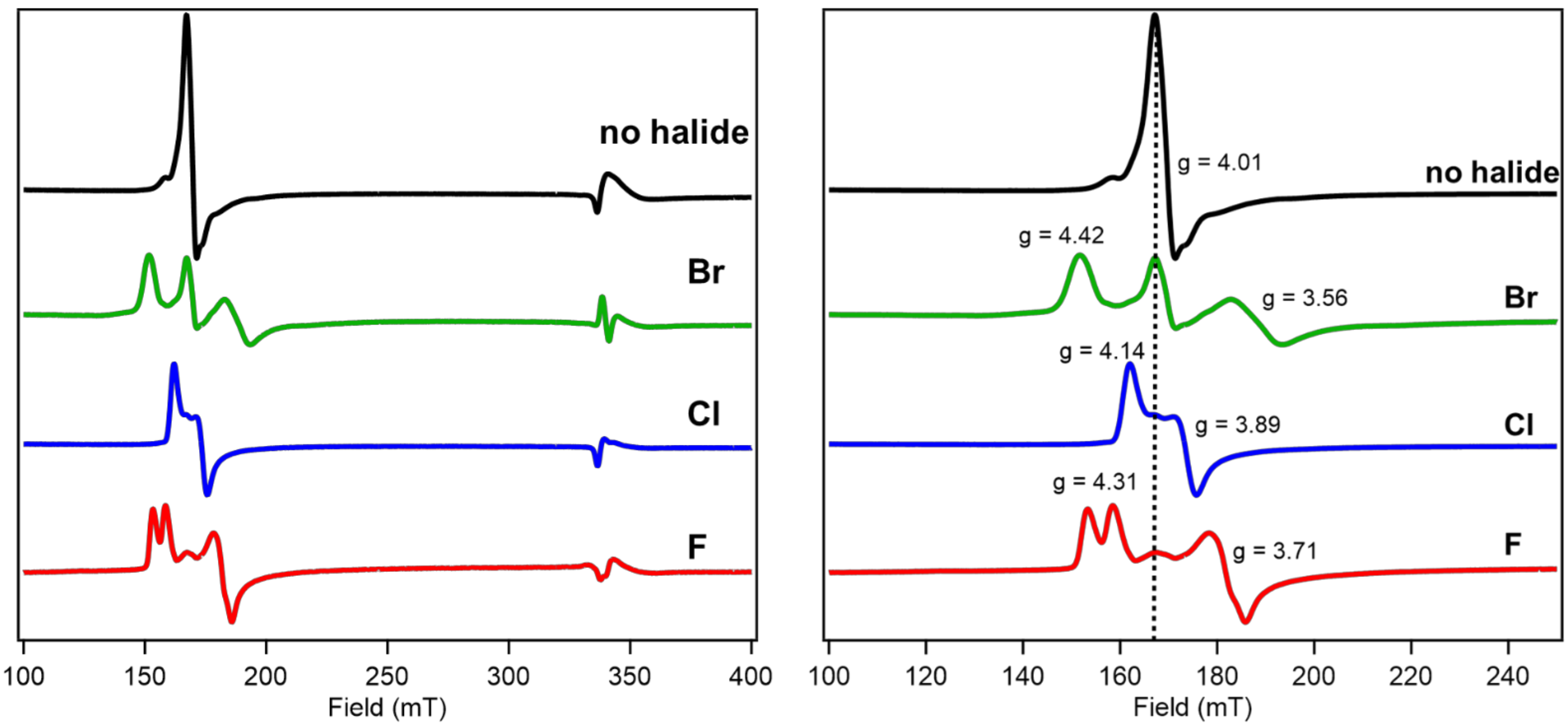

Figure S16. Continuous-wave, X-band EPR spectrum of the B BesD•{FeNO}[7]•2OG•X (X = F, Cl, Br)•L-Lys with different halides. The vertical dashed black line (*right*) indicates the relative amount of unreacted no halide bound species in each case. Based on the intensity of the peaks, the data reveal that the amount of complex lacking bound halide is greatest for the case of bromide and least for the case of chloride. Spectra were collected at 12 K with 1 mT modulation amplitude, 20 dB microwave power, and 9.435179 GHz microwave frequency.

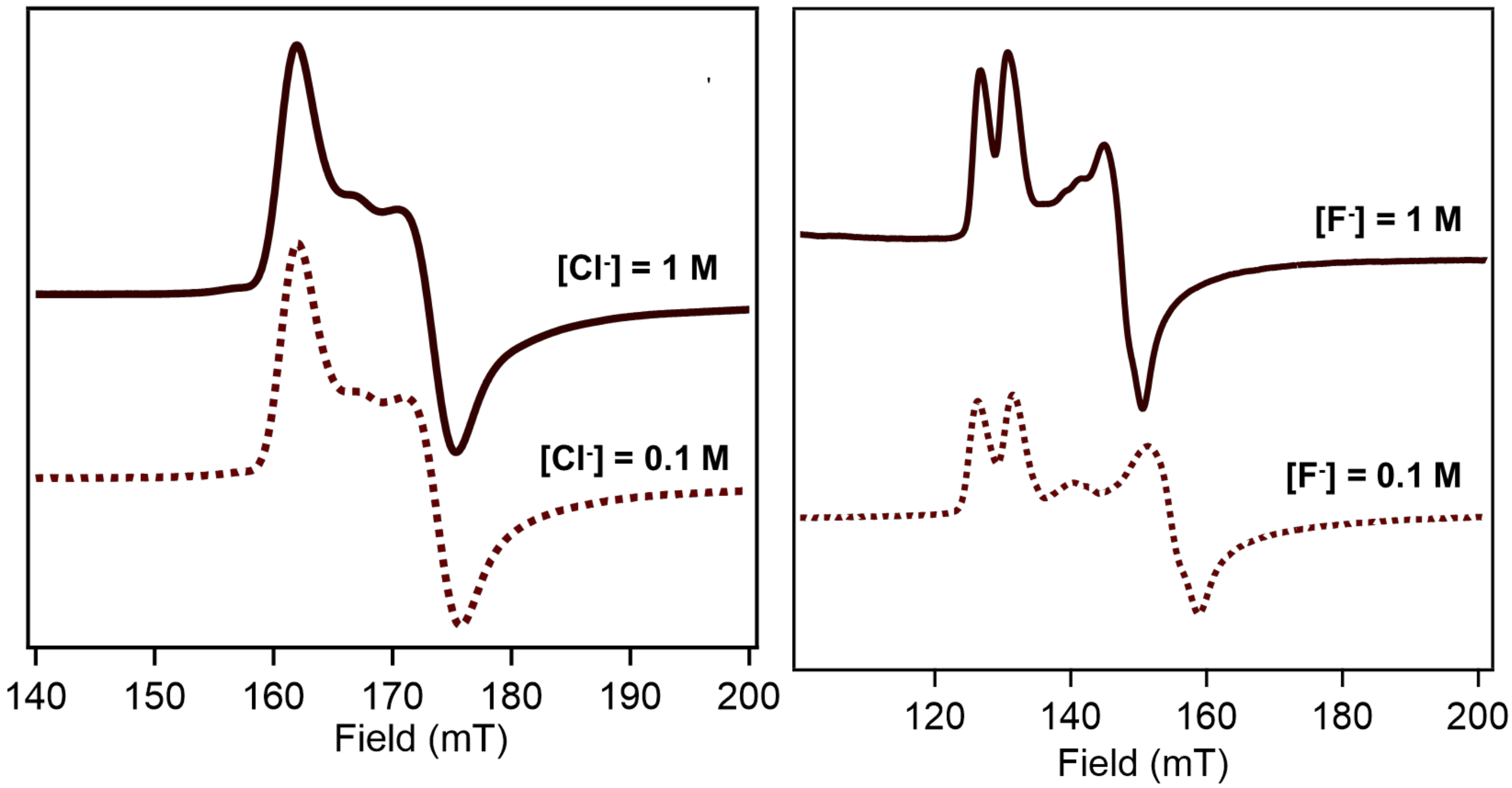

Figure S17. Continuous-wave, X-band EPR spectrum of the BesD•{FeNO}^{7}•2OG•Cl•L-Lys (*left*) and BesD•{FeNO}^{7}•2OG•F•L-Lys (*right*) at different concentrations. Spectra were collected at 12 K with 1 mT modulation amplitude, 20 dB microwave power, and 9.435179 GHz microwave frequency.

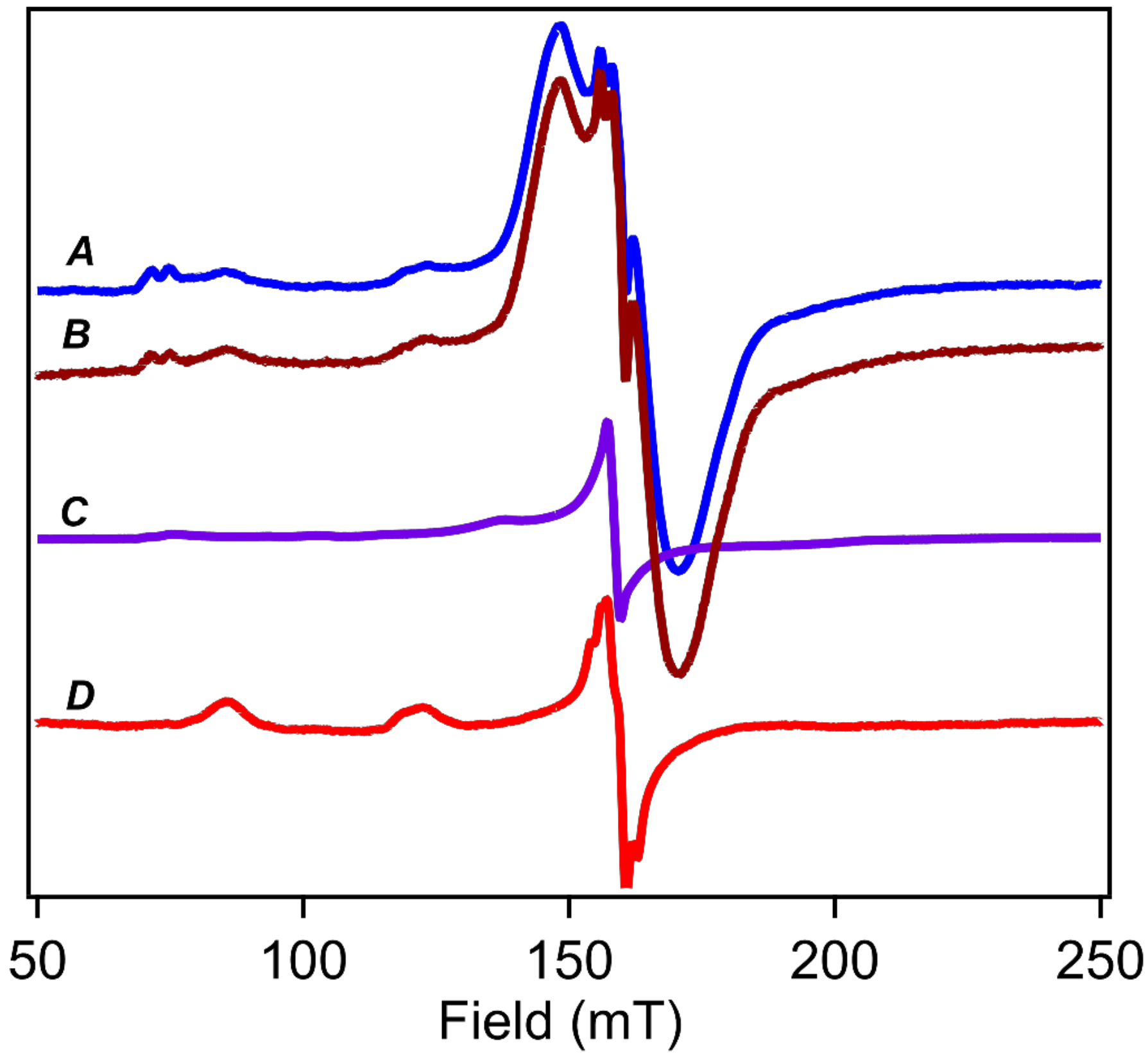

Figure S18. Continuous-wave, X-band EPR spectra of BesD-derived samples prepared by rapid mixing of an anoxic solution containing BesD•(Fe^{II}(F))•*d*$_4$-L-Lys•2OG with O_2-saturated 100 mM HEPES buffer (pH 7.5), followed by freeze-quenching at the indicated times. Panels **A** and **B** show spectra of the 8 s freeze-quenched sample recorded at 12 K and 8 K, respectively. Panels **C** and **D** show spectra collected from samples allowed to decay for 1000 s prior to freezing, prepared either without added azide (**C**) or in the presence of 50 equiv of azide (**D**). Spectra were recorded at 8 –12 K using a modulation amplitude of 1 mT, microwave power of 10 dB, and microwave frequency of 9.435954 GHz.

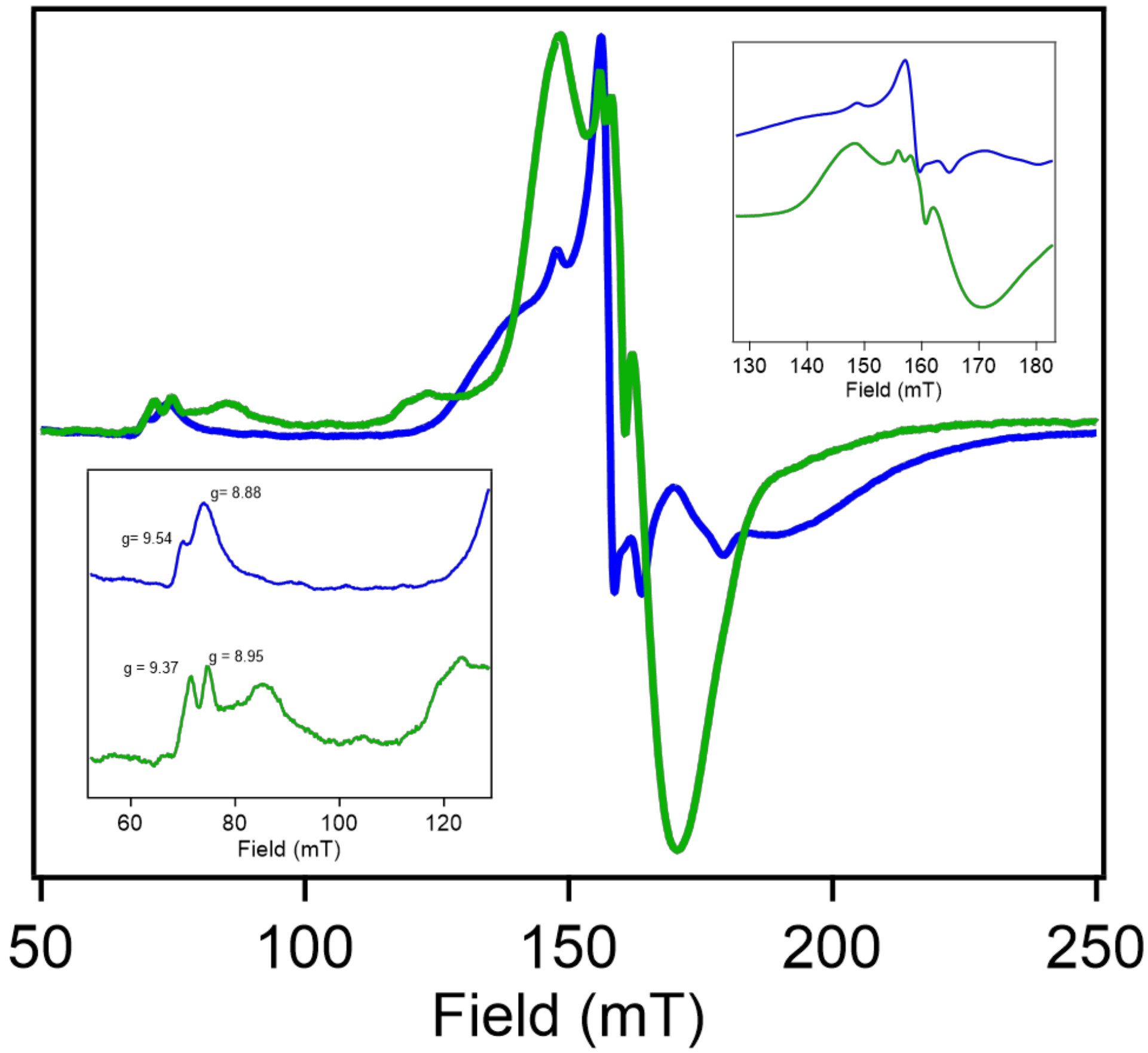

Figure S19. Continuous-wave, X-band EPR spectra recorded at 12 K of samples prepared by rapid mixing an anoxic solution containing BesD•(Fe^{II}(F))•*d*$_4$-L-Lys•2OG or BesD•(Fe^{II}(N_3))•*d*$_4$-L-Lys•2OG with O_2-saturated 100 mM HEPES buffer (pH 7.5), followed by incubation for 8 s and rapid-quenching adding 50 equiv azide followed by immersion liquid nitrogen. Spectra were collected using a microwave frequency of 9.435 GHz, microwave power of 10 dB, and modulation amplitude of 1 mT. The *green trace* corresponds to the sample generated from BesD•(Fe^{II}(F))•*d*$_4$-L-Lys•2OG and the *blue trace* to the sample generated from BesD•(Fe^{II}(N_3))•*d*$_4$-L-Lys•2OG.

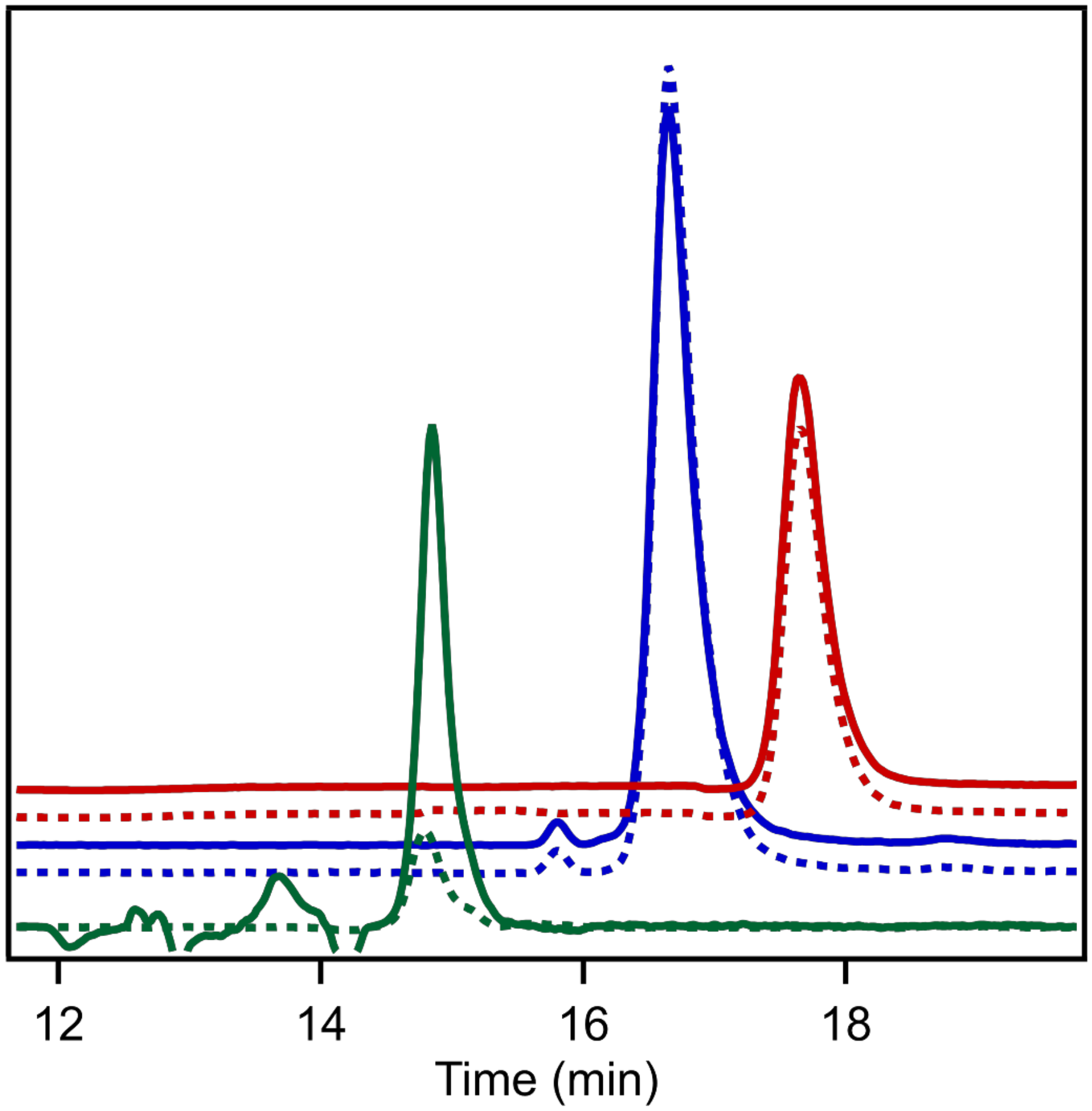

Figure S20. Single-ion LC-MS chromatograms monitoring 4-OH-L-Lysine (*m/z* 163.1, solid lines) and 4-^{18}OH-L-Lysine (*m/z* 165.1, dotted lines) in a reaction that initially contained containing 0.1 mM BesD, 0.1 mM Fe(II), 1 mM L-Lys, 200 mM CsF or NaCl or NaBr, 5 mM L-asc, 8 mM $Na_2$2OG, and 0.9 mM O_2 in 100 mM HEPES in $H_2{}^{18}O$ (50% v/v) at pH 7.5) (pH 7.5) that was incubated at 20 °C for 4 h (for all anions) before being quenched and analyzed as described in Liquid Chromatography Mass Spectrometry (LC-MS) section above**.**

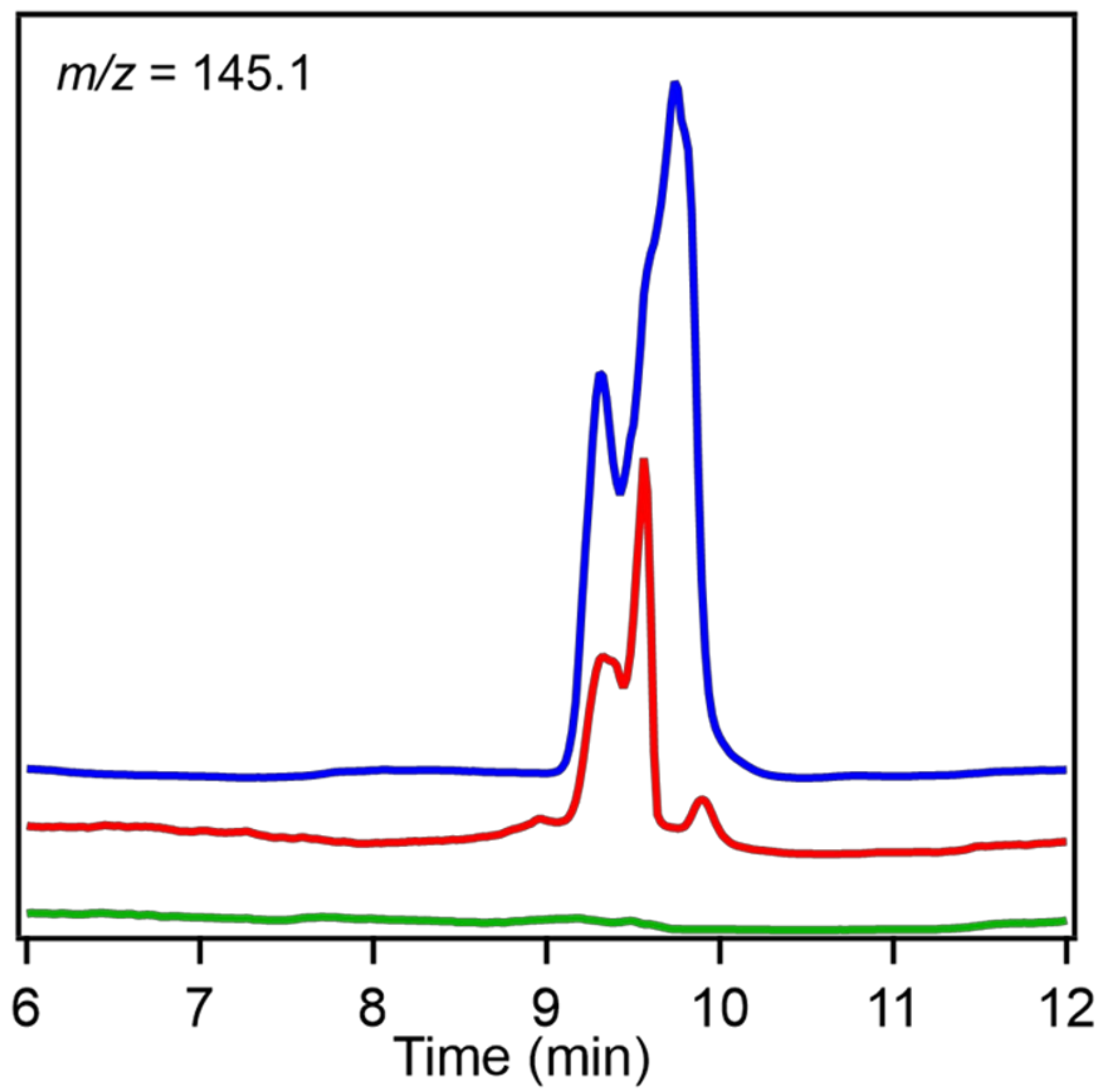

Figure S21. Single-ion LC-MS chromatograms monitoring the 2-amino-4-propylamine-γ-lactone/2-amino-4-hydroxy-ε-lactam products (*m/z* 145.1) in a reaction that initially contained containing 0.1 mM BesD, 0.1 mM Fe(II), 1 mM L-Lys, 200 mM CsF or NaCl or NaBr, 5 mM L-asc, 8 mM $Na_2$2OG, and 0.9 mM O_2 in 100 mM HEPES (pH 7.5) that was incubated at 20 °C for 4 h (for all anions) before being quenched and analyzed as described in Liquid Chromatography Mass Spectrometry (LC-MS) section above.

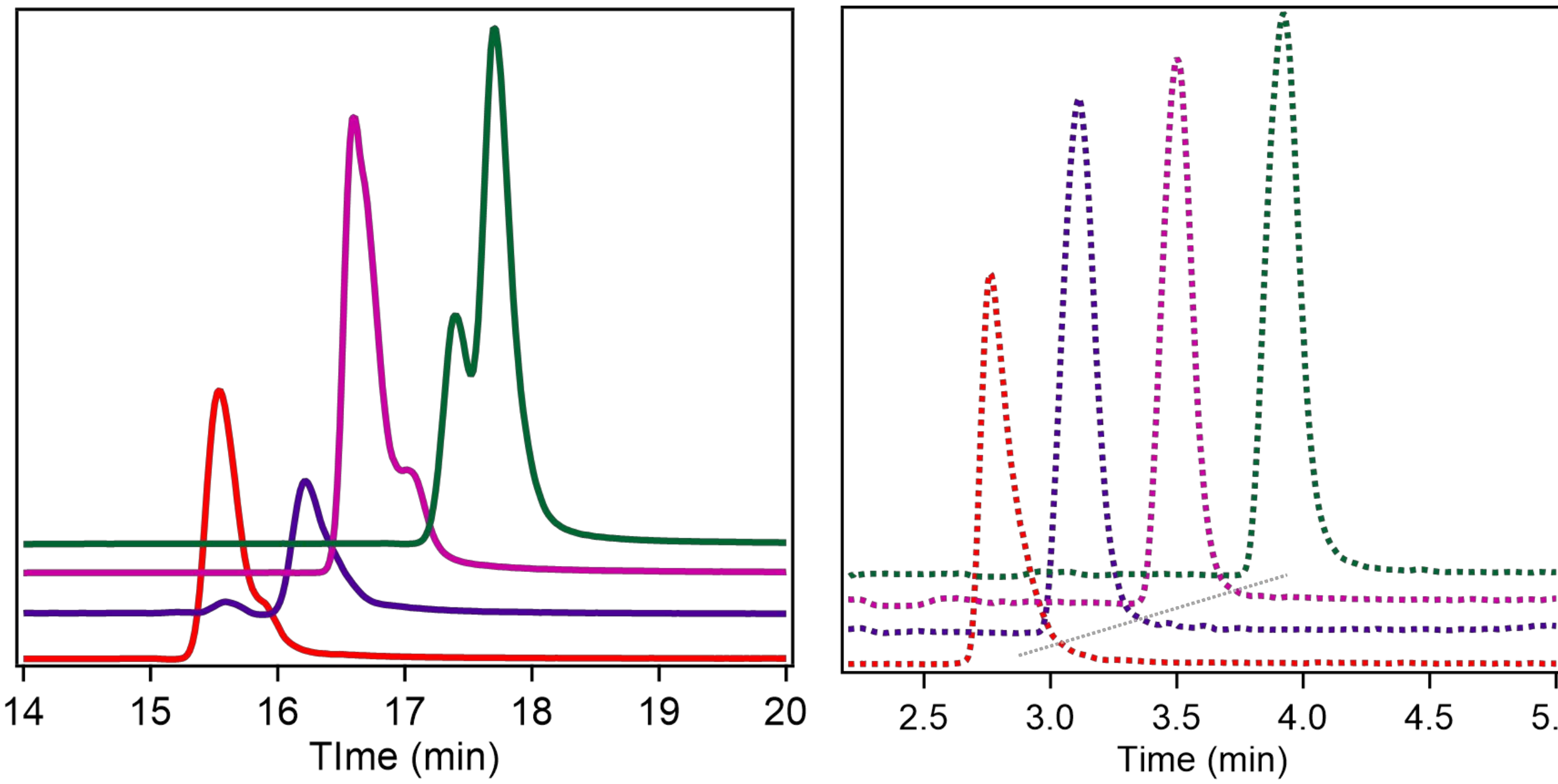

Figure S22. Single-ion LC-MS chromatograms monitoring (*left*) hydroxylated products (*m/z* 163.1 for 4-OH-L-Lysine, red solid trace; *m/z* 149.1 for OH-L-ornithine, purple solid trace, *m/z* 177.1 for OH-L-homolysine, magenta solid trace and 163.1 for OH-D-Lysine, green solid trace) (*right*) succinate (*m/z* = 117.1) co-products for each substrate (shown in the dotted lines) in a reaction that initially contained containing 0.1 mM BesD, 0.1 mM Fe(II), 5 mM substrate, 200 mM CsF, 5 mM L-asc, 8 mM $Na_2$2OG, and 0.9 mM O_2 in 100 mM HEPES (pH 7.5) that was incubated at 20 °C for 4 h before being quenched and analyzed as described in Liquid Chromatography Mass Spectrometry (LC-MS) section above.

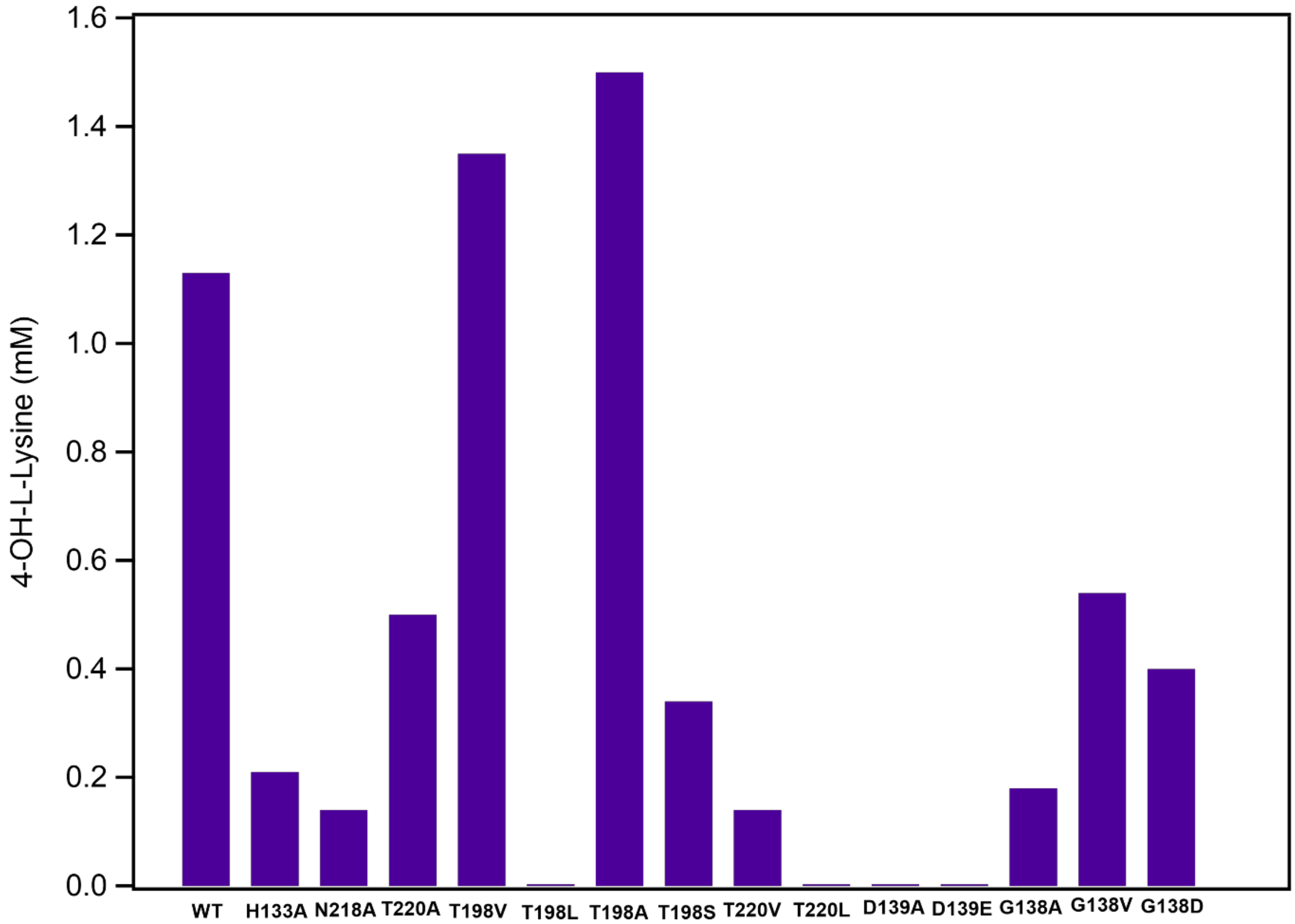

Figure S23. Relative yields of the 4-OH-L-Lysine (*m/z* 163.1) product from a reaction initially containing 0.5 mM BesD, 0.5 mM Fe(II), 5 mM L-Lys, 200 mM CsF, 5 mM L-asc, 8 mM $Na_2$2OG, and 0.9 mM O_2 in 100 mM HEPES buffer (pH 7.5) that was incubated at 20 °C for 4 h before being quenched and analyzed as described in Liquid Chromatography Mass Spectrometry (LC-MS) section above**.**

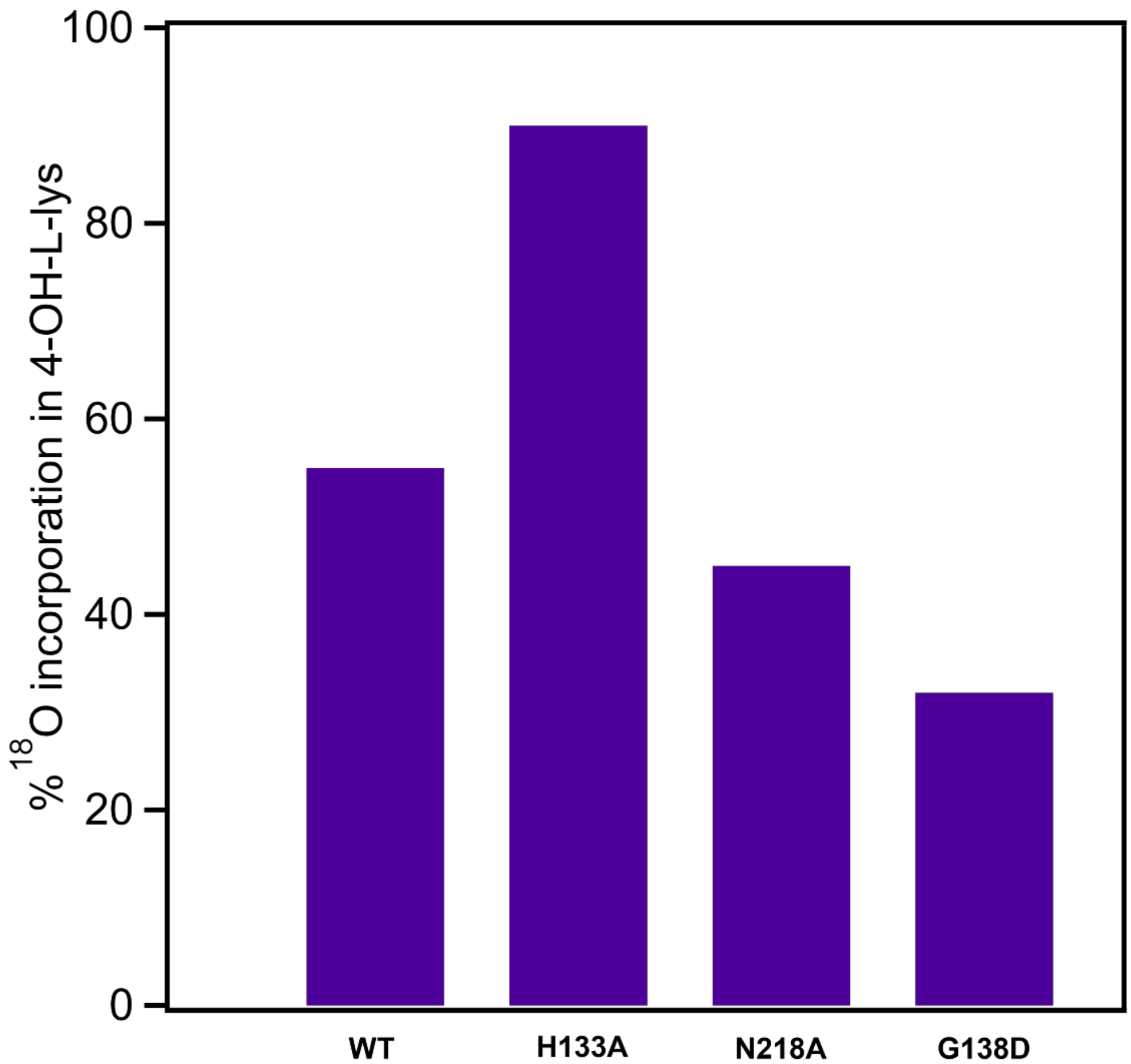

Figure S24. Relative %^{18}O incorporation in 4-^{18}OH-L-Lysine (*m/z* 165.1) product from a reaction initially containing 0.5 mM BesD, 0.5 mM Fe(II), 5 mM L-Lys, 200 mM CsF (specified anion), 5 mM L-asc, 8 mM $Na_2$2OG, and $^{18}O_2$ saturated buffer in 100 mM HEPES buffer (pH 7.5) that was incubated at 20 °C for 4 h before being quenched and analyzed as described in Liquid Chromatography Mass Spectrometry (LC-MS) section above**.**

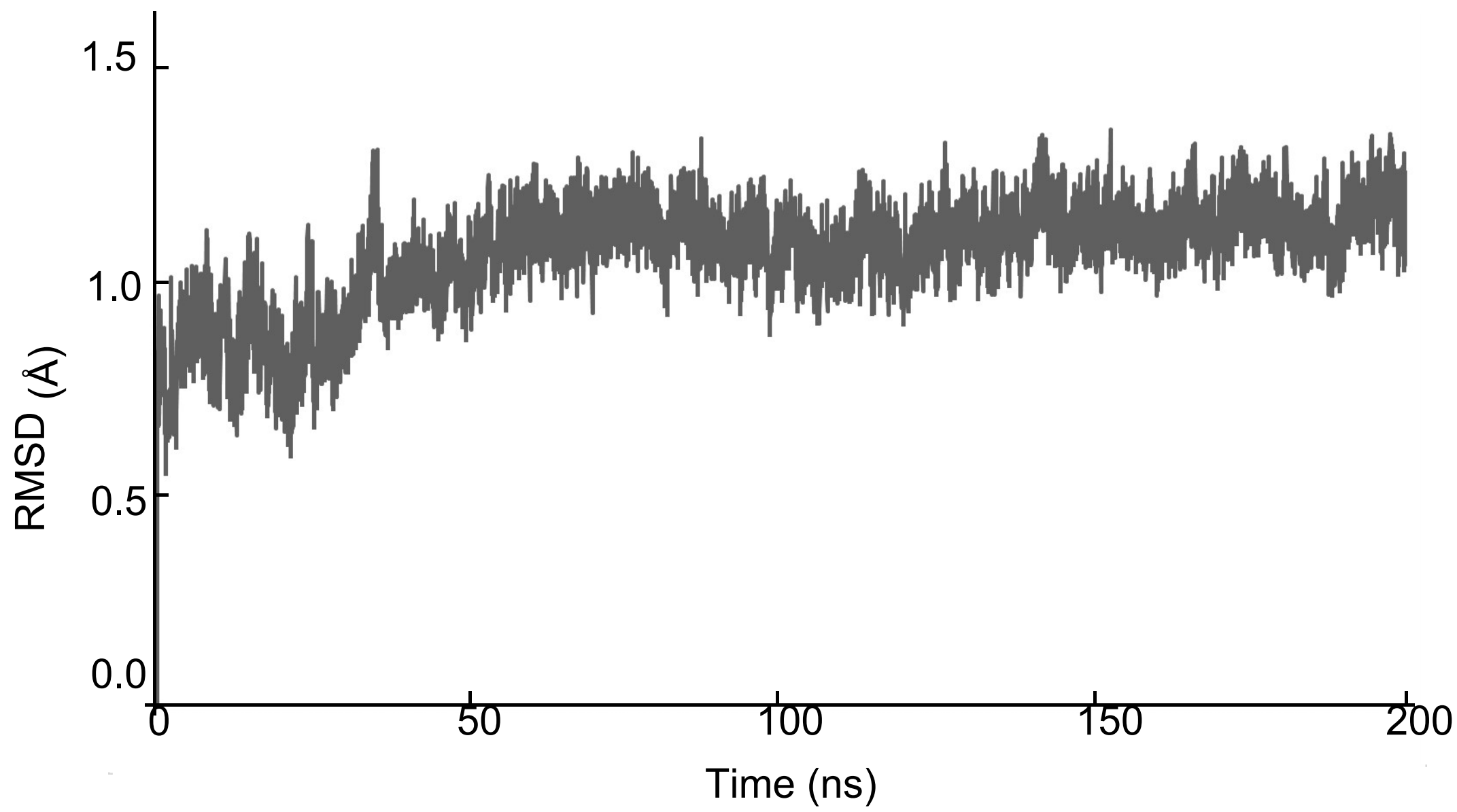

Figure S25. Time evolution of the root mean square deviation (RMSD) of the protein backbone for BesD in the Fe(III)–O_2^- (superoxo) state during molecular dynamics simulations. RMSD values were calculated relative to the starting structure using backbone atoms (N, Cα, C) to monitor global conformational stability over the course of the simulation. This analysis was performed to assess whether formation of the Fe(III)–O_2^- intermediate induces significant structural rearrangements in the protein scaffold or whether the enzyme maintains a stable fold while accommodating the reactive oxygen species. The RMSD profile that the BesD backbone remains structurally stable in the presence of the superoxo intermediate, thereby supporting the interpretation that observed changes in reactivity arise from local active-site dynamics rather than global protein unfolding.

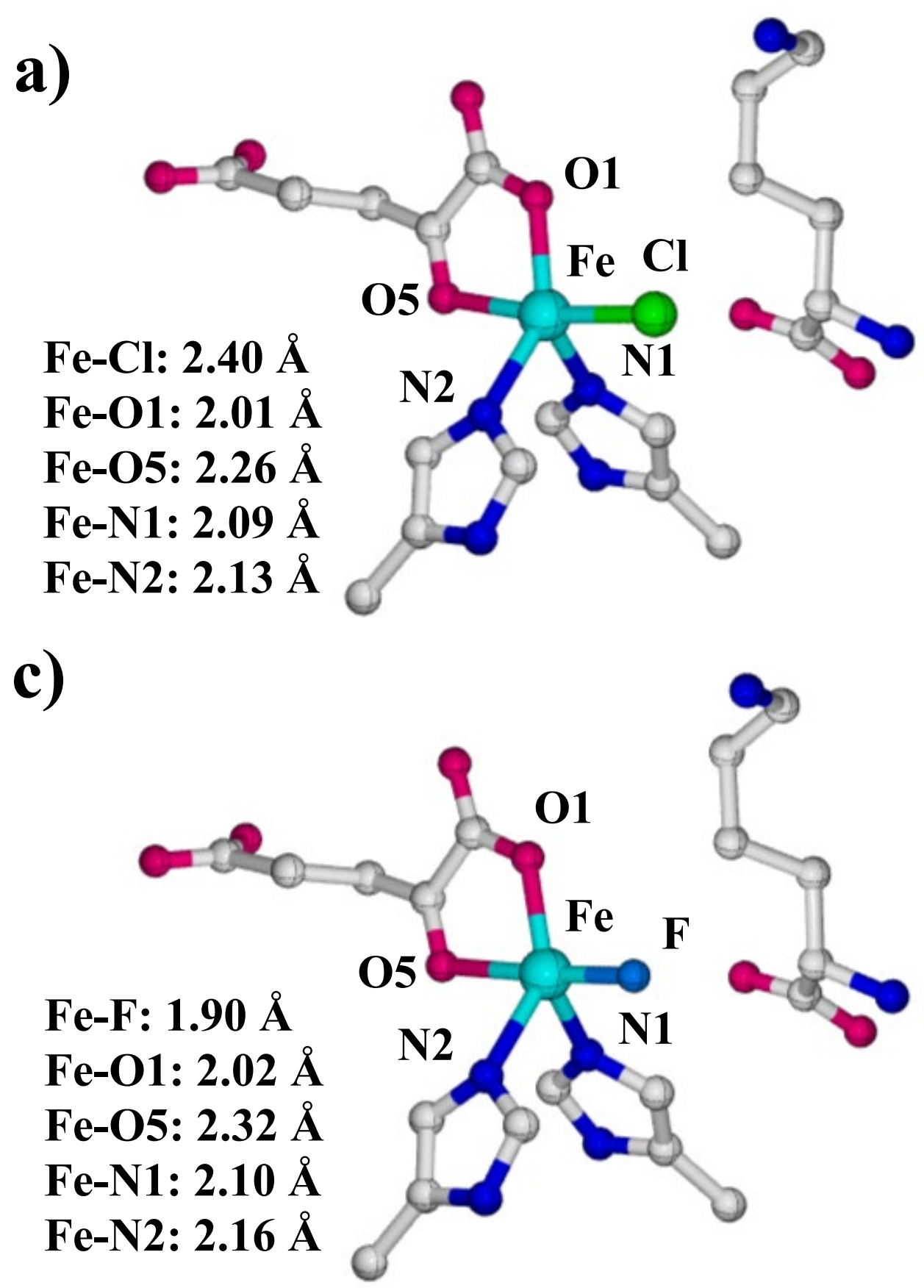

Figure S26. Geometries and key bond lengths of the structures in **Figure 7**. All non-essential hydrogen atoms and the surrounding protein environment have been omitted for clarity in **Figures S26-30**. The following color scheme was used: cyan for iron, green for chlorine, brown for bromine, blue for fluorine, dark blue for nitrogen, magenta for oxygen, gray for carbon, and yellow for hydrogen. Red dots represent radical electrons.

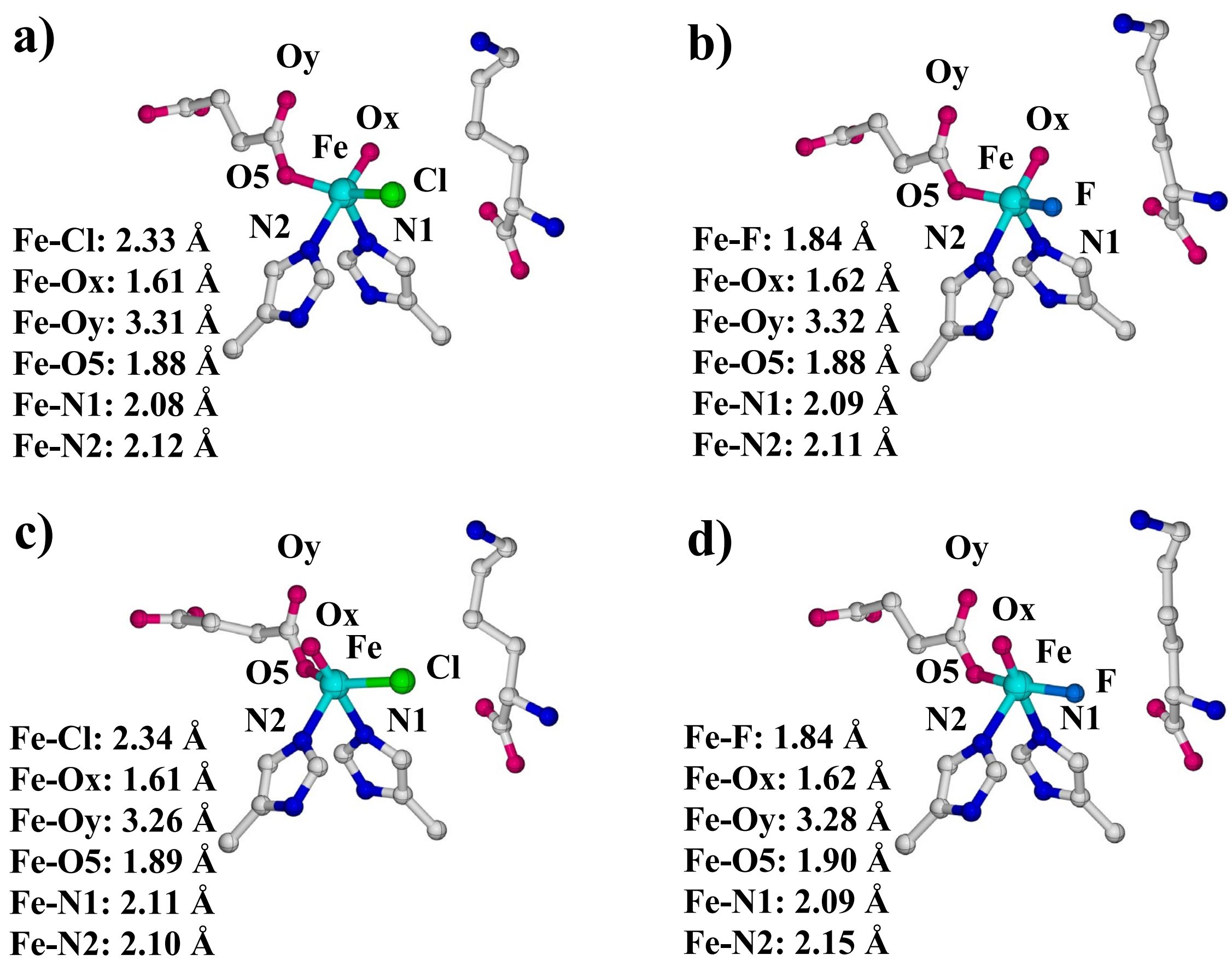

Figure S27. Geometries and key bond lengths of the structure in **Figure 8**.

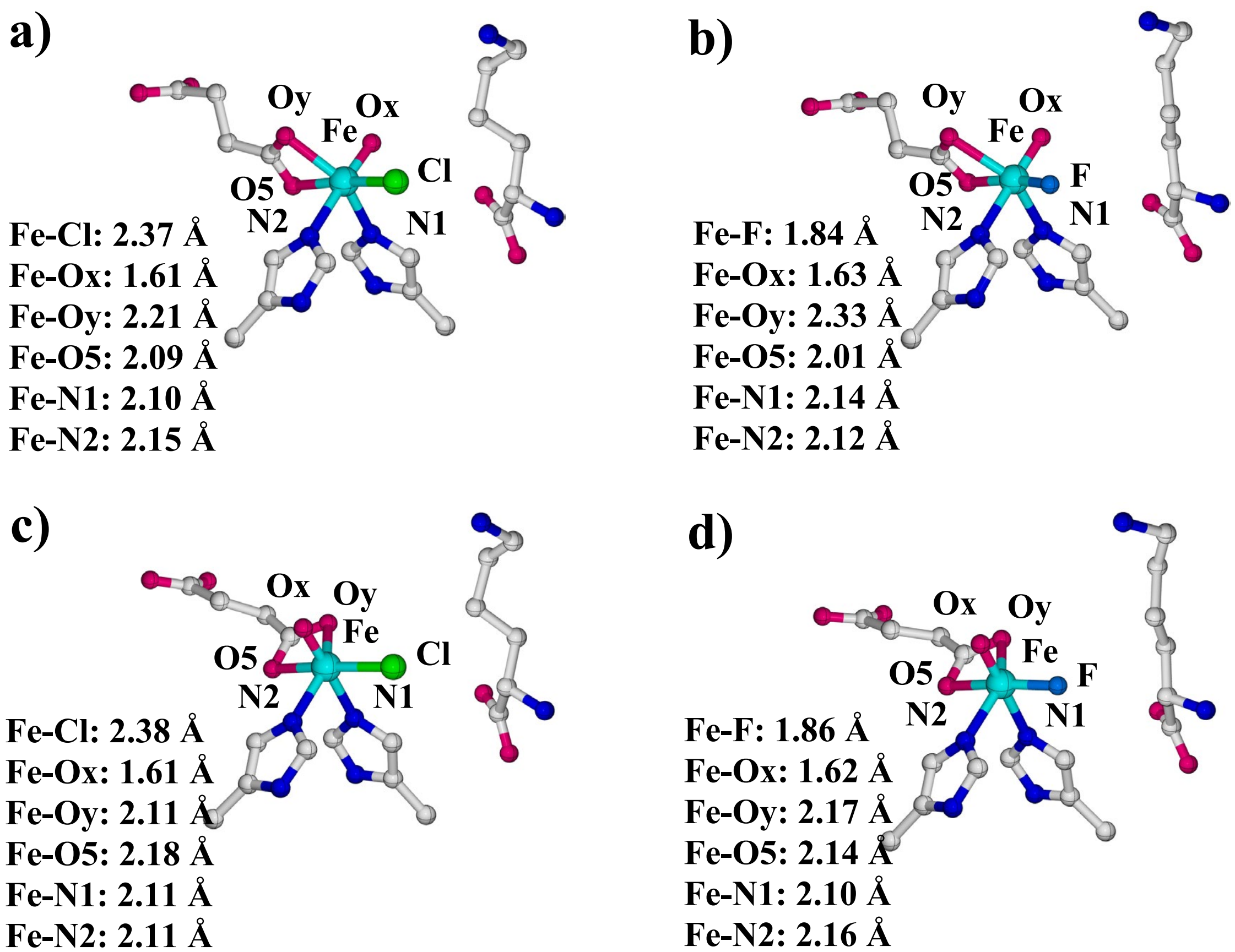

Figure S28. Geometries and key bond lengths of the bidentate succinate structures.

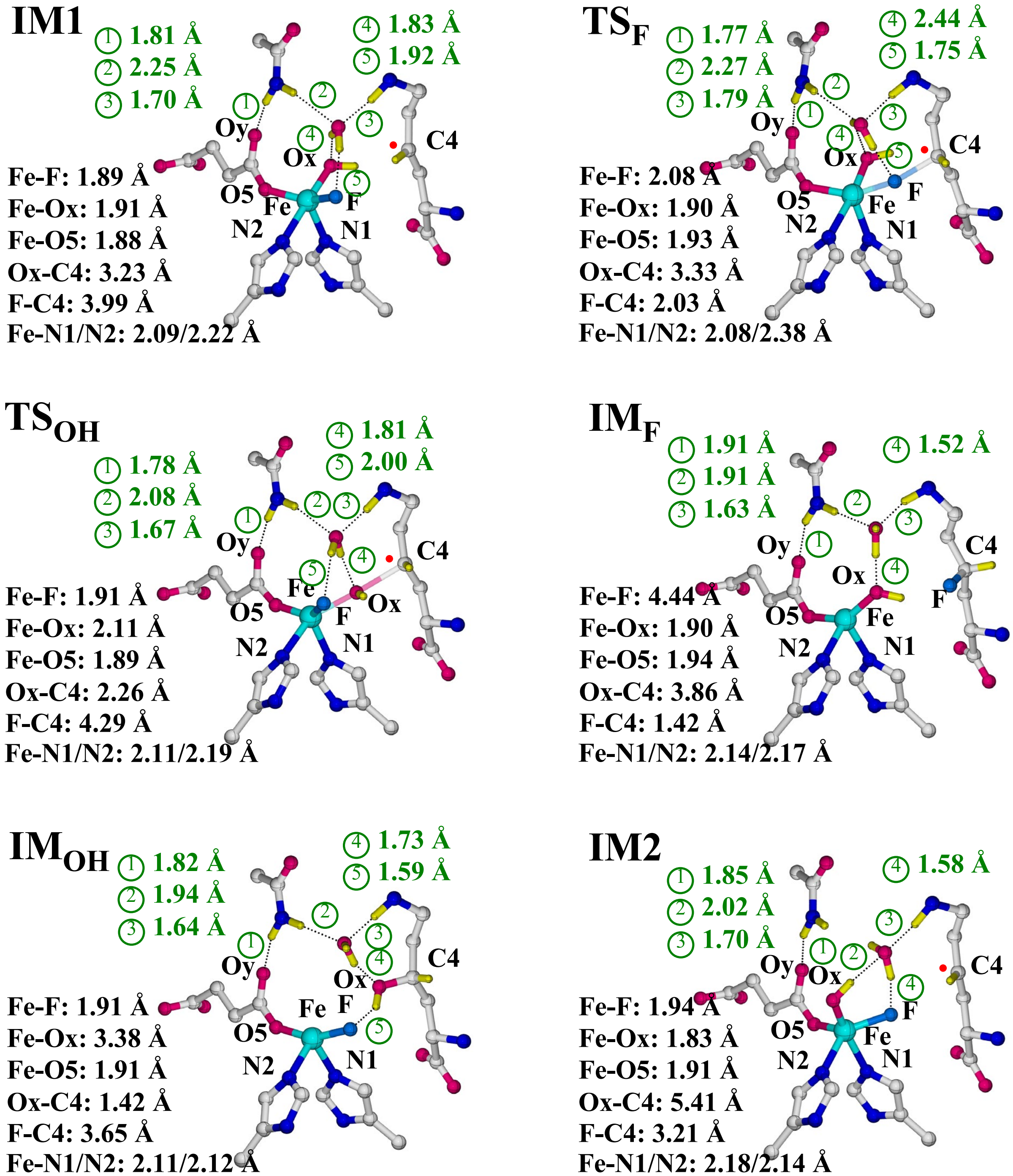

Figure S29a. Geometries and bond lengths of the structures in **Figure 11**, all in the pro-*R* conformation.

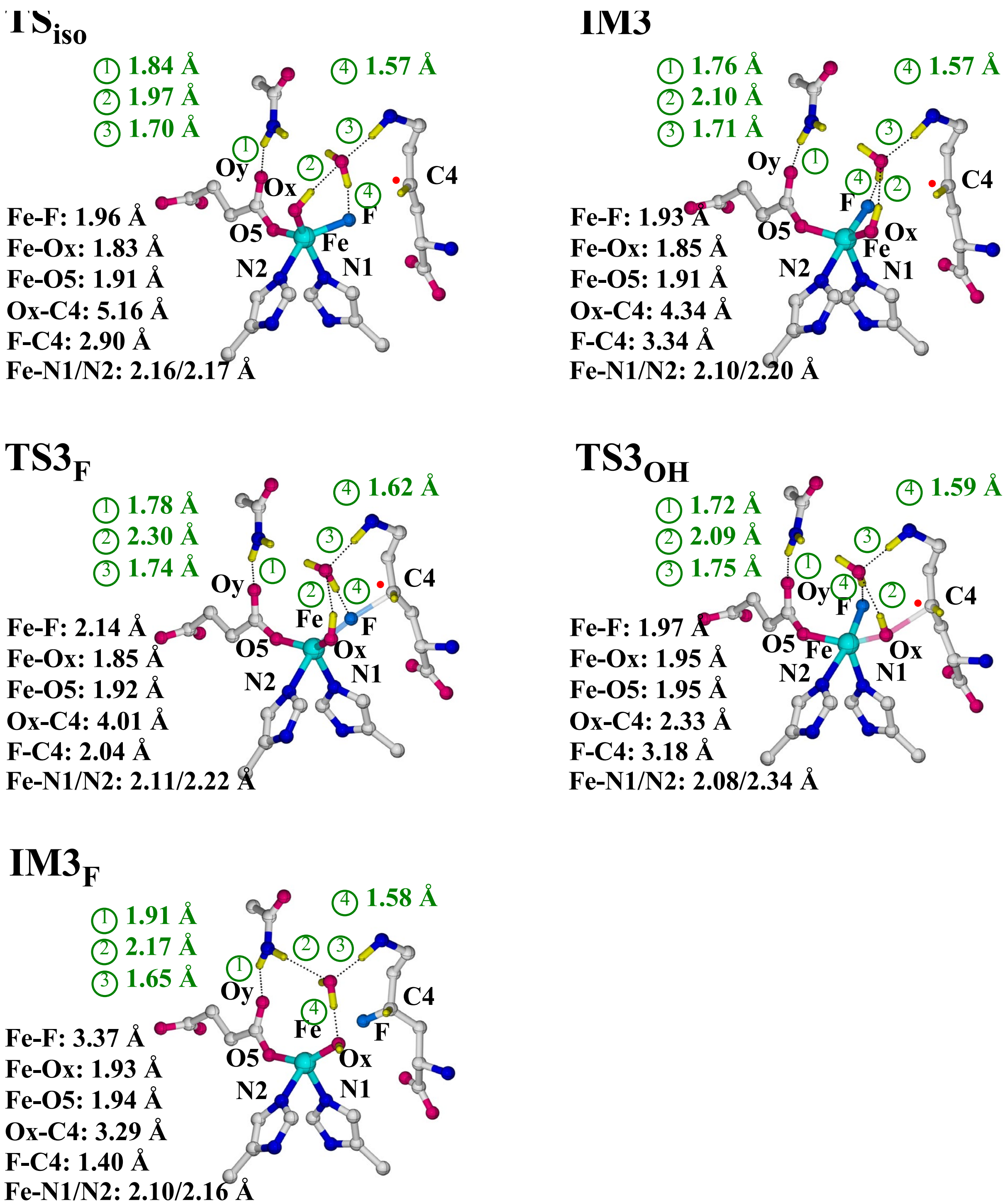

Figure S29b. Geometries and bond lengths of the structures in **Figure 11**, all in the pro-*R* conformation.

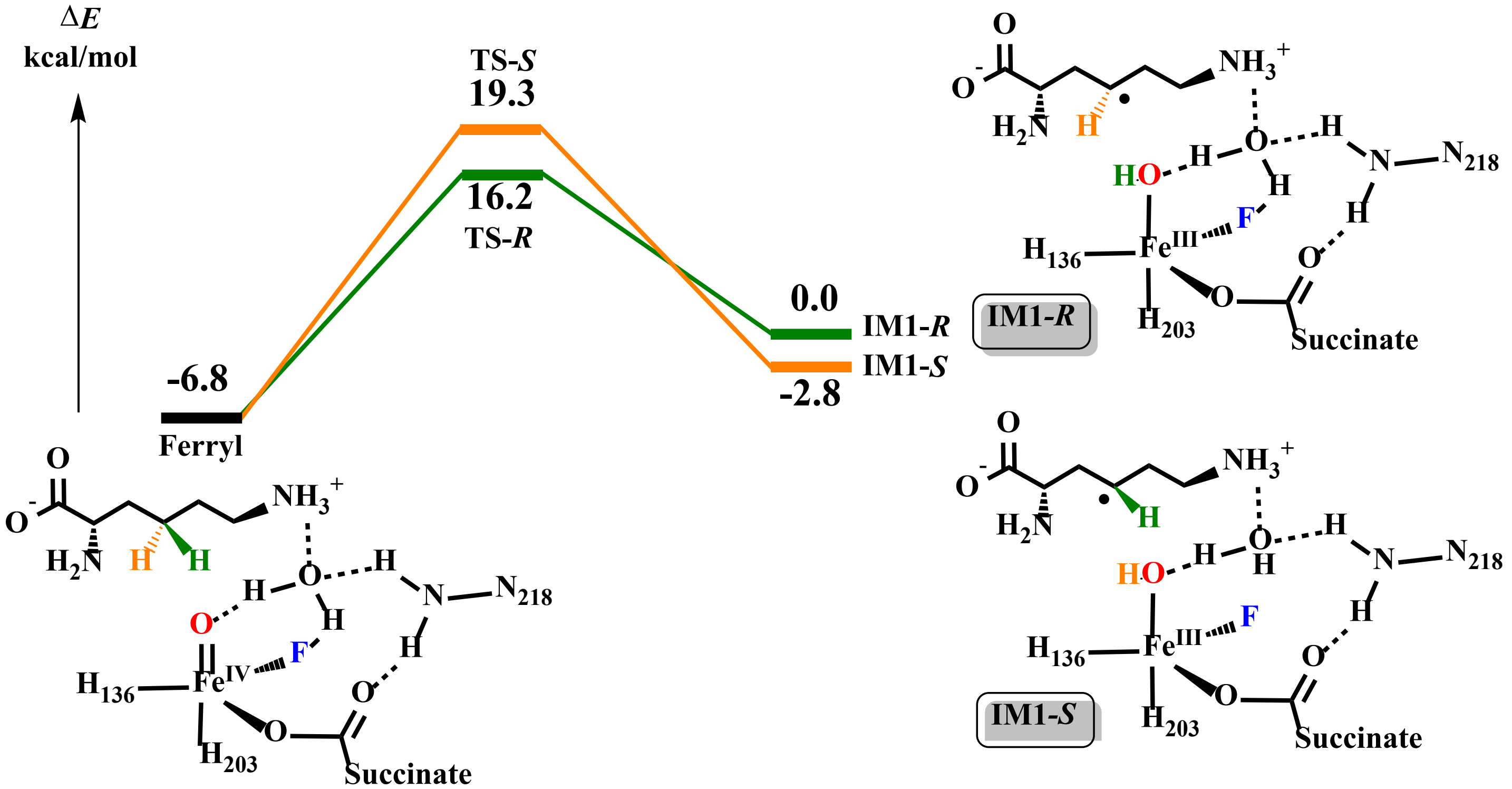

Figure S30. Reaction profile for hydrogen atom transfer (HAT) in the fluoroferryl complex. Abstraction from the L-Lys substrate can occur at either hydrogen, leading to IM-R or IM-S intermediates. The difference in calculated energy barriers suggests a stereoselective preference during the HAT step.

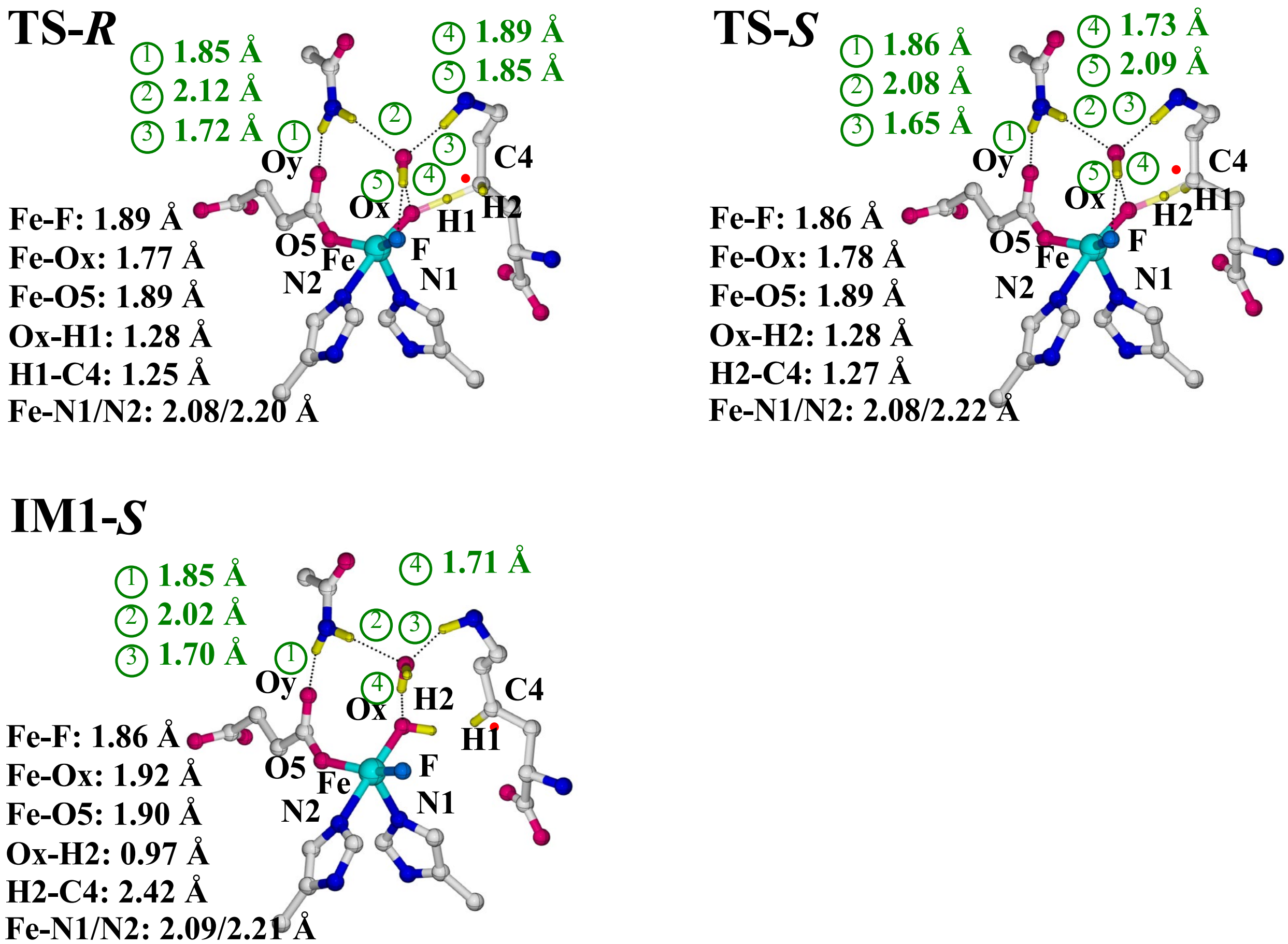

Figure S31. Geometries and bond lengths of the structures in **Figure S30**.

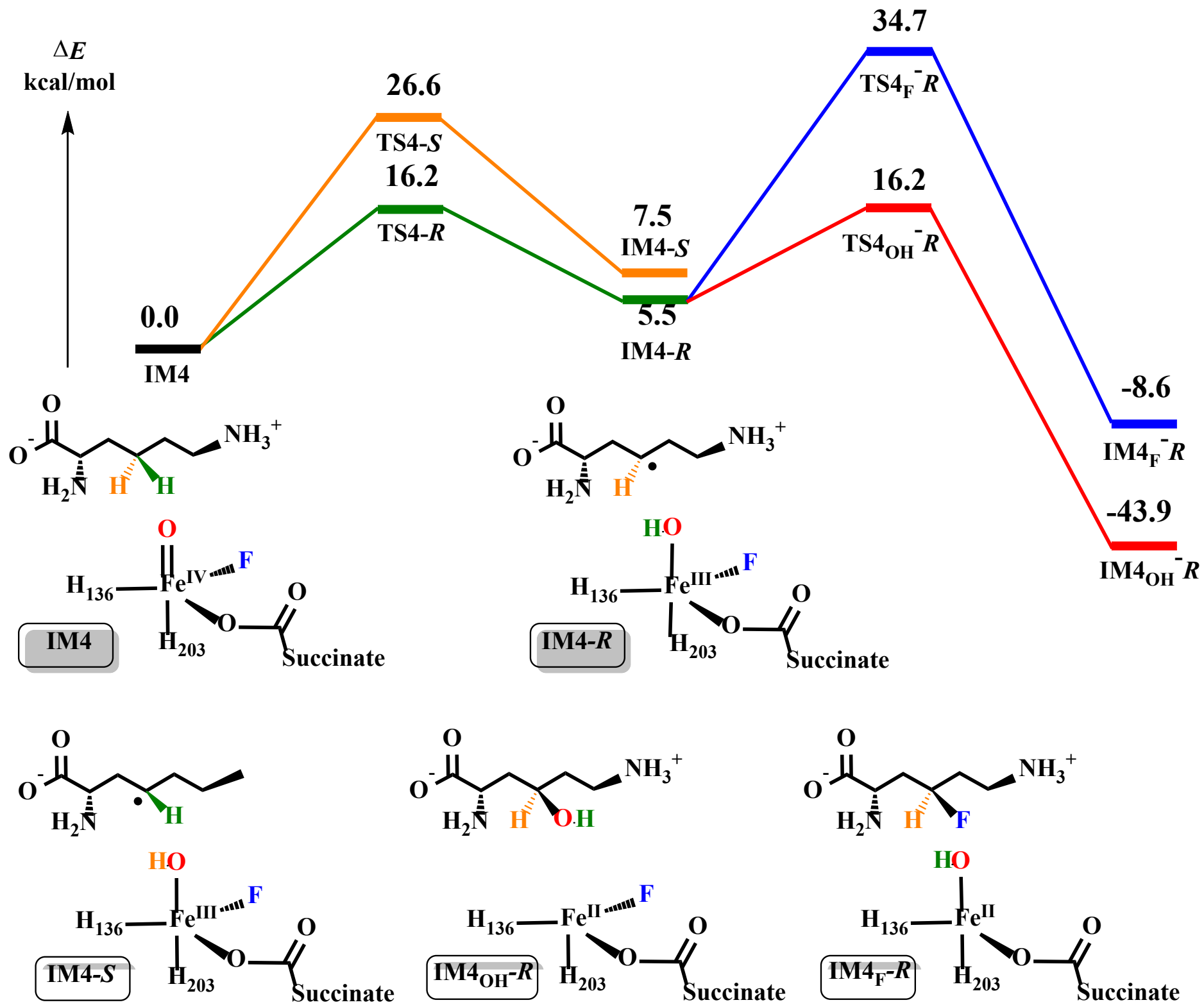

Figure S32. Reaction profiles for both HAT and the radical rebound steps from QM/MM simulations starting with the fluoroferryl species using a smaller QM region that excludes hydrogen-bonding interactions with a water molecule and Asn218. In this smaller model, only the first coordination ligands of Fe and the prime substrate L-Lys were included. Our results indicate that the *R* configuration has a lower reaction barrier and that OH radical rebound is energetically more favorable, in agreement with the findings from the simulations with a larger QM region.

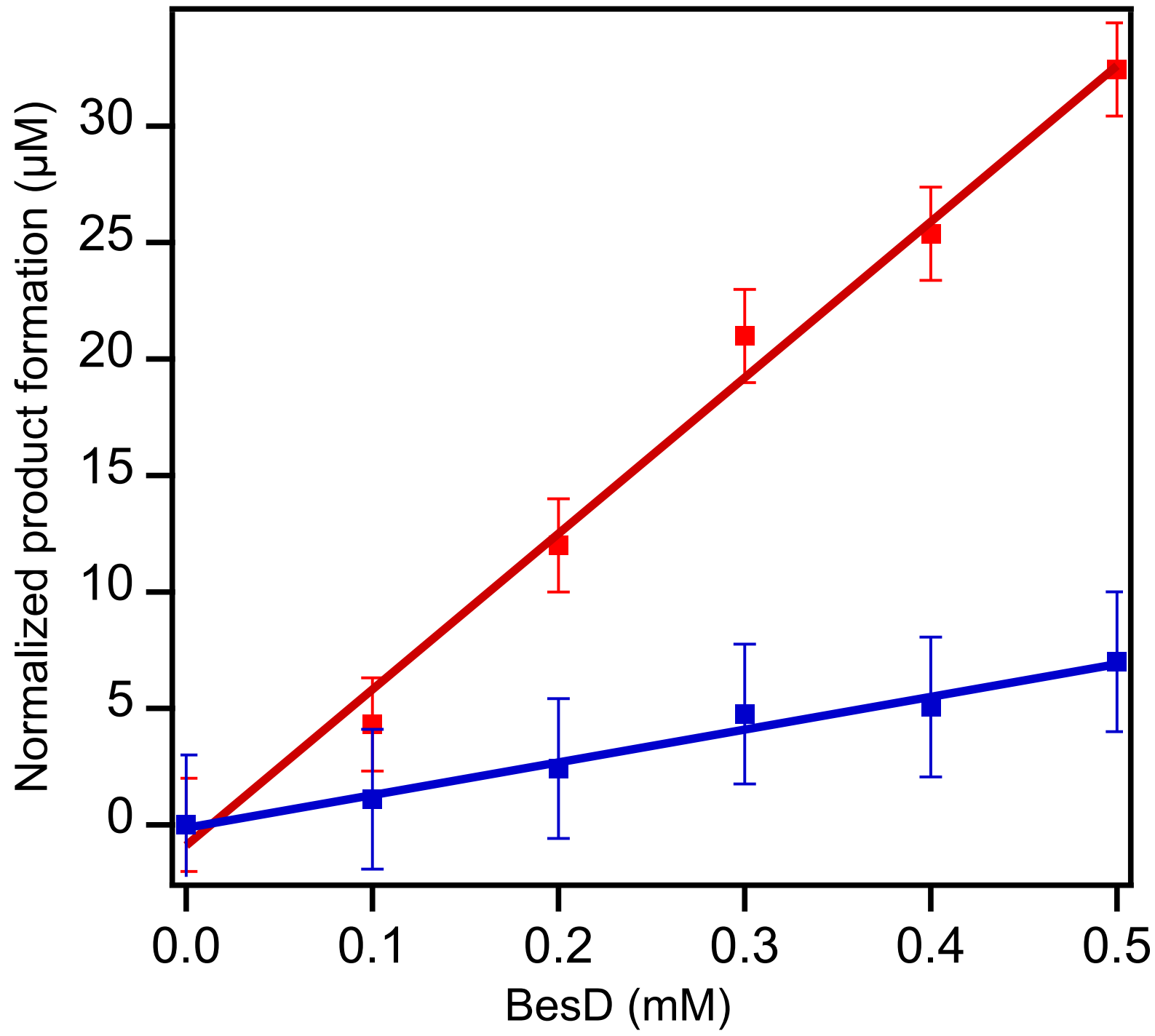

Figure S33. Equivalents of C4-hydroxylated products formed (blue circles) in reactions containing L-Lys (red squares) or d_4-L-Lys (blue squares) as a function of enzyme concentration. Reactions initially contained BesD•Fe(II)•2OG•F^-•L-Lys (or d_4-L-Lys) and were initiated by addition of O_2-saturated buffer (100 mM HEPES, pH 7.5) at 20 °C. Final reaction conditions were [BesD] = 0.7 mM, [Fe(II)] = 0.5 mM, [L-Lys or d_4-L-Lys] = 1 mM, [F^-] = 200 mM, [Na_2-2OG] = 10 mM, and [O_2] = 1.8 mM. Reactions were incubated for 4 h prior to quenching and analysis as described in the Liquid Chromatography Mass Spectrometry (LC-MS) section above**.**

Supporting Tables

Table S1. Computed Mössbauer parameters for structures in **Figure S26**

Structures	δ (mm/s)	ΔE_Q (mm/s)
**a)**	1.06	2.94
**b)**	1.06	2.74
**c)**	1.06	2.66

Table S2. Computed Mössbauer parameters for structures in Figure S27.

Structures	δ (mm/s)	ΔE_Q (mm/s)
**a)**	0.21	-1.00
**b)**	0.17	-0.87
**c)**	0.22	-1.34
**d)**	0.18	-1.37

Table S3. Computed Mössbauer parameters for structures in Figure S28.

Structures	δ (mm/s)	ΔE_Q (mm/s)
**a)**	0.30	-0.84
**b)**	0.24	-0.80
**c)**	0.30	-1.08
**d)**	0.28	-0.74

Table S4. QM/MM Electronic Energies (a.u.) at B3LYP-GD4/def2-TZVP level of theory.

Structures	def2-TZVP
**IM1**	-3604.852771
**TS_F**	-3604.792925
**TS_{OH}**	-3604.830036
**IM_F**	-3604.904544
**IM_{OH}**	-3604.922200
**IM2**	-3604.846898

**TS_{iso}**	-3604.821862
**IM3**	-3604.849927
**$TS3_F$**	-3604.816453
**$TS3_{OH}$**	-3604.806635
**$IM3_F$**	-3604.891142
**Ferryl**	-3604.863621
**TS-*R***	-3604.826908
**TS-*S***	-3604.822085
**IM1-*S***	-3604.857243

Table S5. Spin populations for key atoms in all structures in Figure 7, 8, 11 at B3LYP-GD4/def2-SVP level of theory.

Species	Fe	Ox	Oy	Cl/Br/F	O5	C4-lys
**Figure 7a**	3.71	N/A	N/A	0.07	0.05	0.00
**Figure 7b**	3.70	N/A	N/A	0.07	0.05	0.00
**Figure 7c**	3.69	N/A	N/A	0.12	0.04	0.00
**Figure 8a**	3.04	0.67	0.02	0.08	0.11	0.00
**Figure 8b**	3.08	0.59	0.02	0.11	0.09	0.00
**Figure 8c**	3.02	0.73	0.02	0.08	0.08	0.00
**Figure 8d**	3.08	0.64	0.02	0.09	0.06	0.00
**Figure 8a-bi**	3.07	0.62	0.08	0.08	0.09	0.00
**Figure 8b-bi**	3.12	0.54	0.09	0.10	0.05	0.00
**Figure 8c-bi**	3.04	0.67	0.07	0.09	0.09	0.00
**Figure 8d-bi**	3.09	0.58	0.09	0.12	0.07	0.00
**IM1**	4.10	0.24	0.02	0.18	0.21	-0.92
**TS_F**	3.91	0.23	0.02	0.07	0.15	-0.49
**TS_{OH}**	3.95	0.18	0.01	0.15	0.15	-0.58
**IM_F**	3.72	0.10	0.01	0.00	0.07	0.00
**IM_{OH}**	3.72	0.00	0.01	0.09	0.07	0.00
**IM2**	4.07	0.38	0.02	0.15	0.18	-0.93
**TS_{iso}**	4.08	0.38	0.02	0.14	0.18	-0.92
**IM3**	4.08	0.35	0.02	0.16	0.17	-0.93
**$TS3_F$**	3.95	0.26	0.01	0.07	0.12	-0.55
**$TS3_{OH}$**	3.95	0.25	0.01	0.12	0.13	-0.57
**$IM3_F$**	3.72	0.09	0.01	0.00	0.06	0.00
**TS-*R***	3.88	-0.04	0.01	0.17	0.15	-0.28
**TS-*S***	3.87	-0.03	0.02	0.18	0.15	-0.30
**IM1-*S***	4.10	0.22	0.02	0.22	0.20	-0.92

Table S6. Primers used for site-directed mutagenesis. Bolded nucleotides indicate the variant codon, and italics show the BsaI cut site. Primers were purchased from Integrated DNA Technologies (Coralville, IA).

Site	Direction	Sequence
H133	Reverse	5'-*GGAGGTCTC*ACGTGTCCCCGCTCCG-3'
H133A	Forward	5'-*GGAGGTCTC*ACACG**GCG**GGCTGGCACTGGGGC-3'
G138	Reverse	5'-*GGAGGTCTC*ACCAGTGCCAGCCGTG-3'
G138A	Forward	5'-*GGAGGTCTC*ACTGG**GCG**GATTTCAGCTTCGCCCTCATC-3'
G138V	Forward	5'-*GGAGGTCTC*ACTGG**GTG**GATTTCAGCTTCGCCCTCATC-3'
T198	Reverse	5'-*GGAGGTCTC*AGCGCAGGAAATACACGTCG-3'
T198A	Forward	5'-*GGAGGTCTC*AGCGC**GCG**GACACCACGCTCCACC-3'
T198L	Forward	5'-*GGAGGTCTC*AGCGC**CTG**GACACCACGCTCCACC-3'
N218	Reverse	5'-*GGAGGTCTC*ACAGGATGATGCGGGTGG-3'
N218A	Forward	5'-*GGAGGTCTC*ACCTG**GCG**ATGACCTGGGCGGG-3'
T220	Reverse	5'-*GGAGGTCTC*ACATGTTCAGGATGATGCGG-3'
T220A	Forward	5'-*GGAGGTCTC*ACATG**GCG**TGGGCGGGCGAG-3'

Cartesian Coordinates of the Optimized Structures

Figure 7a

C	25.95353	38.76404	41.82946
C	27.39352	38.38785	41.94324
N	27.85695	37.30068	42.64823
C	28.51442	38.97455	41.41032
C	29.19677	37.25477	42.53360
N	29.62569	38.26916	41.79771
H	25.91578	39.84910	41.64856
H	25.42278	38.58958	42.77551
H	27.26923	36.65014	43.17847
H	28.60183	39.83647	40.75615
H	29.82701	36.49434	42.98807
C	29.49628	33.81994	38.56457
C	29.99006	35.21806	38.75757
N	29.70083	36.27270	37.90862
C	30.80513	35.75364	39.72790
C	30.31507	37.37731	38.38136
N	30.99754	37.09477	39.48110
H	30.13564	33.13330	39.14216
H	29.59192	33.52326	37.50506
H	29.14561	36.22989	37.04646
H	31.25647	35.26631	40.58872
H	30.23889	38.37057	37.94260

Fe	31.51349	38.54327	40.95516
Cl	31.09729	40.45337	39.56477
C	33.74181	38.59575	42.79774
O	33.08460	39.29948	41.95213
O	34.77961	38.91109	43.37480
C	33.10739	37.20532	43.06808
O	32.15697	36.88537	42.35457
C	33.69626	36.30660	44.09079
C	34.75480	35.40792	43.43377
C	35.34230	34.36744	44.41062
O	36.04375	33.45653	43.90616
O	35.07074	34.52159	45.62740
H	34.15891	36.89257	44.89310
H	32.90261	35.68009	44.52038
H	34.32576	34.87302	42.57316
H	35.58492	36.01759	43.04004
N	27.73936	43.25861	41.74026
C	28.72594	42.36466	42.42837
C	27.94905	41.62761	43.55363
O	28.55719	40.76810	44.21543
C	29.93605	43.15025	42.95788
C	31.24653	42.35823	42.88424
C	32.44147	43.11627	43.48130
C	32.80402	44.43418	42.79122

N	33.70483	44.25154	41.60149
O	26.76353	42.00919	43.73463
H	27.01533	43.55693	42.43208
H	28.13781	44.11230	41.30155
H	27.27782	42.74405	40.97069
H	29.07626	41.64425	41.67777
H	30.03164	44.07493	42.36474
H	29.75354	43.47120	43.99621
H	31.11550	41.41049	43.42890
H	31.44339	42.06190	41.84013
H	32.22386	43.36205	44.53451
H	33.32582	42.45511	43.49757
H	31.91164	44.96774	42.43834
H	33.33796	45.10581	43.47448
H	33.62699	45.03960	40.91774
H	34.70455	44.21105	41.87704
H	33.52079	43.37594	41.07063
H	25.39322	38.23012	41.06195
H	28.45396	33.63404	38.82357

Figure 7b

C	25.95032	38.76360	41.82446
C	27.39107	38.38858	41.93200
N	27.85940	37.29645	42.62592
C	28.50813	38.97941	41.39587

C	29.19794	37.25133	42.50175
N	29.62292	38.27053	41.76832
H	25.91121	39.84887	41.64559
H	25.42295	38.58675	42.77196
H	27.27540	36.64198	43.15583
H	28.58557	39.84981	40.75187
H	29.83101	36.48876	42.94827
C	29.47773	33.77062	38.56262
C	29.98435	35.16470	38.74757
N	29.71145	36.20996	37.88282
C	30.78913	35.70828	39.72185
C	30.32467	37.31682	38.34760
N	30.99117	37.04617	39.46066
H	30.10749	33.07845	39.14398
H	29.56892	33.46714	37.50482
H	29.16139	36.16445	37.01743
H	31.22772	35.22900	40.59366
H	30.26117	38.30065	37.88646
Fe	31.51873	38.49329	40.92377
Br	31.12542	40.53820	39.42707
C	33.76259	38.53855	42.73867
O	33.11478	39.23495	41.87841
O	34.80764	38.84658	43.30456
C	33.10332	37.16656	43.04219

O	32.13941	36.85249	42.34368
C	33.68710	36.27630	44.07378
C	34.75215	35.38008	43.42396
C	35.34971	34.35312	44.40878
O	36.05419	33.44219	43.90858
O	35.08343	34.51758	45.62519
H	34.14674	36.86841	44.87353
H	32.89354	35.65019	44.50370
H	34.32653	34.83430	42.56861
H	35.57680	35.99337	43.02511
N	27.72651	43.26377	41.72935
C	28.71245	42.37931	42.42998
C	27.93063	41.63642	43.54752
O	28.54096	40.78315	44.21635
C	29.90833	43.17965	42.96980
C	31.23090	42.40668	42.91553
C	32.41386	43.20043	43.48997
C	32.76580	44.49411	42.75015
N	33.69188	44.28170	41.58636
O	26.74025	42.00642	43.71591
H	27.00086	43.56984	42.41570
H	28.12670	44.11335	41.28412
H	27.26733	42.74184	40.96318
H	29.07592	41.65943	41.68497

H	29.99944	44.10301	42.37415
H	29.71018	43.50211	44.00461
H	31.11344	41.47102	43.48404
H	31.43394	42.09133	41.87795
H	32.18880	43.48287	44.53208
H	33.30682	42.55254	43.53628
H	31.87117	44.99601	42.35879
H	33.27560	45.20177	43.41500
H	33.62876	45.05785	40.88760
H	34.68552	44.24484	41.88365
H	33.52027	43.39702	41.06616
H	25.38853	38.23037	41.05757
H	28.43255	33.60196	38.82190

Figure 7c

C	25.94750	38.77453	41.81840
C	27.38629	38.39553	41.94006
N	27.84037	37.30942	42.64927
C	28.51527	38.97801	41.41573
C	29.18427	37.26019	42.54336
N	29.62058	38.27248	41.81464
H	25.91041	39.85883	41.63147
H	25.41266	38.60611	42.76335
H	27.24803	36.66240	43.17753
H	28.61807	39.83615	40.75912

H	29.80842	36.49947	43.00530
C	29.52745	33.93321	38.57437
C	29.98306	35.34362	38.78016
N	29.66984	36.40238	37.94632
C	30.79985	35.88099	39.74989
C	30.27801	37.51099	38.43245
N	30.97394	37.22516	39.51961
H	30.18781	33.26365	39.14876
H	29.63834	33.64771	37.51294
H	29.11270	36.35560	37.08631
H	31.26475	35.38815	40.60033
H	30.18058	38.50817	38.00760
Fe	31.50295	38.71575	40.99151
F	31.01306	40.19540	39.90984
C	33.76840	38.65584	42.83231
O	33.11548	39.38060	42.00381
O	34.81173	38.95064	43.41133
C	33.12090	37.26482	43.08078
O	32.16747	36.96431	42.36605
C	33.70181	36.34862	44.09475
C	34.75002	35.43992	43.43487
C	35.32805	34.39038	44.40848
O	36.02514	33.47712	43.90141
O	35.05538	34.53991	45.62556

H	34.17077	36.92189	44.90257
H	32.89933	35.72938	44.51843
H	34.31489	34.91188	42.57306
H	35.58664	36.04121	43.04202
N	27.71483	43.24118	41.74534
C	28.70186	42.33649	42.41824
C	27.93805	41.62818	43.57116
O	28.55089	40.77908	44.24065
C	29.94716	43.09497	42.90052
C	31.22954	42.26153	42.79467
C	32.45475	42.96565	43.39901
C	32.82555	44.32097	42.78780
N	33.71538	44.19823	41.58047
O	26.75640	42.01889	43.75952
H	26.98696	43.51481	42.44445
H	28.10896	44.10768	41.32847
H	27.26116	42.74147	40.96179
H	29.01243	41.59355	41.67317
H	30.04989	44.01067	42.29453
H	29.80749	43.43065	43.94077
H	31.07467	41.31206	43.32967
H	31.39856	41.96548	41.74496
H	32.26362	43.14956	44.46992
H	33.32453	42.28710	43.35373

H	31.93672	44.88511	42.47580
H	33.37136	44.94764	43.50406
H	33.62099	45.00562	40.92196
H	34.71861	44.16361	41.84391
H	33.53190	43.33612	41.02938
H	25.38902	38.23691	41.05215
H	28.49189	33.70575	38.82722

Figure 8a

C	26.09690	38.61618	41.88475
C	27.54295	38.24829	41.95312
N	28.03905	37.13892	42.60025
C	28.64710	38.88694	41.43745
C	29.37988	37.12182	42.46626
N	29.77325	38.17515	41.76871
H	26.05117	39.71088	41.79764
H	25.59540	38.37365	42.83203
H	27.46309	36.45821	43.10686
H	28.70240	39.78618	40.83140
H	30.03226	36.36967	42.90320
C	29.55443	33.87444	38.57825
C	30.00224	35.28887	38.77059
N	29.66046	36.33147	37.92644
C	30.81307	35.85524	39.72689
C	30.23508	37.45904	38.39059

N	30.94124	37.20423	39.48131
H	30.20924	33.20915	39.16331
H	29.67128	33.57976	37.52013
H	29.10876	36.26633	37.06366
H	31.32652	35.38839	40.56266
H	30.12793	38.44144	37.93838
Fe	31.64950	38.55885	40.95740
Cl	31.28744	40.26533	39.41248
O	34.67539	38.15409	42.24689
O	32.11773	39.58543	42.10937
C	33.88196	37.22433	42.22471
O	32.82403	37.17661	41.43616
C	34.01944	35.99006	43.09741
C	35.35023	35.89561	43.83734
C	35.46665	34.58121	44.62234
O	35.96149	33.58189	44.03669
O	35.03281	34.60580	45.80423
H	33.18260	36.01501	43.81955
H	33.85876	35.10508	42.46003
H	36.17310	35.96563	43.11431
H	35.42330	36.73519	44.53982
N	27.57483	43.40528	41.70662
C	28.54756	42.49258	42.38246
C	27.69320	41.64657	43.36766

O	28.23864	41.21417	44.40983
C	29.72330	43.26725	42.97650
C	31.06539	42.54417	42.82321
C	32.24322	43.35116	43.38625
C	32.60663	44.60348	42.58322
N	33.65227	44.33997	41.53646
O	26.50365	41.49040	43.01711
H	27.09215	44.02989	42.38278
H	27.99874	44.02322	40.98788
H	26.86075	42.81801	41.25395
H	28.90949	41.82101	41.58869
H	29.77961	44.25134	42.48148
H	29.53509	43.46027	44.04372
H	31.00956	41.57088	43.33041
H	31.24079	42.29216	41.76223
H	32.00524	43.67351	44.41300
H	33.12917	42.70206	43.47018
H	31.73685	45.02460	42.05987
H	33.02053	45.38946	43.22604
H	33.64859	45.08531	40.80321
H	34.60841	44.33119	41.93846
H	33.53675	43.42763	41.05005
H	25.51150	38.14187	41.09710
H	28.51540	33.65539	38.82419

Figure 8b

C	26.04588	38.66062	41.86166
C	27.49633	38.30813	41.90972
N	28.02226	37.23350	42.59107
C	28.57935	38.93131	41.33478
C	29.36073	37.22695	42.42414
N	29.72127	38.25519	41.67445
H	25.98606	39.75392	41.76393
H	25.55857	38.42236	42.81705
H	27.46644	36.56239	43.13243
H	28.61678	39.79615	40.68092
H	30.03704	36.50727	42.87898
C	29.53346	33.87872	38.55852
C	29.98406	35.29401	38.73516
N	29.63907	36.33055	37.88326
C	30.79848	35.86726	39.68483
C	30.21471	37.46192	38.33589
N	30.92483	37.21045	39.42298
H	30.19020	33.21872	39.14734
H	29.64375	33.57255	37.50291
H	29.08256	36.25900	37.02428
H	31.30725	35.41157	40.52981
H	30.10332	38.44817	37.89392
Fe	31.55174	38.70327	40.76940

F	31.08647	39.83501	39.39406
O	34.49955	38.34257	42.25779
O	31.99960	39.90559	41.76054
C	33.71984	37.39970	42.24024
O	32.64154	37.34839	41.48880
C	33.91363	36.14136	43.07154
C	35.23131	36.09417	43.84024
C	35.39572	34.77015	44.60435
O	35.91298	33.79576	43.99555
O	34.97927	34.75908	45.79258
H	33.06062	36.07889	43.76979
H	33.82547	35.27668	42.39300
H	36.06618	36.21389	43.13626
H	35.25724	36.92630	44.55709
N	27.75684	43.33660	41.74707
C	28.56154	42.28333	42.44785
C	27.57223	41.57638	43.41032
O	28.04837	41.07946	44.46003
C	29.82998	42.79768	43.10377
C	30.84804	43.41939	42.14683
C	32.11060	43.87553	42.89296
C	32.91667	44.95683	42.18497
N	33.90213	44.42934	41.16949
O	26.37927	41.57731	43.04886

H	27.21020	43.92925	42.40448
H	28.28486	43.99044	41.13707
H	27.08187	42.85353	41.13449
H	28.83896	41.56336	41.66584
H	29.57952	43.50685	43.91037
H	30.27539	41.92187	43.59808
H	31.09205	42.68642	41.35854
H	30.40446	44.30007	41.64838
H	31.80643	44.30998	43.85643
H	32.75024	43.01213	43.14019
H	32.25394	45.64606	41.64297
H	33.50032	45.53842	42.90740
H	33.94299	45.08533	40.35942
H	34.85893	44.35818	41.56197
H	33.66336	43.48085	40.81502
H	25.46123	38.17234	41.08204
H	28.49533	33.66466	38.81255

Figure 8c

C	26.138870	38.584026	41.888897
C	27.592558	38.239372	41.918194
N	28.139221	37.152519	42.565773
C	28.663219	38.907951	41.376011
C	29.477982	37.188652	42.411125
N	29.827398	38.246841	41.690559

H	26.078083	39.680012	41.824211
H	25.662521	38.317760	42.842999
H	27.593412	36.448772	43.074577
H	28.670237	39.808372	40.772823
H	30.173897	36.477184	42.847008
C	29.510164	33.805115	38.540741
C	29.982004	35.211147	38.731030
N	29.656565	36.264950	37.896488
C	30.801180	35.755859	39.693546
C	30.253752	37.380463	38.366295
N	30.954561	37.098698	39.450115
H	30.161621	33.127880	39.115570
H	29.604328	33.509823	37.480946
H	29.094450	36.217316	37.038994
H	31.289454	35.268620	40.534100
H	30.152308	38.370712	37.927849
Fe	31.736008	38.478912	40.820435
Cl	30.994356	40.510250	39.926746
O	34.374344	38.429657	42.730309
O	33.176575	38.625999	40.111046
C	33.515804	37.578369	42.941612
O	32.338921	37.559025	42.358250
C	33.752922	36.445953	43.921898
C	34.819271	35.493164	43.382126

C	35.280209	34.407757	44.373158
O	35.950008	33.456131	43.896076
O	34.975414	34.569904	45.583641
H	34.101765	36.873112	44.873028
H	32.814270	35.913433	44.127544
H	34.470154	35.003609	42.460358
H	35.711990	36.074833	43.095627
N	27.623514	43.379211	41.687981
C	28.591227	42.502144	42.419384
C	27.726244	41.654434	43.391247
O	28.259052	41.241383	44.448948
C	29.716856	43.325993	43.045692
C	31.086962	42.638630	43.004322
C	32.223278	43.550972	43.485870
C	32.548092	44.721214	42.555645
N	33.592827	44.384109	41.531120
O	26.546238	41.478188	43.021202
H	27.057905	43.957325	42.340527
H	28.067850	44.042625	41.024246
H	26.986734	42.776264	41.145973
H	29.010340	41.827087	41.656395
H	29.780380	44.286335	42.507072
H	29.463055	43.567997	44.088638
H	31.046909	41.737125	43.634193

H	31.291592	42.272217	41.984310
H	31.961775	43.971535	44.468704
H	33.133572	42.954922	43.650700
H	31.665464	45.068565	42.000843
H	32.949902	45.572464	43.117222
H	33.613430	45.104595	40.773613
H	34.545788	44.366482	41.940983
H	33.456558	43.460232	41.072094
H	25.539181	38.122818	41.104214
H	28.471493	33.607792	38.805866

Figure 8d

C	26.066191	38.610336	41.905464
C	27.523832	38.287350	41.947159
N	28.095610	37.230965	42.624397
C	28.577826	38.970882	41.395912
C	29.435587	37.301769	42.478012
N	29.754534	38.356995	41.743023
H	25.996649	39.705433	41.833801
H	25.584925	38.344173	42.856804
H	27.564604	36.518341	43.136669
H	28.581045	39.848366	40.762245
H	30.150175	36.612740	42.920128
C	29.583303	34.037315	38.573177
C	29.970858	35.473747	38.742317

N	29.597683	36.493668	37.884415
C	30.781891	36.074409	39.678837
C	30.161135	37.641834	38.321316
N	30.886555	37.415806	39.400963
H	30.272169	33.415760	39.167313
H	29.720112	33.734180	37.519569
H	29.037741	36.398750	37.029831
H	31.296917	35.634785	40.528919
H	30.018677	38.612503	37.851238
Fe	31.572065	38.858772	40.836136
F	30.647690	40.340012	40.245964
O	34.290871	38.465878	42.624348
O	32.964581	39.299026	40.125767
C	33.498348	37.527935	42.587315
O	32.311504	37.579269	42.036137
C	33.784706	36.187477	43.251625
C	35.195930	36.048374	43.815990
C	35.371536	34.731743	44.596058
O	35.914402	33.756710	44.013279
O	34.934078	34.735332	45.777145
H	33.046021	36.077335	44.065571
H	33.571255	35.387825	42.522388
H	35.930756	36.103859	43.002533
H	35.371288	36.889133	44.503059

N	27.841503	43.272320	41.742943
C	28.685463	42.279049	42.488182
C	27.723707	41.550213	43.462112
O	28.219662	41.070370	44.510960
C	29.903059	42.900135	43.148026
C	30.925617	43.472218	42.164581
C	32.123688	44.100121	42.888168
C	32.895359	45.122765	42.069466
N	33.919426	44.515800	41.147038
O	26.526020	41.525078	43.112022
H	27.224613	43.820816	42.375298
H	28.355854	43.968304	41.169483
H	27.238646	42.755798	41.081543
H	29.040541	41.556953	41.738213
H	29.592007	43.670074	43.873352
H	30.355247	42.090782	43.738522
H	31.230551	42.679638	41.460213
H	30.451750	44.266907	41.560909
H	31.753248	44.632244	43.772999
H	32.817509	43.339687	43.281559
H	32.217491	45.710060	41.434177
H	33.443272	45.809822	42.723484
H	34.030888	45.147919	40.325127
H	34.847069	44.412075	41.599327

H	33.656387	43.574757	40.789131
H	25.479662	38.138783	41.116997
H	28.557951	33.762203	38.820217

Figure S28a-bi

C	25.88958	38.76020	41.76639
C	27.32237	38.37478	41.92097
N	27.74474	37.27407	42.62910
C	28.47126	38.93445	41.40956
C	29.08335	37.18455	42.53946
N	29.54776	38.18442	41.80812
H	25.86816	39.84385	41.58858
H	25.33687	38.59787	42.70098
H	27.13203	36.64215	43.15257
H	28.59248	39.80056	40.76673
H	29.69522	36.42824	43.02103
C	29.51173	33.80130	38.61666
C	30.01761	35.18420	38.87673
N	29.81946	36.26092	38.03042
C	30.74661	35.68389	39.92914
C	30.40745	37.34383	38.58344
N	30.98096	37.02847	39.73716
H	30.12962	33.08289	39.17894
H	29.62174	33.54572	37.54829
H	29.32206	36.24369	37.13272

H	31.09284	35.16997	40.82008
H	30.39746	38.34190	38.15366
Fe	31.56943	38.38991	41.29402
Cl	31.36844	40.11673	39.68807
O	33.56792	37.47724	41.51515
O	31.91837	39.43148	42.47253
C	33.04246	36.60824	42.27071
O	31.78668	36.66468	42.45869
C	33.77704	35.46458	42.92425
C	35.13556	35.76054	43.56572
C	35.52850	34.55474	44.44231
O	36.13295	33.59026	43.90533
O	35.14744	34.61610	45.64011
H	33.09596	35.07084	43.69225
H	33.89343	34.67976	42.15785
H	35.89107	35.94757	42.79106
H	35.04756	36.64032	44.21398
N	27.58555	43.33609	41.68952
C	28.54986	42.45489	42.42170
C	27.68952	41.64071	43.42787
O	28.24254	41.22272	44.47196
C	29.70228	43.26636	43.01273
C	31.06314	42.56845	42.91341
C	32.20520	43.45882	43.42195

C	32.55636	44.63384	42.50645
N	33.64195	44.30218	41.52088
O	26.49495	41.49895	43.09272
H	27.02582	43.91794	42.34407
H	28.03413	43.99760	41.02668
H	26.94108	42.74067	41.15017
H	28.94032	41.75510	41.66709
H	29.75221	44.23653	42.48975
H	29.48955	43.48998	44.06908
H	31.02940	41.63036	43.48605
H	31.25369	42.24832	41.87344
H	31.93321	43.87287	44.40517
H	33.10863	42.85405	43.58757
H	31.69261	44.97307	41.91738
H	32.93109	45.49077	43.07845
H	33.66898	45.01490	40.75639
H	34.58024	44.31451	41.96215
H	33.54404	43.36547	41.07840
H	25.34683	38.21716	40.99271
H	28.46310	33.62209	38.85400

Figure S28b-bi

C	25.94725	38.78906	41.75392
C	27.39119	38.42501	41.85723
N	27.85906	37.36251	42.59505

C	28.51393	38.98143	41.28381
C	29.19854	37.30151	42.46661
N	29.62016	38.28057	41.68317
H	25.89712	39.87408	41.58746
H	25.43092	38.61233	42.70685
H	27.27309	36.72861	43.14758
H	28.60140	39.81718	40.59762
H	29.84293	36.58446	42.96550
C	29.49015	33.75606	38.59344
C	30.01397	35.13614	38.82796
N	29.80810	36.20098	37.96751
C	30.76138	35.64848	39.86282
C	30.40878	37.28925	38.49302
N	30.99758	36.98349	39.63866
H	30.10111	33.03685	39.16208
H	29.58772	33.48242	37.52838
H	29.29681	36.17534	37.07789
H	31.12488	35.14798	40.75540
H	30.39915	38.28683	38.06373
Fe	31.63646	38.48596	40.98625
F	31.33689	39.59075	39.54068
O	33.71803	37.45508	41.15209
O	32.13238	39.69104	41.95758
C	33.15645	36.74776	42.02231

O	31.92391	36.97506	42.28563
C	33.78921	35.59077	42.76261
C	35.06801	35.90430	43.54951
C	35.45536	34.69149	44.42142
O	36.05304	33.72982	43.87114
O	35.10010	34.74739	45.62756
H	33.02669	35.21202	43.45855
H	33.99343	34.80169	42.02290
H	35.88274	36.15109	42.85461
H	34.88819	36.76129	44.21155
N	27.69711	43.32708	41.71326
C	28.49205	42.29474	42.45544
C	27.49570	41.61911	43.43155
O	27.97230	41.13132	44.48529
C	29.74934	42.83844	43.10889
C	30.79395	43.39316	42.13892
C	32.02036	43.93643	42.88625
C	32.83163	44.97382	42.12458
N	33.83660	44.38750	41.16124
O	26.30218	41.63165	43.07674
H	27.12038	43.90991	42.35335
H	28.24077	43.99127	41.12874
H	27.05600	42.83730	41.07016
H	28.77703	41.54497	41.70510

H	29.48368	43.59904	43.86134
H	30.17413	41.99382	43.67095
H	31.07517	42.59886	41.42604
H	30.35760	44.21807	41.54722
H	31.67385	44.43958	43.79930
H	32.66558	43.11296	43.22841
H	32.17731	45.62463	41.52756
H	33.40332	45.60178	42.81726
H	33.91079	45.01707	40.33325
H	34.78186	44.31930	41.58165
H	33.59367	43.42895	40.83824
H	25.38547	38.24863	40.99208
H	28.44019	33.59896	38.84035

Figure S28c-bi

C	26.12165	38.55409	41.91520
C	27.56170	38.15827	41.96487
N	28.03137	36.99432	42.53047
C	28.68121	38.80089	41.49210
C	29.36788	36.95031	42.38532
N	29.79739	38.03953	41.75967
H	26.09281	39.65110	41.84894
H	25.62271	38.30077	42.86154
H	27.44190	36.29722	42.99556
H	28.75443	39.73896	40.95381

H	29.99741	36.13592	42.72912
C	29.43670	33.76033	38.47641
C	29.92126	35.15991	38.67741
N	29.58064	36.24819	37.89278
C	30.75957	35.65874	39.64411
C	30.18990	37.34167	38.40163
N	30.90819	37.01172	39.46011
H	30.09299	33.07746	39.03897
H	29.51093	33.46673	37.41426
H	29.00743	36.23621	37.04218
H	31.26305	35.14565	40.45735
H	30.08489	38.35065	38.00932
Fe	31.72486	38.26681	40.94307
Cl	31.04975	40.35514	40.03244
O	32.56216	38.36120	42.88256
O	33.18786	38.40543	40.28376
C	32.67350	37.09335	42.94601
O	32.16181	36.41954	42.00612
C	33.41376	36.38612	44.05064
C	34.50557	35.48113	43.47213
C	35.17626	34.53369	44.48207
O	35.93957	33.65488	44.00351
O	34.91260	34.70236	45.70134
H	33.81226	37.10524	44.77470

H	32.68778	35.76396	44.59690
H	34.08546	34.86658	42.66257
H	35.29515	36.08836	42.99675
N	27.61637	43.36351	41.73868
C	28.59757	42.48286	42.44766
C	27.74552	41.62643	43.42389
O	28.28337	41.22165	44.48073
C	29.72682	43.30630	43.06502
C	31.09966	42.62489	43.01829
C	32.23091	43.56760	43.45113
C	32.53421	44.70015	42.46797
N	33.60416	44.34242	41.47517
O	26.56588	41.43908	43.05381
H	27.09022	43.97073	42.39790
H	28.04261	43.99840	41.03648
H	26.94256	42.75455	41.25256
H	29.00632	41.81606	41.67149
H	29.78442	44.26595	42.52400
H	29.47840	43.55012	44.10904
H	31.08501	41.73959	43.67150
H	31.29103	42.23059	42.00584
H	31.97442	44.02574	44.41782
H	33.14969	42.99041	43.63259
H	31.65136	44.99161	41.88212

H	32.90403	45.59066	42.98913
H	33.63011	45.04475	40.70098
H	34.54826	44.35426	41.90476
H	33.48901	43.40040	41.04911
H	25.51977	38.10434	41.12556
H	28.40226	33.56871	38.76153

Figure S28d-bi

C	26.08797	38.56100	41.94189
C	27.53255	38.18158	41.98932
N	28.03609	37.05006	42.59297
C	28.63279	38.84280	41.50112
C	29.37740	37.04830	42.45863
N	29.76865	38.13371	41.80837
H	26.05030	39.65852	41.87868
H	25.59217	38.30314	42.88866
H	27.46368	36.34504	43.06788
H	28.69463	39.75875	40.92676
H	30.03330	36.27002	42.83748
C	29.50707	33.98822	38.51411
C	29.91235	35.41727	38.69908
N	29.53295	36.47315	37.88810
C	30.74485	35.96997	39.64220
C	30.11664	37.59746	38.36384
N	30.85615	37.32233	39.42277

H	30.20058	33.35557	39.09083
H	29.62014	33.69160	37.45570
H	28.96234	36.41634	37.03767
H	31.27644	35.49243	40.45870
H	29.97942	38.58624	37.93103
Fe	31.61454	38.64249	40.95733
F	30.77897	40.25130	40.54561
O	32.56501	38.69699	42.90715
O	33.00810	39.03425	40.23678
C	32.74643	37.44030	42.86854
O	32.27984	36.81975	41.86688
C	33.47858	36.68199	43.95077
C	34.48147	35.67842	43.38056
C	35.12041	34.70923	44.39554
O	35.86870	33.81884	43.91246
O	34.85304	34.86891	45.61493
H	33.94918	37.38711	44.64597
H	32.72086	36.14007	44.54046
H	33.99877	35.07062	42.60045
H	35.30141	36.20461	42.86205
N	27.82193	43.29621	41.76134
C	28.64029	42.27598	42.49768
C	27.65015	41.56924	43.45768
O	28.11082	41.12558	44.53699

C	29.87782	42.85528	43.15726
C	30.92090	43.38359	42.16963
C	32.10231	44.04134	42.89314
C	32.87645	45.04652	42.05966
N	33.87861	44.41792	41.12181
O	26.46658	41.51967	43.06416
H	27.23647	43.87412	42.39723
H	28.34881	43.96502	41.16782
H	27.18791	42.78828	41.12402
H	28.97130	41.54954	41.74134
H	29.59207	43.63954	43.87787
H	30.30698	42.03793	43.75529
H	31.24251	42.55155	41.51923
H	30.45859	44.14405	41.51313
H	31.71719	44.60015	43.75587
H	32.77971	43.28239	43.31091
H	32.20168	45.64413	41.43043
H	33.44702	45.72694	42.70161
H	33.97271	45.03600	40.28736
H	34.81742	44.33670	41.55409
H	33.62056	43.46227	40.80147
H	25.48853	38.11248	41.14970
H	28.48435	33.72196	38.78095

IM1

C	26.09880	38.64694	41.88895
C	27.54708	38.28306	41.95073
N	28.05663	37.20819	42.64571
C	28.64487	38.90981	41.41040
C	29.39805	37.20637	42.52527
N	29.78316	38.23924	41.78974
H	26.04472	39.74164	41.79834
H	25.60499	38.40601	42.84050
H	27.48503	36.53260	43.16637
H	28.69389	39.77424	40.75585
H	30.05792	36.48493	43.00174
C	29.56442	33.97350	38.56599
C	29.99568	35.39456	38.75365
N	29.64584	36.44093	37.91629
C	30.82678	35.95640	39.69619
C	30.24019	37.56489	38.37486
N	30.96312	37.30167	39.44967
H	30.23674	33.32259	39.14755
H	29.68254	33.67960	37.50767
H	29.08547	36.37359	37.05897
H	31.34550	35.47996	40.52446
H	30.13246	38.55078	37.92801
C	37.94046	40.49825	42.56019

H	37.46139	39.54783	42.84121
H	37.78089	41.21971	43.37932
C	37.25274	41.10656	41.32498
O	37.60170	42.19937	40.84992
N	36.21797	40.41134	40.83946
H	35.58968	40.89093	40.19487
H	35.83745	39.56776	41.28163
Fe	31.63378	38.75641	40.97958
F	31.27292	40.05394	39.65389
O	34.91401	38.22633	42.08164
O	32.18366	40.07435	42.24201
C	34.01561	37.39545	42.16244
O	32.91172	37.45618	41.45490
C	34.08052	36.18665	43.07206
C	35.36332	36.07901	43.88834
C	35.44357	34.73892	44.63775
O	35.92484	33.75225	44.01999
O	35.00140	34.72986	45.81580
H	33.20024	36.23083	43.73714
H	33.94724	35.29612	42.43425
H	36.22662	36.16987	43.21566
H	35.38926	36.90418	44.61405
N	27.82936	43.33009	41.76209
C	28.58658	42.27025	42.49828

C	27.57515	41.58550	43.45424
O	28.04326	41.07239	44.50129
C	29.85690	42.76516	43.16941
C	30.98753	43.08000	42.24627
C	32.17427	43.84692	42.73599
C	32.75410	44.84162	41.72700
N	33.82593	44.26044	40.85411
O	26.38679	41.60528	43.08617
H	27.23404	43.90782	42.39107
H	28.40102	43.99883	41.20810
H	27.20592	42.86572	41.08229
H	28.86091	41.52756	41.73733
H	29.64094	43.62199	43.83022
H	30.12962	41.95156	43.87213
H	31.63470	40.87346	42.23493
H	31.03126	42.58792	41.26630
H	31.88877	44.41577	43.63374
H	32.98275	43.16605	43.07179
H	31.97306	45.22729	41.05765
H	33.20299	45.69234	42.25215
H	33.94430	44.90236	40.04344
H	34.73984	44.22352	41.34369
H	33.62060	43.27338	40.52824
O	33.45943	41.58813	40.33982

H	33.28001	41.06081	41.15538
H	32.76641	41.24989	39.73737
H	25.51007	38.17038	41.10514
H	28.53273	33.72869	38.81845
H	39.01428	40.31881	42.50748

TS_F

C	26.02600	38.64400	41.90600
C	27.47300	38.29000	42.01900
N	27.98400	37.25500	42.77000
C	28.57500	38.94800	41.53300
C	29.33000	37.31600	42.73700
N	29.71600	38.35300	42.00900
H	25.97700	39.73800	41.80400
H	25.50600	38.41500	42.84600
H	27.41200	36.56200	43.26500
H	28.61900	39.78400	40.84500
H	29.98900	36.63100	43.26500
C	29.70500	34.39400	38.67000
C	29.98700	35.85900	38.84700
N	29.60300	36.85400	37.96400
C	30.76200	36.50400	39.78600
C	30.12600	38.02600	38.40300
N	30.83300	37.85300	39.50800
H	30.42900	33.83300	39.28200

H	29.91000	34.11000	37.62200
H	29.07500	36.71700	37.09500
H	31.29800	36.08200	40.63100
H	29.98000	38.97400	37.88700
C	37.98300	40.53500	42.57800
H	37.49700	39.59700	42.88500
H	37.83000	41.28500	43.37000
C	37.32600	41.08900	41.30500
O	37.63000	42.19900	40.83600
N	36.39400	40.30800	40.75100
H	35.84700	40.71000	39.99400
H	35.98800	39.47800	41.20700
Fe	31.57800	39.07100	41.40700
F	31.08900	41.00700	40.81400
O	34.97100	38.30700	42.06700
O	32.37900	40.01900	42.84700
C	34.01500	37.52700	42.11800
O	32.92100	37.68300	41.43300
C	34.04200	36.28000	42.99000
C	35.31300	36.11700	43.81900
C	35.37600	34.77200	44.56499
O	35.85100	33.77900	43.95000
O	34.94200	34.76500	45.74599
H	33.15400	36.32400	43.64600

H	33.89500	35.41300	42.32300
H	36.18200	36.19700	43.15300
H	35.35600	36.93200	44.55399
N	27.71600	43.32900	41.80400
C	28.47500	42.16600	42.36900
C	27.55800	41.54000	43.45200
O	28.10200	41.05800	44.47499
C	29.87700	42.48100	42.83100
C	30.88200	42.79000	41.77100
C	32.19400	43.34600	42.26200
C	32.76500	44.49499	41.42200
N	33.99100	44.12500	40.64500
O	26.34200	41.58900	43.18400
H	27.30000	43.96000	42.52100
H	28.23200	43.93700	41.13700
H	26.92400	42.93600	41.27900
H	28.54600	41.45800	41.54000
H	29.86900	43.30600	43.56800
H	30.19400	41.60200	43.42100
H	32.03900	40.91800	42.85700
H	30.52800	43.13300	40.79900
H	32.02600	43.72900	43.28300
H	32.95900	42.56400	42.39300
H	32.03000	44.85999	40.69300

H	33.04300	45.34199	42.06200
H	34.10000	44.82799	39.88600
H	34.85000	44.17200	41.22500
H	33.94000	43.14200	40.27300
O	33.70100	41.37000	40.33500
H	33.90700	40.92400	41.17800
H	32.72800	41.26400	40.26700
H	25.45307	38.16350	41.11293
H	28.70599	34.00955	38.87555
H	39.05418	40.34100	42.52317

TS_{OH}

C	26.01600	38.63000	41.90800
C	27.46900	38.28700	41.96100
N	28.02000	37.20900	42.61600
C	28.53800	38.95400	41.41700
C	29.36000	37.24800	42.46000
N	29.70200	38.30800	41.74200
H	25.95700	39.72400	41.82400
H	25.52200	38.37900	42.85700
H	27.48000	36.51500	43.14300
H	28.52800	39.84400	40.79900
H	30.05100	36.53800	42.90800
C	29.55300	33.95400	38.58900
C	29.98300	35.37700	38.76900

N	29.69800	36.39400	37.87400
C	30.75200	35.96900	39.74600
C	30.27600	37.52900	38.32700
N	30.91800	37.30200	39.45900
H	30.21600	33.30300	39.18200
H	29.68400	33.65300	37.53400
H	29.17400	36.30600	36.99700
H	31.20300	35.51500	40.62600
H	30.22500	38.49500	37.83000
C	37.98000	40.50900	42.63100
H	37.50500	39.55800	42.91400
H	37.84900	41.20500	43.47800
C	37.23900	41.14900	41.44600
O	37.63400	42.19000	40.89300
N	36.08400	40.55900	41.12300
H	35.42800	41.05000	40.51500
H	35.71700	39.70600	41.56300
Fe	31.53000	38.88000	40.84900
F	31.64800	39.94800	39.27500
O	34.83700	38.34400	42.30300
O	31.22200	40.80300	41.65100
C	33.88500	37.56200	42.37200
O	32.72800	37.78900	41.82900
C	33.99300	36.23900	43.10800

C	35.29900	36.07800	43.88100
C	35.39000	34.73100	44.61499
O	35.87200	33.74900	43.99200
O	34.96200	34.71700	45.79899
H	33.12400	36.15900	43.78200
H	33.88300	35.43600	42.35700
H	36.14300	36.17500	43.18600
H	35.36500	36.88500	44.62499
N	27.81500	43.31000	41.77200
C	28.57200	42.25000	42.51100
C	27.54600	41.58200	43.45900
O	27.99400	41.08700	44.52299
C	29.84500	42.73400	43.17600
C	31.00000	42.96500	42.26000
C	32.25400	43.56900	42.82700
C	32.83000	44.72699	42.00100
N	33.84000	44.29399	40.98100
O	26.36400	41.59500	43.07000
H	27.23100	43.89700	42.40200
H	28.37400	43.97300	41.19900
H	27.17800	42.83900	41.11000
H	28.85800	41.49900	41.76800
H	29.64800	43.63900	43.78100
H	30.11200	41.96000	43.91300

H	30.95000	41.10100	40.76800
H	30.78500	43.19000	41.20900
H	32.02800	43.96500	43.83200
H	33.02700	42.80100	43.00100
H	32.03400	45.25799	41.45900
H	33.32600	45.44999	42.66000
H	33.87900	45.00599	40.22100
H	34.79200	44.23799	41.38700
H	33.63100	43.33500	40.57500
O	33.45800	41.69100	40.36200
H	32.84300	41.32200	41.04600
H	33.06500	41.33900	39.53900
H	25.43974	38.15378	41.11477
H	28.51682	33.71553	38.82887
H	39.05161	40.32688	42.55013

IM_F

C	26.07555	38.66600	41.87339
C	27.53439	38.34614	41.92080
N	28.08751	37.26458	42.56576
C	28.60867	39.03532	41.40409
C	29.43243	37.32729	42.43303
N	29.77819	38.40116	41.73993
H	25.99141	39.75836	41.78707
H	25.59428	38.41143	42.82801

H	27.54706	36.54704	43.06083
H	28.59897	39.92886	40.78765
H	30.12959	36.61238	42.86432
C	29.57580	34.04609	38.56484
C	29.96722	35.48546	38.69934
N	29.59891	36.48308	37.81658
C	30.79978	36.10329	39.60577
C	30.19135	37.63411	38.21061
N	30.92748	37.43839	39.29030
H	30.26445	33.43744	39.17241
H	29.71461	33.71989	37.51815
H	29.02993	36.37653	36.97006
H	31.32546	35.68113	40.45895
H	30.06290	38.58246	37.69104
C	37.99340	40.50250	42.69734
H	37.53113	39.55047	42.99616
H	37.87325	41.20152	43.54262
C	37.22068	41.12923	41.52531
O	37.55666	42.20512	41.00307
N	36.09844	40.48646	41.17172
H	35.42843	40.98560	40.57094
H	35.78919	39.59480	41.56811
Fe	31.68029	38.76031	40.83246
F	30.40000	43.00813	40.90489

O	34.62140	38.21973	42.20419
O	31.86208	40.56714	40.26662
C	33.77426	37.32892	42.35163
O	32.59449	37.36251	41.82110
C	34.03084	36.08924	43.18770
C	35.37512	36.05464	43.90292
C	35.53475	34.74173	44.68098
O	36.05667	33.76441	44.08162
O	35.08946	34.73327	45.85916
H	33.20777	36.01324	43.91919
H	33.92600	35.21733	42.51932
H	36.18532	36.15532	43.16986
H	35.42494	36.90621	44.59675
N	27.57268	43.41154	41.83106
C	28.37778	42.35994	42.51929
C	27.41174	41.64049	43.48320
O	27.90837	41.15032	44.52515
C	29.67551	42.84950	43.14604
C	30.50954	43.64944	42.16320
C	31.99284	43.77607	42.45128
C	32.57545	44.73233	41.41627
N	34.01608	44.51819	41.12671
O	26.22227	41.60607	43.11359
H	27.24997	44.16697	42.46913

H	28.02381	43.86725	41.01351
H	26.71480	42.93057	41.51697
H	28.62900	41.64411	41.72975
H	29.49386	43.44713	44.05175
H	30.21946	41.94923	43.46790
H	31.14478	41.20707	40.35495
H	30.06520	44.64787	42.01645
H	32.15640	44.13868	43.47849
H	32.43445	42.77118	42.36222
H	32.06317	44.58858	40.45723
H	32.43617	45.78113	41.71189
H	34.25387	45.16844	40.35087
H	34.60756	44.76268	41.94061
H	34.14563	43.50854	40.78913
O	33.92515	42.05089	40.08356
H	33.10699	41.43408	40.20484
H	33.92466	42.25510	39.11621
H	25.49000	38.18270	41.09133
H	28.54996	33.77407	38.81330
H	39.06395	40.32614	42.59297

IM_{OH}

C	26.03537	38.66037	41.86167
C	27.48337	38.29886	41.92665
N	28.00033	37.22391	42.61228

C	28.57545	38.92374	41.37124
C	29.34350	37.22481	42.46497
N	29.72073	38.25489	41.72532
H	25.98505	39.75343	41.75257
H	25.53638	38.43538	42.81459
H	27.43945	36.55208	43.14642
H	28.61390	39.78776	40.71577
H	30.01219	36.50375	42.93016
C	29.50225	33.90277	38.52811
C	29.93955	35.32694	38.66594
N	29.56935	36.34378	37.80580
C	30.79123	35.91961	39.57100
C	30.17607	37.48159	38.20884
N	30.92717	37.25568	39.27170
H	30.17250	33.26580	39.12688
H	29.61337	33.57415	37.47910
H	28.98471	36.26110	36.96752
H	31.31917	35.47483	40.41157
H	30.04116	38.44712	37.72518
C	37.99557	40.49074	42.66977
H	37.53106	39.53607	42.95654
H	37.86806	41.18248	43.52061
C	37.23328	41.12842	41.49768
O	37.60183	42.18398	40.95357

N	36.08609	40.52108	41.17442
H	35.41517	41.03670	40.59770
H	35.73633	39.65694	41.60360
Fe	31.54521	38.64680	40.74762
F	30.99239	40.25801	39.88085
O	34.72873	38.31705	42.31295
O	31.55321	41.89924	41.66594
C	33.85525	37.44404	42.35888
O	32.71774	37.52455	41.74531
C	34.03564	36.16567	43.15802
C	35.35579	36.06652	43.91424
C	35.47569	34.72879	44.65755
O	35.97998	33.75242	44.04175
O	35.03260	34.70807	45.83649
H	33.18519	36.09531	43.85777
H	33.92638	35.32242	42.45446
H	36.19031	36.17301	43.20890
H	35.40876	36.88772	44.64367
N	27.79581	43.28555	41.69209
C	28.59882	42.28098	42.46245
C	27.60170	41.60972	43.44659
O	28.07680	41.12776	44.50288
C	29.84197	42.87556	43.09864
C	31.01866	43.12566	42.13888

C	32.14166	43.90775	42.85160
C	32.82643	45.00418	42.03569
N	34.02085	44.56867	41.23634
O	26.41043	41.61542	43.08470
H	27.17520	43.84175	42.31532
H	28.33448	43.97703	41.13458
H	27.19852	42.78215	41.01573
H	28.89745	41.50470	41.74490
H	29.57647	43.80805	43.61763
H	30.16977	42.16522	43.87157
H	31.04359	41.43673	40.94954
H	30.65837	43.73588	41.28313
H	31.71537	44.41080	43.72921
H	32.87211	43.18204	43.24467
H	32.12297	45.45826	41.32217
H	33.18382	45.79550	42.70493
H	34.13466	45.24175	40.44991
H	34.89057	44.58607	41.79660
H	33.93860	43.59332	40.81943
O	33.81401	42.06879	40.22886
H	32.95841	41.83306	40.66523
H	33.65492	42.18068	39.26063
H	25.45207	38.16990	41.08240
H	28.46872	33.67594	38.78972

H	39.06745	40.31673	42.57574

IM2

C	26.08536	38.62142	41.91323
C	27.53486	38.26252	41.95026
N	28.06230	37.15934	42.58359
C	28.61911	38.91184	41.40854
C	29.40137	37.16570	42.41426
N	29.76675	38.22206	41.70610
H	26.03120	39.71739	41.83907
H	25.59910	38.36378	42.86466
H	27.50838	36.47330	43.10681
H	28.64202	39.82748	40.82753
H	30.07999	36.43363	42.84536
C	29.53929	33.92416	38.56241
C	29.96424	35.35048	38.71809
N	29.66219	36.35552	37.81681
C	30.74319	35.95528	39.67710
C	30.24138	37.49725	38.24222
N	30.89966	37.28628	39.36887
H	30.20596	33.28526	39.16329
H	29.66592	33.60799	37.51190
H	29.12321	36.26110	36.94821
H	31.20659	35.52189	40.55951
H	30.16697	38.44663	37.71517

C	37.88716	40.45491	42.66647
H	37.43781	39.50949	43.00460
H	37.75044	41.20140	43.46743
C	37.15910	40.99139	41.42313
O	37.50109	42.03762	40.85555
N	36.08867	40.27974	41.02886
H	35.55188	40.63374	40.24138
H	35.71451	39.44346	41.49341
Fe	31.68026	38.69976	40.76850
F	31.11980	40.49906	41.21294
O	34.58066	38.31253	42.42748
O	32.98390	39.30322	39.62722
C	33.73189	37.41922	42.50562
O	32.53321	37.51772	42.00789
C	33.98740	36.11062	43.22500
C	35.35094	36.01697	43.89907
C	35.49828	34.68528	44.64835
O	36.02408	33.71632	44.04109
O	35.04554	34.66671	45.82332
H	33.18287	35.98720	43.96929
H	33.85836	35.29675	42.48975
H	36.14436	36.11968	43.14757
H	35.44077	36.84147	44.62103
N	27.85959	43.35044	41.75189

C	28.64820	42.32834	42.50947
C	27.63254	41.60159	43.42488
O	28.07748	41.11357	44.49360
C	29.85974	42.89829	43.22700
C	30.88775	43.49840	42.33064
C	32.11928	44.11312	42.90699
C	32.80232	45.13367	41.99769
N	33.78298	44.50904	41.04429
O	26.45476	41.57687	43.01854
H	27.25214	43.92271	42.37284
H	28.41143	44.02621	41.18809
H	27.24764	42.85068	41.08680
H	29.02147	41.61037	41.76509
H	29.55437	43.61310	44.01336
H	30.28404	42.04084	43.78505
H	33.15954	40.25942	39.64749
H	30.86611	43.24757	41.26472
H	31.86544	44.61731	43.84981
H	32.85345	43.33355	43.20012
H	32.06180	45.66805	41.38692
H	33.35698	45.86600	42.59492
H	33.84973	45.10815	40.19530
H	34.73325	44.45004	41.45433
H	33.51974	43.51762	40.79142

O	33.28455	41.83485	40.89805
H	33.78702	41.42309	41.61995
H	32.37275	41.44326	41.01922
H	25.49511	38.15418	41.12496
H	28.50398	33.69002	38.81018
H	38.96130	40.29196	42.57855

TS_{iso}

C	26.06100	38.62600	41.91200
C	27.51200	38.27800	41.95400
N	28.05200	37.18100	42.58600
C	28.58800	38.94500	41.42000
C	29.39200	37.20800	42.42500
N	29.74400	38.27400	41.72200
H	26.00200	39.72100	41.83600
H	25.57200	38.36700	42.86200
H	27.50500	36.48500	43.10400
H	28.59400	39.85900	40.83600
H	30.07900	36.48300	42.85400
C	29.54600	33.94800	38.57100
C	29.96400	35.37500	38.73900
N	29.67700	36.38200	37.83500
C	30.72500	35.98000	39.71400
C	30.24700	37.52400	38.27500
N	30.88400	37.31300	39.41400

H	30.21500	33.30800	39.16900
H	29.67800	33.64000	37.51900
H	29.15000	36.28800	36.95800
H	31.17600	35.54200	40.60000
H	30.18600	38.47400	37.74800
C	37.88900	40.45800	42.66300
H	37.43700	39.51300	42.99700
H	37.75200	41.20400	43.46400
C	37.16800	40.99800	41.41700
O	37.51300	42.04400	40.85200
N	36.09700	40.28900	41.01700
H	35.56800	40.64500	40.22600
H	35.72000	39.45300	41.47900
Fe	31.64500	38.77200	40.82700
F	31.18600	40.58200	41.42000
O	34.60500	38.31600	42.39800
O	32.83900	39.38200	39.58400
C	33.73800	37.44200	42.49600
O	32.53300	37.56800	42.02000
C	33.98100	36.12800	43.21300
C	35.34200	36.02300	43.89100
C	35.48500	34.69100	44.64199
O	36.00900	33.72000	44.03600
O	35.03500	34.67500	45.81799

H	33.17200	36.00700	43.95300
H	33.85000	35.31900	42.47300
H	36.13800	36.12100	43.14100
H	35.43600	36.84700	44.61399
N	27.86800	43.34400	41.75500
C	28.63700	42.30000	42.50400
C	27.61300	41.59400	43.42300
O	28.05200	41.10200	44.49299
C	29.87200	42.82700	43.21000
C	30.94100	43.33400	42.30500
C	32.12600	44.03500	42.88600
C	32.77500	45.08299	41.98200
N	33.78600	44.50099	41.03500
O	26.43400	41.58300	43.01800
H	27.26800	43.92200	42.37800
H	28.43100	44.01400	41.19600
H	27.24800	42.86100	41.08500
H	28.98500	41.57700	41.75500
H	29.60200	43.58700	43.96700
H	30.24200	41.96700	43.80000
H	33.09200	40.32000	39.64400
H	30.93400	43.03700	41.25300
H	31.82100	44.54499	43.81000
H	32.90100	43.31000	43.21200

H	32.02000	45.59499	41.36900
H	33.29900	45.83299	42.58700
H	33.84700	45.10699	40.19100
H	34.73200	44.46599	41.45800
H	33.55800	43.50200	40.77800
O	33.35700	41.82100	40.89800
H	33.90700	41.42400	41.59100
H	32.45000	41.45200	41.11100
H	25.47817	38.15439	41.12082
H	28.51202	33.70446	38.81524
H	38.96306	40.29324	42.57746

IM3

C	26.10451	38.65293	41.88968
C	27.55715	38.30526	41.93811
N	28.08620	37.23100	42.61888
C	28.64247	38.94915	41.39220
C	29.42705	37.24854	42.48309
N	29.79344	38.29020	41.75008
H	26.04167	39.74787	41.80784
H	25.61959	38.40025	42.84274
H	27.52675	36.54135	43.13417
H	28.67092	39.82252	40.74933
H	30.09824	36.52654	42.94248
C	29.53964	33.91408	38.56730

C	29.98349	35.33055	38.75482
N	29.66641	36.36991	37.89675
C	30.78643	35.89736	39.71870
C	30.25575	37.49444	38.35885
N	30.93888	37.23719	39.46036
H	30.20188	33.25386	39.14994
H	29.65199	33.61789	37.50920
H	29.12191	36.30179	37.02948
H	31.27607	35.42411	40.56643
H	30.18209	38.47696	37.89731
C	37.90618	40.52683	42.56577
H	37.41368	39.58918	42.86397
H	37.74972	41.27634	43.35805
C	37.27531	41.08354	41.28126
O	37.63232	42.15826	40.78284
N	36.29320	40.34060	40.73415
H	35.92411	40.66748	39.84488
H	35.87593	39.49699	41.16180
Fe	31.63733	38.77325	40.86916
F	32.09254	40.08960	42.19904
O	34.97249	38.30322	42.08035
O	31.48353	39.98314	39.48372
C	34.06320	37.46786	42.11817
O	32.99482	37.53013	41.38058

C	34.10723	36.25369	43.02784
C	35.37928	36.11171	43.85627
C	35.43272	34.76558	44.60097
O	35.91333	33.77578	43.98770
O	34.97615	34.75762	45.77355
H	33.22254	36.31015	43.68694
H	33.95954	35.36962	42.38459
H	36.25162	36.19201	43.19377
H	35.41384	36.92837	44.59042
N	27.88777	43.32461	41.78616
C	28.63988	42.26589	42.53187
C	27.61602	41.57537	43.46703
O	28.06722	41.07172	44.52551
C	29.89777	42.76154	43.22477
C	30.97351	43.23521	42.31007
C	32.14892	43.97365	42.86158
C	32.75269	45.01086	41.91370
N	33.79418	44.42975	41.00287
O	26.43235	41.58612	43.08002
H	27.27502	43.89467	42.40486
H	28.46545	44.00134	41.24905
H	27.28118	42.85986	41.09077
H	28.94134	41.53057	41.77424
H	29.65528	43.52481	43.98450

H	30.25135	41.88616	43.80497
H	32.09604	40.73873	39.53642
H	31.05009	42.76426	41.32279
H	31.84840	44.49683	43.78020
H	32.94778	43.27036	43.17771
H	31.97928	45.45632	41.27297
H	33.23714	45.81233	42.48268
H	33.87345	45.03859	40.16221
H	34.72638	44.40803	41.45581
H	33.57969	43.42892	40.74054
O	33.45628	41.72598	40.78941
H	34.34124	41.36590	40.96423
H	32.89044	41.20867	41.43023
H	25.51623	38.17673	41.10531
H	28.50387	33.68883	38.82131
H	38.97778	40.33383	42.51568

TS3$_{OH}$

C	26.06300	38.62300	41.91400
C	27.51700	38.29200	42.00400
N	28.05900	37.26500	42.74500
C	28.59900	38.98100	41.51600
C	29.40300	37.36200	42.70600
N	29.75900	38.41600	41.98500
H	25.99600	39.71700	41.83400

H	25.56100	38.37000	42.85800
H	27.50500	36.55200	43.23400
H	28.62600	39.82000	40.83200
H	30.08300	36.69000	43.22600
C	29.70800	34.35700	38.66800
C	30.00700	35.81700	38.84900
N	29.61100	36.81700	37.97600
C	30.79800	36.45700	39.77900
C	30.14200	37.98700	38.41200
N	30.86500	37.80500	39.50400
H	30.42700	33.78300	39.27500
H	29.90500	34.07300	37.61800
H	29.07200	36.68600	37.11300
H	31.34100	36.03200	40.61900
H	29.98500	38.94200	37.91400
C	37.97700	40.55000	42.58800
H	37.48000	39.62000	42.90300
H	37.82200	41.31600	43.36400
C	37.36100	41.07000	41.28500
O	37.66600	42.16500	40.79700
N	36.46700	40.25300	40.69300
H	36.13700	40.53800	39.77500
H	36.04300	39.41200	41.12900
Fe	31.55900	39.18000	41.26200

F	32.45900	40.21400	42.67000
O	35.02400	38.33600	42.00900
O	31.00500	40.91500	40.55700
C	34.02900	37.60000	42.07300
O	32.93500	37.80400	41.41300
C	34.03200	36.34400	42.93900
C	35.30100	36.14400	43.76500
C	35.34700	34.79800	44.51299
O	35.82000	33.80100	43.90400
O	34.91000	34.79800	45.69399
H	33.14500	36.39500	43.59500
H	33.87100	35.48700	42.26100
H	36.17100	36.21000	43.09800
H	35.36200	36.95500	44.50499
N	27.74600	43.34200	41.85700
C	28.46800	42.13400	42.38100
C	27.51900	41.52100	43.43900
O	28.02800	41.06900	44.49399
C	29.87900	42.37000	42.86200
C	30.86200	42.84400	41.84800
C	32.09100	43.49600	42.42100
C	32.71900	44.61099	41.58500
N	33.92100	44.18000	40.80000
O	26.31000	41.55800	43.13400

H	27.40000	44.00100	42.58300
H	28.24700	43.91300	41.14800
H	26.90000	42.97900	41.39600
H	28.51100	41.46600	41.51900
H	29.87700	43.05600	43.73100
H	30.23800	41.41000	43.27100
H	31.77700	41.29600	40.10600
H	30.49600	43.21100	40.88500
H	31.78200	43.95900	43.37600
H	32.84100	42.74700	42.72600
H	31.99800	45.01799	40.86200
H	33.04300	45.43699	42.22900
H	34.02400	44.82799	39.99400
H	34.79000	44.25299	41.36000
H	33.84700	43.16800	40.51800
O	33.74000	41.44800	40.80500
H	34.62800	41.06400	40.89800
H	33.26600	41.07300	41.60100
H	25.48387	38.15321	41.11902
H	28.70503	33.98501	38.87715
H	39.04671	40.34771	42.53461

$TS3_F$

C	26.00000	38.68000	41.85800
C	27.45800	38.36700	41.92700

N	28.02800	37.33700	42.63900
C	28.51700	39.04100	41.37000
C	29.37100	37.41600	42.50800
N	29.69300	38.45700	41.75600
H	25.92400	39.77100	41.75200
H	25.50400	38.43900	42.80800
H	27.49800	36.63500	43.16700
H	28.50500	39.89200	40.70000
H	30.07500	36.74300	42.99200
C	29.57700	33.99900	38.61700
C	29.98400	35.42800	38.80200
N	29.70600	36.43600	37.89500
C	30.72500	36.03600	39.79200
C	30.26200	37.58100	38.35300
N	30.88300	37.36800	39.50100
H	30.24400	33.35700	39.21300
H	29.71900	33.70400	37.56200
H	29.19500	36.33700	37.01100
H	31.16200	35.59300	40.68400
H	30.21100	38.54800	37.85700
C	37.97300	40.54200	42.61000
H	37.48600	39.60800	42.92800
H	37.83300	41.30200	43.39600
C	37.32300	41.08500	41.33000

O	37.65600	42.16600	40.82700
N	36.35300	40.32000	40.80300
H	35.88200	40.68200	39.97700
H	35.93300	39.50500	41.27300
Fe	31.49400	39.04300	40.82800
F	31.38000	40.81900	42.00900
O	34.85200	38.50500	42.26800
O	31.42400	40.11900	39.32200
C	33.89600	37.71400	42.33000
O	32.74600	37.95100	41.79700
C	34.00100	36.38100	43.05900
C	35.31400	36.16200	43.80800
C	35.37600	34.80800	44.54399
O	35.86700	33.82100	43.93500
O	34.92700	34.79800	45.72099
H	33.14600	36.32000	43.75300
H	33.84600	35.58900	42.30400
H	36.15200	36.22900	43.10100
H	35.42000	36.95900	44.55599
N	27.84000	43.24600	41.75300
C	28.60000	42.19700	42.50800
C	27.59100	41.54700	43.48700
O	28.06600	41.03800	44.53299
C	29.87200	42.70000	43.15900

C	31.06300	42.81600	42.27200
C	32.26600	43.51400	42.83600
C	32.78700	44.66599	41.96200
N	33.89200	44.25499	41.03400
O	26.39600	41.59000	43.14000
H	27.21200	43.80100	42.37000
H	28.40300	43.93800	41.22200
H	27.24800	42.78000	41.04400
H	28.88400	41.43200	41.77900
H	29.68800	43.66200	43.67400
H	30.09300	41.98600	43.97000
H	32.08500	40.82400	39.27200
H	30.91200	42.84300	41.18900
H	32.00000	43.93900	43.81700
H	33.08800	42.80700	43.04400
H	31.98500	45.08499	41.33700
H	33.18000	45.47499	42.58900
H	33.92500	44.92299	40.23400
H	34.81700	44.29799	41.50100
H	33.77900	43.25600	40.71400
O	33.60200	41.52900	40.84500
H	34.29300	41.08400	41.36100
H	32.76500	41.28100	41.31600
H	25.43506	38.18128	41.07047

H	28.54245	33.74634	38.84924
H	39.04306	40.34399	42.54821

IM3$_F$

C	25.95938	38.69725	41.82554
C	27.41207	38.37022	41.92308
N	27.94778	37.29864	42.59863
C	28.49520	39.04429	41.41109
C	29.29390	37.34883	42.48352
N	29.65275	38.40621	41.77390
H	25.90192	39.78723	41.69794
H	25.44408	38.47802	42.77055
H	27.39783	36.59599	43.10408
H	28.51107	39.93709	40.79561
H	29.97734	36.63290	42.93488
C	29.54789	34.02089	38.57395
C	29.93165	35.46203	38.70753
N	29.57005	36.44610	37.80827
C	30.73158	36.10053	39.62965
C	30.13192	37.60893	38.20620
N	30.84415	37.43477	39.30559
H	30.22995	33.41381	39.19030
H	29.69693	33.69249	37.52963
H	29.01692	36.32678	36.95290
H	31.24111	35.69244	40.49950

H	29.99493	38.54815	37.67266
C	37.98530	40.47335	42.65751
H	37.52960	39.52445	42.97959
H	37.84451	41.21496	43.46190
C	37.26393	41.03356	41.42146
O	37.58256	42.11639	40.90894
N	36.23729	40.29894	40.96980
H	35.58679	40.76375	40.33523
H	35.84623	39.50033	41.47858
Fe	31.48755	38.81453	40.83597
F	31.42625	41.40985	42.98025
O	34.49327	38.49367	42.37929
O	31.39844	40.57804	40.06158
C	33.69414	37.54392	42.43747
O	32.54863	37.54448	41.84200
C	33.96508	36.27929	43.23432
C	35.35606	36.14722	43.84388
C	35.49213	34.81514	44.60197
O	36.03088	33.84348	44.00873
O	35.02058	34.79689	45.76933
H	33.20337	36.24013	44.03348
H	33.75338	35.42562	42.56830
H	36.11852	36.20683	43.05639
H	35.50847	36.97312	44.55203

N	27.74762	43.28557	41.68635
C	28.68018	42.39746	42.45240
C	27.78443	41.61591	43.45987
O	28.32131	41.17158	44.49976
C	29.85405	43.18330	43.04093
C	31.22446	42.72797	42.55244
C	32.35390	43.61147	43.09170
C	32.75038	44.73954	42.14013
N	33.83263	44.32100	41.19016
O	26.58802	41.51719	43.11645
H	27.13335	43.83555	42.32051
H	28.20808	43.97795	41.06407
H	27.14587	42.70230	41.08440
H	29.06469	41.66366	41.72970
H	29.75348	44.24985	42.78816
H	29.84027	43.10197	44.13798
H	31.24678	40.67677	39.11367
H	31.25138	42.67460	41.45204
H	32.05567	44.05034	44.05534
H	33.22570	42.97595	43.30171
H	31.89958	45.07679	41.52906
H	33.12582	45.60510	42.69778
H	33.85063	44.97444	40.37834
H	34.76636	44.37174	41.63767

H	33.72382	43.30785	40.87249
O	33.65153	41.66049	40.74482
H	33.78793	41.20365	41.59109
H	32.78350	41.26932	40.39472
H	25.40047	38.18500	41.04241
H	28.51871	33.75306	38.81295
H	39.05876	40.30271	42.57601

TS-R

C	25.95900	38.68800	41.84800
C	27.40900	38.34500	41.92300
N	27.94700	37.28800	42.62200
C	28.48400	38.97500	41.34700
C	29.28500	37.29900	42.46400
N	29.63400	38.32300	41.70100
H	25.90500	39.77900	41.72900
H	25.45300	38.46500	42.79700
H	27.39900	36.61300	43.16700
H	28.49900	39.83600	40.68700
H	29.96900	36.59600	42.93300
C	29.57000	33.99200	38.58300
C	29.98700	35.41800	38.76700
N	29.67400	36.44400	37.89100
C	30.76900	36.00400	39.73700
C	30.24700	37.57900	38.34900

N	30.91500	37.34200	39.46500
H	30.24200	33.34900	39.17300
H	29.70000	33.69600	37.52700
H	29.14300	36.35900	37.01700
H	31.24400	35.54900	40.60200
H	30.17000	38.55500	37.87500
C	37.96400	40.49400	42.64000
H	37.50000	39.54700	42.95200
H	37.83100	41.21800	43.46300
C	37.22800	41.08900	41.42700
O	37.59900	42.13800	40.87600
N	36.11200	40.44300	41.06500
H	35.46500	40.90100	40.42100
H	35.74300	39.61100	41.53300
Fe	31.48100	38.88300	40.93600
F	31.44100	39.99800	39.41300
O	34.58700	38.38000	42.28800
O	31.71300	40.23700	42.05700
C	33.73700	37.49200	42.32500
O	32.60600	37.55400	41.67600
C	33.91700	36.20700	43.11200
C	35.24600	36.11100	43.85400
C	35.38300	34.77700	44.60499
O	35.88800	33.80100	43.99000

O	34.95700	34.76500	45.78999
H	33.07400	36.13500	43.82000
H	33.80300	35.36900	42.40300
H	36.07200	36.21400	43.13700
H	35.30700	36.93300	44.57999
N	27.81400	43.26400	41.71300
C	28.60900	42.25800	42.48800
C	27.60900	41.57600	43.46100
O	28.08400	41.08200	44.51299
C	29.85800	42.85000	43.12700
C	31.13800	42.68100	42.32500
C	32.32500	43.46600	42.88300
C	32.75300	44.64099	42.00200
N	33.74200	44.22599	40.95200
O	26.42000	41.57600	43.09300
H	27.20000	43.83300	42.33100
H	28.35900	43.94600	41.15000
H	27.20800	42.76600	41.04000
H	28.90900	41.48500	41.76900
H	29.69700	43.92300	43.32200
H	29.97700	42.37900	44.11500
H	31.40600	41.45900	42.29400
H	30.99200	42.84100	41.24300
H	32.06300	43.86800	43.87400

H	33.19100	42.81200	43.06000
H	31.89300	45.08599	41.48000
H	33.22800	45.42499	42.60300
H	33.76100	44.94099	40.19300
H	34.70300	44.18700	41.34100
H	33.54700	43.26200	40.55900
O	33.44900	41.55700	40.34200
H	33.14300	41.08000	41.14600
H	32.81100	41.20700	39.68100
H	25.39607	38.18284	41.06314
H	28.53722	33.74348	38.82729
H	39.03669	40.31767	42.56061

TS-S

C	25.98200	38.65700	41.84900
C	27.43500	38.33200	41.92900
N	27.98700	37.25700	42.58800
C	28.50300	39.00900	41.39300
C	29.32600	37.29900	42.44200
N	29.66200	38.36100	41.72500
H	25.92300	39.74800	41.74300
H	25.47400	38.41900	42.79300
H	27.44700	36.55700	43.10800
H	28.50400	39.90100	40.77800
H	30.02000	36.58800	42.88400

C	29.58500	33.97600	38.59300
C	30.00100	35.40100	38.78500
N	29.69400	36.42700	37.90800
C	30.77400	35.98800	39.76300
C	30.26000	37.56300	38.37300
N	30.91900	37.32600	39.49400
H	30.25300	33.32800	39.18200
H	29.71900	33.68600	37.53600
H	29.16600	36.34200	37.03100
H	31.24500	35.53200	40.63100
H	30.18600	38.53900	37.89900
C	37.92200	40.48000	42.63100
H	37.45600	39.52800	42.92900
H	37.78200	41.20200	43.45400
C	37.19700	41.08300	41.41400
O	37.55200	42.15800	40.90600
N	36.12500	40.40400	40.99100
H	35.45600	40.88700	40.38700
H	35.73600	39.60000	41.49200
Fe	31.49000	38.92400	40.91900
F	31.45100	39.95100	39.36600
O	34.60600	38.40200	42.35300
O	31.70800	40.35200	41.96300
C	33.75200	37.51800	42.34700

O	32.62000	37.62000	41.70200
C	33.92800	36.19500	43.07400
C	35.25100	36.07100	43.82400
C	35.37900	34.73100	44.56999
O	35.88700	33.75500	43.95800
O	34.95200	34.71800	45.75399
H	33.08000	36.08700	43.77000
H	33.82300	35.39100	42.32400
H	36.08300	36.17500	43.11500
H	35.31000	36.88300	44.56099
N	27.65000	43.32000	41.76300
C	28.62900	42.39000	42.41100
C	27.77600	41.57100	43.42000
O	28.32500	41.15700	44.46699
C	29.81100	43.18000	42.96100
C	31.12100	42.44400	43.21900
C	32.34400	43.36000	43.33000
C	32.57500	44.41999	42.23500
N	33.68300	44.10600	41.28100
O	26.58100	41.42600	43.08400
H	27.23100	43.99200	42.43800
H	28.03300	43.89500	40.98700
H	26.87900	42.74100	41.40100
H	28.97100	41.71300	41.61600

H	29.99400	44.00800	42.25900
H	29.51100	43.64600	43.91500
H	31.03200	41.82200	44.12200
H	31.34600	41.49500	42.40900
H	32.23900	43.91100	44.28099
H	33.24900	42.74700	43.45400
H	31.68400	44.58899	41.61500
H	32.83400	45.37999	42.69900
H	33.70500	44.84199	40.54000
H	34.61400	44.13500	41.73600
H	33.58200	43.14800	40.82700
O	33.49500	41.57200	40.35900
H	32.99500	41.07500	41.05600
H	32.96700	41.38300	39.56000
H	25.42505	38.15927	41.05517
H	28.55067	33.72832	38.83152
H	38.99465	40.30157	42.55583

IM1-S

C	26.11488	38.63607	41.85539
C	27.57140	38.30740	41.90616
N	28.10613	37.21480	42.55487
C	28.65295	38.98024	41.38766
C	29.44563	37.24261	42.42239
N	29.80579	38.31164	41.72550

H	26.04021	39.72882	41.76978
H	25.63378	38.38262	42.81006
H	27.55171	36.51267	43.05859
H	28.68252	39.87406	40.77190
H	30.12456	36.51521	42.86118
C	29.56392	33.89783	38.55516
C	30.01268	35.31405	38.73317
N	29.65480	36.35609	37.89414
C	30.85120	35.88127	39.66693
C	30.24930	37.48230	38.34170
N	30.98241	37.22501	39.41105
H	30.22578	33.23933	39.14011
H	29.67370	33.59489	37.49842
H	29.08581	36.28889	37.04306
H	31.37732	35.41010	40.49380
H	30.13267	38.47000	37.90271
C	37.98636	40.54406	42.66221
H	37.50424	39.59515	42.94338
H	37.86129	41.25739	43.49384
C	37.26982	41.17349	41.45531
O	37.57766	42.29126	41.01294
N	36.25383	40.46165	40.95203
H	35.56937	40.98528	40.40245
H	35.88218	39.62064	41.40227

Fe	31.63132	38.78797	40.83465
F	31.13193	39.93892	39.45970
O	34.84833	38.29137	42.17060
O	32.18241	40.26993	41.92105
C	33.96739	37.43641	42.18773
O	32.87909	37.50647	41.46156
C	34.04878	36.18462	43.04236
C	35.33259	36.06094	43.85731
C	35.41827	34.71856	44.60147
O	35.90023	33.73194	43.98394
O	34.98587	34.70897	45.78395
H	33.16823	36.18339	43.70805
H	33.92982	35.32173	42.36523
H	36.19622	36.15600	43.18565
H	35.36444	36.87616	44.59214
N	27.49047	43.37972	41.70679
C	28.46574	42.53342	42.45631
C	27.62249	41.64292	43.40682
O	28.18497	41.18535	44.43109
C	29.51726	43.41622	43.13759
C	30.81314	42.72821	43.41560
C	32.15013	43.35790	43.18266
C	32.21098	44.24641	41.93431
N	33.61298	44.41610	41.46544

O	26.43596	41.48447	43.05754
H	26.92236	43.96011	42.35558
H	27.93382	44.04013	41.03774
H	26.86017	42.76232	41.17543
H	28.94451	41.88784	41.70467
H	29.68498	44.30579	42.51106
H	29.08103	43.78473	44.08397
H	30.76524	41.83295	44.03926
H	31.49962	40.96014	41.90544
H	32.46032	43.96259	44.06203
H	32.89708	42.55246	43.09533
H	31.66877	43.78137	41.09777
H	31.78387	45.24310	42.11164
H	33.65379	45.09763	40.67539
H	34.22818	44.79009	42.21440
H	33.93934	43.47009	41.11770
O	33.77117	41.90700	40.47311
H	33.22034	41.21806	40.94537
H	33.50123	41.94280	39.53197
H	25.52711	38.15270	41.07503
H	28.52786	33.67557	38.81061
H	39.05486	40.34505	42.57991

References

(1) Stemmer, W. P.; Morris, S. K. Enzymatic inverse PCR: a restriction site independent, single-fragment method for high-efficiency, site-directed mutagenesis. *Biotechniques* **1992**, *13* (2), 214-220.

(2) Ravi, N.; Bollinger, J. M., Jr.; Huynh, B. H.; Stubbe, J.; Edmondson, D. E. Mechanism of Assembly of the Tyrosyl Radical-Diiron(III) Cofactor of E. Coli Ribonucleotide Reductase: 1. Moessbauer Characterization of the Diferric Radical Precursor. *J. Am. Chem. Soc.* **1994**, *116* (18), 8007-8014. D

(3) Silakov, A. KazanViewer. **2022**.

(4) Stoll, S.; Schweiger, A. EasySpin, a comprehensive software package for spectral simulation and analysis in EPR. *J. Mag. Res.* **2006**, *178* (1), 42-55.

(5) Finkelstein, K. D.; Pollock, C. J.; Lyndaker, A.; Krawcyk, T.; Conrad, J. Dual-array valence emission spectrometer (DAVES): A new approach for hard x-ray photon-in photon-out spectroscopies. *AIP Conf. Proc.* **2016**, *1741* (1), 030009.

(6) Sole, V. A.; Papillon, E.; Cotte, M.; Walter, P.; Susini, J. A multiplatform code for the analysis of energy-dispersive X-ray fluorescence spectra. *Spectroc. Acta Pt. B-Atom. Spectr.* **2007**, *62* (1), 63-68, Article.

(7) Ravel, B.; Newville, M. ATHENA, ARTEMIS, HEPHAESTUS: data analysis for X-ray absorption spectroscopy using IFEFFIT. *J Synchrotron Radiat* **2005**, *12* (Pt 4), 537-541.

(8) Delgado-Jaime, M. U.; Mewis, C. P.; Kennepohl, P. Blueprint XAS: a Matlab-based toolbox for the fitting and analysis of XAS spectra. *J. Synchrotron Radiat.* **2010**, *17* (1), 132-137.

(9) Neugebauer, M. E.; Sumida, K. H.; Pelton, J. G.; McMurry, J. L.; Marchand, J. A.; Chang, M. C. Y. A family of radical halogenases for the engineering of amino-acid-based products. *Nat. Chem. Biol.* **2019**, *15* (10), 1009-1016.

(10) Case, D. A. B., S. R.; Cerutti, D. S.; Cheatham, T. E.; Cruzeiro, I. V. W. D.; Darden, T. A.; Duke, R. E.; Ghoreishi, D.; Gilson, M. K.; Gohlke, H.; Goetz, A. W.; Greene, D.; Harris, R.; Homeyer, N.; Izadi, S.; Kovalenko, A.; Kurtzman, T.; Lee, T. S.; LeGrand, S.; Li, P.; Lin, C.; Liu, J.; Luchko, T.; Luo, R.; Mermelstein, D. J.; Merz, K. M.; Miao, Y.; Monard, G.; Nguyen, C.; Nguyen, H.; Omelyan, I.; Onufriev, A.; Pan, F.; Qi, R.; Roe, D. R.; Roitberg, A.; Sagui, C.; Schott-Verdugo, S.; Shen, J.; Simmerling, C. L.; Smith, J.; Salomon-Ferrer, R.; Swails, J.; Walker, R. C.; Wang, J.; Wei, H.; Wolf, R. M.; Wu, X.; Xiao, L.; York, D. M.; Kollman, P. A. *AMBER 2018*; University of California, San Francisco, 2018.

(11) Søndergaard, C. R.; Olsson, M. H. M.; Rostkowski, M.; Jensen, J. H. Improved Treatment of Ligands and Coupling Effects in Empirical Calculation and Rationalization of pKa Values. *J. Chem. Theory. Comput.* **2011**, *7* (7), 2284-2295.

(12) Wang, J.; Wolf, R. M.; Caldwell, J. W.; Kollman, P. A.; Case, D. A. Development and testing of a general amber force field. *J. Comput. Chem.* **2004**, *25* (9), 1157-1174.

(13) Bayly, C. I.; Cieplak, P.; Cornell, W.; Kollman, P. A. A well-behaved electrostatic potential based method using charge restraints for deriving atomic charges: the RESP model. *J. Phys. Chem.* **1993**, *97* (40), 10269-10280.

(14) Roothaan, C. C. J. New Developments in Molecular Orbital Theory. *Rev. Mod. Phys.* **1951**, *23* (2), 69-89.

(15) Hehre, W. J.; Ditchfield, R.; Pople, J. A. Self—Consistent Molecular Orbital Methods. XII. Further Extensions of Gaussian—Type Basis Sets for Use in Molecular Orbital Studies of Organic Molecules. *J. Chem. Phys.* **1972**, *56* (5), 2257-2261.

(16) Hariharan, P. C.; Pople, J. A. The influence of polarization functions on molecular orbital hydrogenation energies. *Theor. Chim. Acta* **1973**, *28* (3), 213-222.

(17) Frisch, M. J. T., G. W.; Schlegel, H. B.; Scuseria, G. E.; Robb, M. A.; Cheeseman, J. R.; Scalmani, G.; Barone, V.; Petersson, G. A.; Nakatsuji, H.; Li, X.; Caricato, M.; Marenich, A. V.; Bloino, J.; Janesko, B. G.; Gomperts, R.; Mennucci, B.; Hratchian, H. P.; Ortiz, J. V.; Izmaylov, A. F.; Sonnenberg, J. L.; Ding, F.; Lipparini, F.; Egidi, F.; Goings, J.; Peng, B.; Petrone, A.; Henderson, T.; Ranasinghe, D.; Zakrzewski, V. G.; Gao, J.; Rega, N.; Zheng, G.; Liang, W.; Hada, M.; Ehara, M.; Toyota, K.; Fukuda, R.; Hasegawa, J.; Ishida, M.; Nakajima, T.; Honda, Y.; Kitao, O.; Nakai, H.; Vreven, T.; Throssell, K.; Montgomery, J. A., Jr.; Peralta, J. E.; Ogliaro, F.; Bearpark, M. J.; Heyd, J. J.; Brothers, E. N.; Kudin, K. N.; Staroverov, V. N.; Keith, T. A.; Kobayashi, R.; Normand, J.; Raghavachari, K.; Rendell, A. P.; Burant, J. C.; Iyengar, S. S.; Tomasi, J.; Cossi, M.; Millam, J. M.; Klene, M.; Adamo, C.; Cammi, R.; Ochterski, J. W.; Martin, R. L.; Morokuma, K.; Farkas, O.; Foresman, J. B.; Fox, D. J. *Gaussian 16 Rev. C.01*; Gaussian Inc., 2016.

(18) Maier, J. A.; Martinez, C.; Kasavajhala, K.; Wickstrom, L.; Hauser, K. E.; Simmerling, C. ff14SB: Improving the Accuracy of Protein Side Chain and Backbone Parameters from ff99SB. *J. Chem. Theory Comput.* **2015**, *11* (8), 3696-3713.

(19) Li, P.; Merz, K. M., Jr. MCPB.py: A Python Based Metal Center Parameter Builder. *J. Chem. Inf. Model.* **2016**, *56* (4), 599-604.

(20) Li, P.; Merz, K. M., Jr. Metal Ion Modeling Using Classical Mechanics. *Chem. Rev.* **2017**, *117* (3), 1564-1686.

(21) Zhang, J. The Hybrid Method of Steepest Descent: Conjugate Gradient with Simulated Annealing. In *Molecular Structures and Structural Dynamics of Prion Proteins and Prions: Mechanism Underlying the Resistance to Prion Diseases*, Zhang, J. Ed.; Springer Netherlands, 2015; pp 171-201.
(22) Brooks, C. L. Computer simulation of liquids. *J. Solut. Chem.* **1989**, *18* (1), 99-99.
(23) van Gunsteren, W. F.; Berendsen, H. J. C. Computer Simulation of Molecular Dynamics: Methodology, Applications, and Perspectives in Chemistry. *Angew. Chem. Int. Ed.* **1990**, *29* (9), 992-1023.
(24) Kräutler, V.; Gunsteren, W. F. v.; Hünenberger, P. H. A fast SHAKE algorithm to solve distance constraint equations for small molecules in molecular dynamics simulations. *J. Comput. Chem.* **2001**, *22*.
(25) Metz, S.; Kästner, J.; Sokol, A. A.; Keal, T. W.; Sherwood, P. ChemShell—a modular software package for QM/MM simulations. *WIREs Comput. Mol. Sci.* **2014**, *4* (2), 101-110.
(26) de Vries, A. H.; Sherwood, P.; Collins, S. J.; Rigby, A. M.; Rigutto, M.; Kramer, G. J. Zeolite Structure and Reactivity by Combined Quantum-Chemical−Classical Calculations. *J. Phys. Chem. B* **1999**, *103* (29), 6133-6141.
(27) Smith, W.; Yong, C. W.; Rodger, P. M. DL_POLY: Application to molecular simulation. *Mol. Simul.* **2002**, *28* (5), 385-471.
(28) Furche, F.; Ahlrichs, R.; Hättig, C.; Klopper, W.; Sierka, M.; Weigend, F. Turbomole. *WIREs Comput. Mol. Sci.* **2014**, *4* (2), 91-100.
(29) Bakowies, D.; Thiel, W. Hybrid Models for Combined Quantum Mechanical and Molecular Mechanical Approaches. *J. Phys. Chem.* **1996**, *100* (25), 10580-10594.
(30) Sherwood, P.; de Vries, A. H.; Guest, M. F.; Schreckenbach, G.; Catlow, C. R. A.; French, S. A.; Sokol, A. A.; Bromley, S. T.; Thiel, W.; Turner, A. J.; et al. QUASI: A general purpose implementation of the QM/MM approach and its application to problems in catalysis. *Journal of Molecular Structure: THEOCHEM* **2003**, *632* (1), 1-28.
(31) Lee, C.; Yang, W.; Parr, R. G. Development of the Colle-Salvetti correlation-energy formula into a functional of the electron density. *Phys. Rev. B* **1988**, *37* (2), 785-789.
(32) Becke, A. D. Density-functional thermochemistry. II. The effect of the Perdew–Wang generalized-gradient correlation correction. *J. Chem. Phys.* **1992**, *97* (12), 9173-9177.
(33) Becke, A. D. Density-functional thermochemistry. III. The role of exact exchange. *J. Chem. Phys.* **1993**, *98* (7), 5648-5652.

(34) Weigend, F.; Ahlrichs, R. Balanced basis sets of split valence, triple zeta valence and quadruple zeta valence quality for H to Rn: Design and assessment of accuracy. *Phys. Chem. Chem. Phys.* **2005**, *7* (18), 3297-3305.
(35) Caldeweyher, E.; Bannwarth, C.; Grimme, S. Extension of the D3 dispersion coefficient model. *J. Chem. Phys.* **2017**, *147* (3).
(36) Kästner, J.; Carr, J. M.; Keal, T. W.; Thiel, W.; Wander, A.; Sherwood, P. DL-FIND: An Open-Source Geometry Optimizer for Atomistic Simulations. *J. Phys. Chem. A* **2009**, *113* (43), 11856-11865.
(37) Wang, C.; Chen, H. Convergent Theoretical Prediction of Reactive Oxidant Structures in Diiron Arylamine Oxygenases AurF and CmlI: Peroxo or Hydroperoxo? *J. Am. Chem. Soc.* **2017**, *139* (37), 13038-13046.
(38) Peng, W.; Yan, S.; Zhang, X.; Liao, L.; Zhang, J.; Shaik, S.; Wang, B. How Do Preorganized Electric Fields Function in Catalytic Cycles? The Case of the Enzyme Tyrosine Hydroxylase. *J. Am. Chem. Soc.* **2022**, *144* (44), 20484-20494.
(39) Peng, W.; Wang, Z.; Zhang, Q.; Yan, S.; Wang, B. Unraveling the Valence State and Reactivity of Copper Centers in Membrane-Bound Particulate Methane Monooxygenase. *J. Am. Chem. Soc.* **2023**, *145* (46), 25304-25317.
(40) Lu, J.; Bi, B.; Lai, W.; Chen, H. Origin of Nitric Oxide Reduction Activity in Flavo–Diiron NO Reductase: Key Roles of the Second Coordination Sphere. *Angew. Chem. Intl. Ed.* **2019**, *58* (12), 3795-3799.
(41) Lu, J.; Lai, W.; Chen, H. C(sp^3)−H Hydroxylation in Diiron β-Hydroxylase CmlA Transpires by Amine-Assisted O_2 Activation Avoiding $Fe^{IV}{}_2O_2$ Species. *Angew. Chem. Intl. Ed.* **2022**, *61* (46), e202211843.
(42) Zhang, J.; Li, Y.; Yuan, W.; Zhang, X.; Si, Y.; Wang, B. Conformational Isomerization of the Fe(III)–OH Species Enables Selective Halogenation in Carrier-Protein-Independent Halogenase BesD and Hydroxylase-Evolved Halogenase. *ACS Catal.* **2024**, *14* (12), 9342-9353.
(43) Humphrey, W.; Dalke, A.; Schulten, K. VMD: Visual molecular dynamics. *J. Mol. Graph.* **1996**, *14* (1), 33-38..
(44) Legault, C. Y. CYLview, 1.0b. Université de Sherbrooke: 2009.
(45) Neese, F. Software Update: The ORCA Program System—Version 6.0. *WIREs Comput. Mol. Sci.* **2025**, *15* (2), e70019.
(46) Grimme, S. Accurate Calculation of the Heats of Formation for Large Main Group Compounds with Spin-Component Scaled MP2 Methods. *J. Phys. Chem A.,* **2005**, *109* (13), 3067-3077. DOI: 10.1021/jp050036j.

(47) Liu, W. Big picture of relativistic molecular quantum mechanics. *Nat. Sci. Rev.* **2015**, *3* (2), 204-221.
(48) Helmich-Paris, B.; de Souza, B.; Neese, F.; Izsák, R. An improved chain of spheres for exchange algorithm. *J. Chem. Phys.* **2021**, *155* (10).
(49) Neese, F.; Wolf, A.; Fleig, T.; Reiher, M.; Hess, B. A. Calculation of electric-field gradients based on higher-order generalized Douglas–Kroll transformations. *J. Chem. Phys.* **2005**, *122* (20).
(50) Mastalerz, R.; Barone, G.; Lindh, R.; Reiher, M. Analytic high-order Douglas-Kroll-Hess electric field gradients. *J. Chem. Phys.* **2007**, *127* (7).
(51) Autschbach, J.; Peng, D.; Reiher, M. Two-Component Relativistic Calculations of Electric-Field Gradients Using Exact Decoupling Methods: Spin–orbit and Picture-Change Effects. *J. Chem. Theory Comput.* **2012**, *8* (11), 4239-4248.
(52) Barone, G.; Mastalerz, R.; Reiher, M.; Lindh, R. Nuclear Quadrupole Moment of ^{119}Sn. *J. Phys. Chem. A.,* **2008**, *112* (7), 1666-1672.
(53) Gómez.-Piñeiro, R. J.; Pantazis, D. A.; Orio, M. Comparison of Density Functional and Correlated Wave Function Methods for the Prediction of Cu(II) Hyperfine Coupling Constants. *ChemPhysChem* **2020**, *21* (24), 2667-2679.
(54) Pollak, P.; Weigend, F. Segmented Contracted Error-Consistent Basis Sets of Double- and Triple-ζ Valence Quality for One- and Two-Component Relativistic All-Electron Calculations. *J. Chem. Theory Comput*. **2017**, *13* (8), 3696-3705.
(55) Santra, G.; Neese, F.; Pantazis, D. A. Extensive reference set and refined computational protocol for calculations of ^{57}Fe Mössbauer parameters. *Phys. Chem. Chem. Phys.* **2024**, *26* (35), 23322-23334.
(56) DeBeer George, S.; Petrenko, T.; Neese, F. Prediction of iron K-edge absorption spectra using time-dependent density functional theory *J. Phys. Chem. A* **2008**, *112*, 12936–12943.
(57) Neese, F. The ORCA program system. *WIREs Comput. Mol. Sci.* **2012**, *2* (1), 73-78.
(58) Neese, F. Software update: The ORCA program system—Version 5.0. *WIREs Comput. Mol. Sci.* **2022**, e1606.
(59) Becke, A. D. *Phys. Rev. A.* **1988**, *38*, 3098.
(60) Perdew, J. P. *Phys. Rev. B.* **1986**, *33*, 8822.
(61) Weigend, F.; Ahlrichs, R. Balanced basis sets of split valence, triple zeta valence and quadruple zeta valence quality for H to Rn: Design and assessment of accuracy. *Phys. Chem. Chem. Phys.* **2005**, *7* (18), 3297-3305.

(62) Neese, F. Prediction and interpretation of the ^{57}Fe isomer shift in Mossbauer spectra by density functional theory. *Inorg. Chim. Acta* **2002**, *337C*, 181.
(63) Barone, V.; Cossi, M. Quantum Calculation of Molecular Energies and Energy Gradients in Solution by a Conductor Solvent Model. *J. Phys. Chem. A.,* **1998**, *102* (11), 1995-2001.
(64) Chandrasekaran, P.; Stieber, S. C. E.; Collins, T. J.; Que, J. L.; Neese, F.; DeBeer, S. Prediction of high-valent iron K-edge absorption spectra by time-dependent Density Functional Theory. *Dalton Trans.* **2011**, *40* (42), 11070-11079.
(65) Hugenbruch, S.; Shafaat, H. S.; Kramer, T.; Delgado-Jaime, M. U.; Weber, K.; Neese, F.; Lubitz, W.; DeBeer, S. In search of metal hydrides: an X-ray absorption and emission study of [NiFe] hydrogenase model complexes. *Phys. Chem. Chem. Phys.* **2016**, *18*, 10688-10699.
